# Carbon Efficient CO_2_ Interfaces in Acid through Ion Management Channels

**DOI:** 10.1021/acsenergylett.5c02981

**Published:** 2025-12-15

**Authors:** Blanca Belsa, Anku Guha, Barbara Polesso, Ranit Ram, Viktoria Golovanova, Marinos Dimitropoulos, Sunil Kadam, Prathama Haldar, Aliaksandr S. Bandarenka, F. Pelayo García de Arquer

**Affiliations:** † 172281 ICFO - Institut de Ciències Fotòniques, The Barcelona Institute of Science and Technology, Castelldefels (Barcelona) 08860, Spain; ‡ Physik-Department ECS, 84665 Technische Universität München, James-Franck-Str. 1, Garching D-85748, Germany; § Catalysis Research Center TUM, Ernst-Otto-Fischer-Straße 1, Garching bei München 85748, Germany

## Abstract

CO_2_ electroreduction (CO_2_E) in acidic media enables high carbon utilization but is often limited by enhanced hydrogen evolution (HER). Cation-exchange ionomer coatings can suppress HER by enriching alkali cations at the catalyst surface, yet *in situ* SERS reveals that they also cause excessive *OH adsorption, hindering CO_2_ access and suppressing C–C coupling. To address this, we introduce ion management channels (IMCs), a spatially distributed ionomer architecture combining cation- and anion-exchange domains to modulate nanoscale ion transport. Applied to PTFE-Cu gas diffusion electrodes, IMCs facilitate the removal of excess *OH by restructuring interfacial water, increasing *CO coverage, and enhancing multicarbon (C_2+_) selectivity. IMC-functionalized electrodes achieve ∼80% Faradaic efficiency for C_2+_ products at 0.5 A·cm^–2^, with ∼90% single-pass carbon utilization sustained over 70 h of pulsed operation. This represents a 39% improvement in peak C_2+_ partial current density over monopolar cation-exchange ionomers and a ∼3.4-fold enhancement relative to bare Cu, highlighting the importance of ion-transport engineering for efficient CO_2_E.

## Graphical abstract

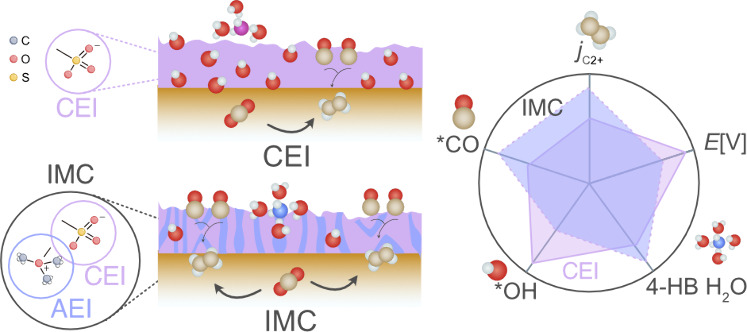

Carbon dioxide electroreduction (CO_2_E) offers a sustainable pathway to produce widely used fuels and chemicals from CO_2_ gas. Examples include the generation of carbon monoxide, syngas, formate, methane, ethylene, and ethanol, whose production represents >0.4 Gt_CO2_ emissions globally and a multibillion-dollar market.^1^ For CO_2_E to reach technoeconomic and environmental viability, performance metrics such as selectivity, current density, energy, carbon efficiency, and stability must improve simultaneously. These metrics are crucial not only for CO_2_E but also to enable energy and carbon efficiency in the full capture and utilization process, including downstream product separation.^2^

Early progress in CO_2_E focused on improving selectivity and reaction rate, achieving notable success in producing CO, formate, and higher-value C_2_ products like ethylene.^3^ This progress has largely relied on the adoption of flow cell reactors, gas-diffusion electrodes, and alkaline electrolytes.^4^ These were followed by the increasing implementation of membrane electrode assemblies (MEAs) with anion exchange membranes (AEMs) and neutral-to-alkaline anolytes, presenting a scalable approach with competitive energy efficiency.^5^

These advances are still challenged by the undesired consumption of CO_2_ into (bi)­carbonate species in locally alkaline environments. This contributes to salt formation, compromising operational stability and leading to low carbon utilization. The latter imposes additional separation steps to recover/separate CO_2_, increasing the energy intensity of the full process and compromising viability.^6,7^

One approach to circumventing carbonate formation is implementing CO_2_E in acid electrolytes. Such operation enables local proton-driven regeneration of carbonates into CO_2_, which could then be reduced at the electrocatalyst, thus enabling high carbon efficiency.^8,9^ However, this mode of operation also favors the competing hydrogen evolution reaction (HER) over the CO_2_E (Figure 1a).^10,11^

**Figure 1:**
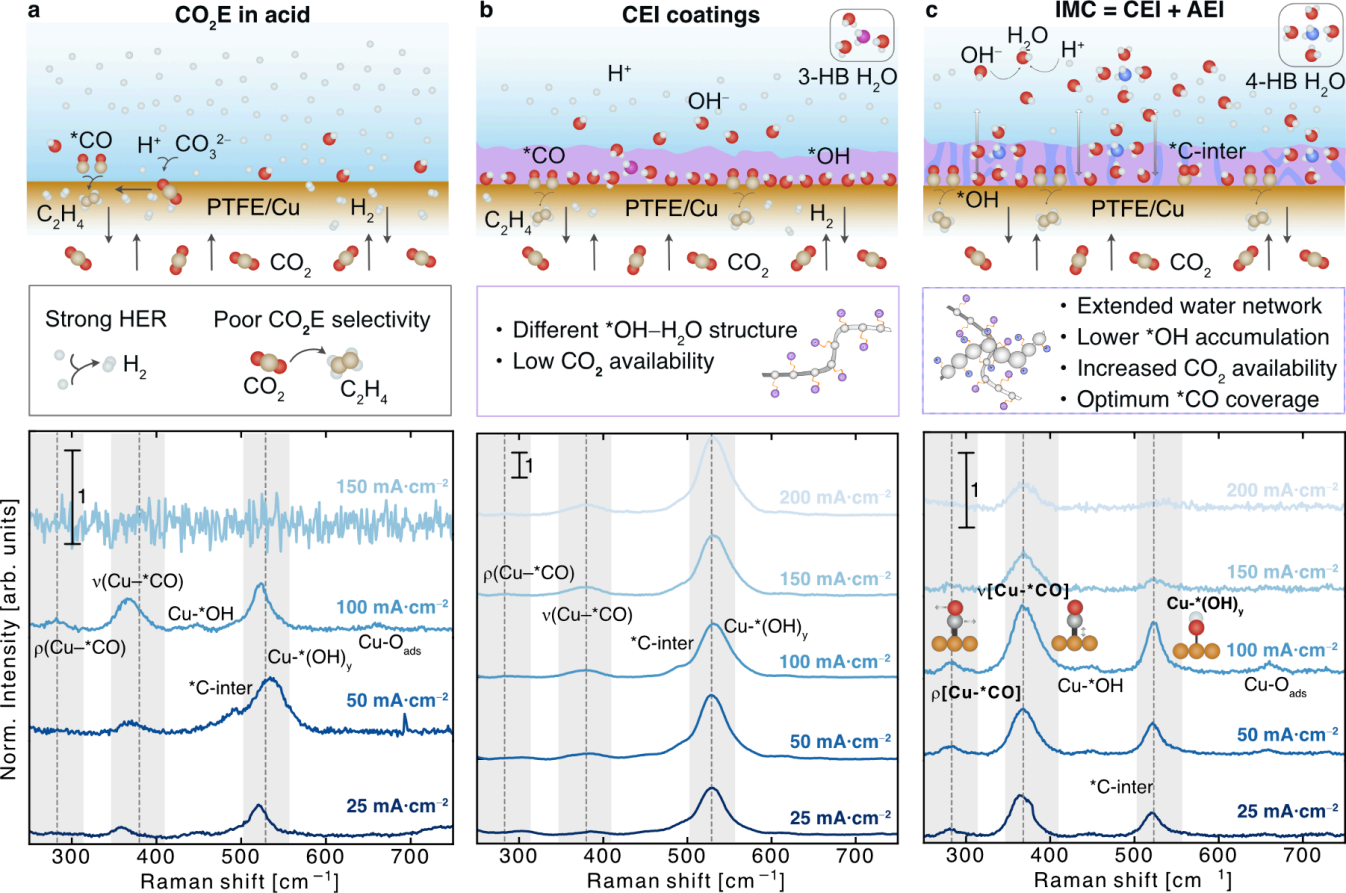
Ion management channels (IMCs) modulating the local reaction environment to enhance C_2**+**_ product formation and SPCU at high currents. **a.** CO_2_E in acid electrolytes mitigates carbonate formation via recombination with local protons. However, this maximizes the hydrogen evolution competing reaction. **b.** CEI coatings improve the CO_2_E selectivity but lead to excessive accumulation of *OH species at high currents, limiting CO_2_ access to active sites and C–C coupling. **c.** IMC channels promote the efficient transport of excessive adsorbed hydroxyl ions (*OH) away from the catalyst surface by forming hydrogen bonds with the surrounding water molecules. Consequently, the availability of CO_2_ and *CO coverage increases, facilitating CO–CO coupling and enhancing C_2+_ product formation. The lower panels show corresponding *in situ* normalized Raman spectra at increasing current densities (25–200 mA·cm^–2^), highlighting differences in surface species under each condition. The highlighted peaks include the frustrated Cu–*CO rotational mode [ρ­(Cu–*CO)] (295–303 cm^–1^), the Cu–*CO stretching mode [ν­(Cu–*CO)] (382–389 cm^–1^), and the Cu–*­(OH)_*y*_ (525 – 550 cm^–1^). Other visible peaks are Cu–*OH (430–470 cm^–1^), *C-inter (490–520 cm^–1^), and *Cu–O_ads._ (614–624 cm^–1^) species. For complete peak assignment and spectra, see Supplementary Table S5 and Figures S35–S38.

In this context, several strategies have been explored to enhance CO_2_E selectivity in acid.^3,6,8–17^ One salient example is the use of cation exchange ionomers (CEI) based on perfluorinated sulfonic acid ionomers such as Aquivion to increase local cation concentration and interfacial pH, effectively shifting the proton source from hydronium to water and favoring CO_2_E.^6,18^ These efforts, among others, highlight the critical yet dynamic role of the reaction environment in CO_2_E, a factor not yet fully understood.

In this study, we delve into the characterization and further control of the CO_2_E reaction environment in acid media using ionomers over benchmark polycrystalline Cu electrodes, focusing on C_2+_ product generation.

Our initial *in situ* Raman studies reveal a previously underappreciated and counterintuitive limitation in ionomer-coated electrodes for CO_2_E in acidic media:^19^ the persistent accumulation of *OH species at the catalyst surface, which impedes *CO adsorption in neighboring sites and significantly limits C_2+_ product formation as current density increases (Figure 1b).^20,21^ This challenges prior assumptions about CO_2_E in acidic conditions, indicating that excess *OH species can represent a detrimental barrier to efficient C–C coupling.^22^

Based on these insights, we reasoned that methods for managing these anion surface species without compromising the benefits endowed by local cation accumulation are essential. To this end, we designed a strategy that enables the formation of ion management channels (IMCs) at the catalyst interface by distributing cation and anion exchange ionomers (CEI and AEI, respectively) (Figure 1c). IMCs balance the availability and surface coverage of key ionic species in CO_2_E (*OH, *OCHCH_2_, *CO, and K^+^) by promoting the desorption of surface-bound *OH through a restructured interfacial water network. This facilitates the removal of excess *OH and enables its recombination with protons (H^+^) before they reach the catalyst surface.^13^ This approach maintains the CO_2_ activation and increases the availability of *CO adsorption sites (Figure 1). Collectively, these effects boost selectivity for C_2+_ products to 80 ± 4% at a current density of 0.5 A·cm^–2^, together with a high carbon utilization (90%) and operational stability of up to 70 h. This represents a relative 39% increase in the maximum partial current density toward C_2+_ products compared to the CEI sample and a 4-fold improvement compared to bare Cu.

We first focused our efforts on assessing the CO_2_E reaction environment in state-of-the-art PTFE/polycrystalline Cu/CEI electrodes. To establish a benchmark for our study, we reproduced and systematically characterized electrodes incorporating Aquivion, a sulfonated tetrafluoroethylene-based polymer that is a proton-conducting ionomer, as it is known to enhance gas-ion-electron transport and improve selectivity and performance.^23^ Electrochemical performance measurements revealed that the Aquivion-coated Cu electrodes exhibit moderate selectivity for C_2+_ products under acidic conditions (Figure 4), consistent with prior reports (Supplementary Figure S58 and Table S7).^6,8^ Structural and compositional analyses confirmed uniform ionomer coverage and stable adhesion to the electrode surface. However, the observed product distribution, together with *in situ* Raman spectroscopy results, suggested an imbalance in ionic flux, which we hypothesized was due to the accumulation of excess *OH near the catalyst surface (Figure 1b).

**Figure 2:**
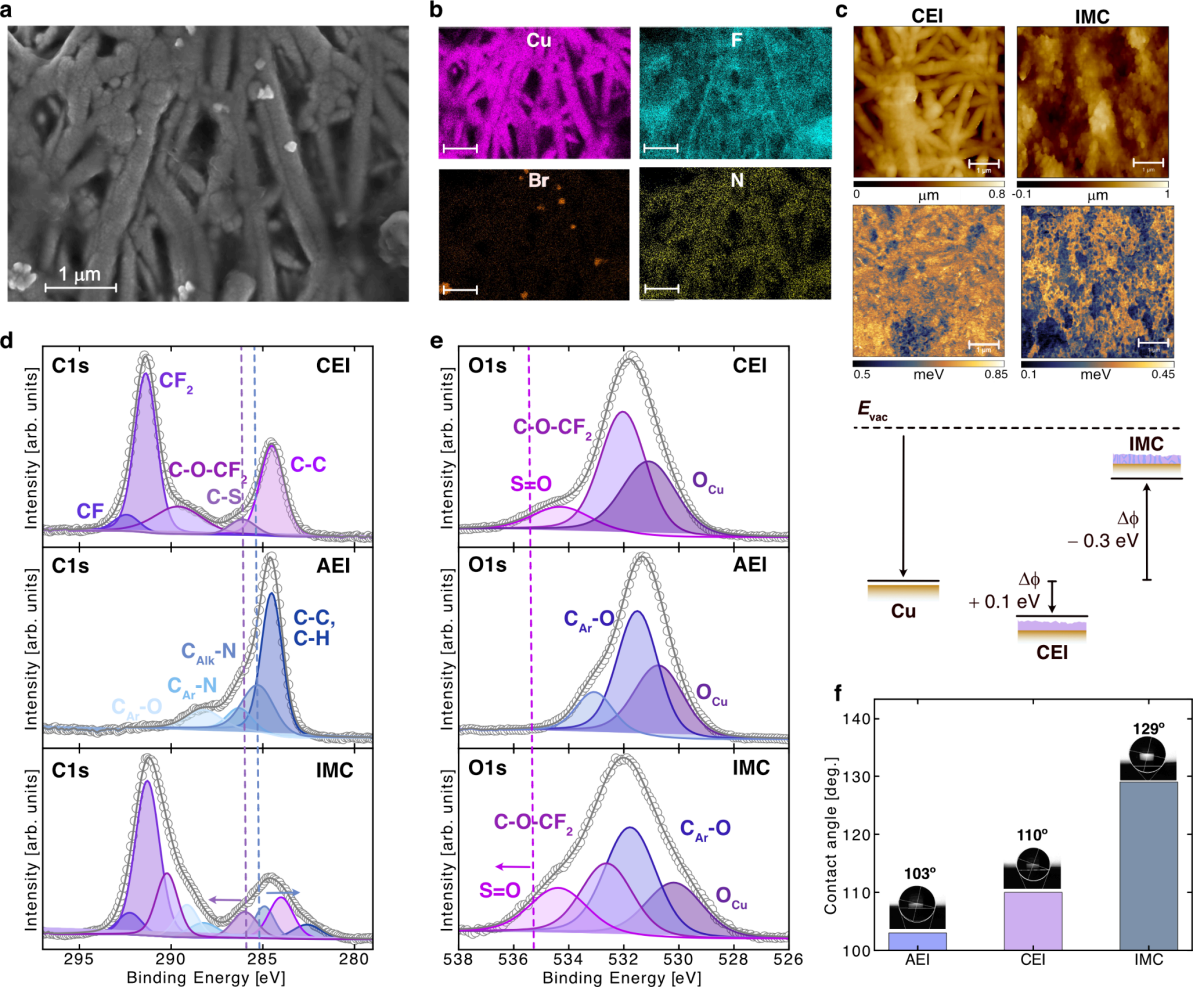
Morphological and compositional analysis of ionomer-coated Cu samples. **a.** SEM of IMC-coated electrodes. **b.** EDS maps show the homogeneous distribution of CEI and AEI in the IMC sample. The fluorine (F) signal corresponds to the CEI, while the nitrogen (N) signal indicates the presence of the AEI. The bromide (Br^–^), originating from the counterion in the quaternary ammonium groups of the AEI, appears localized in isolated pockets, suggesting limited retention within the IMC film. **c.** Work function mapping of the CEI (left) and IMC-coated (right) electrodes by KPFM. The schematic (bottom) summarizes the measured work function shifts (Δϕ) relative to Cu, illustrating how CEI and IMC coatings tune the energy alignment at the electrode–vacuum interface. **d.** C 1s XPS spectra of CEI (top), AEI (middle), and IMC (bottom). C 1s peak in CEI deconvolutes into five peaks: CF, CF_2_, C–O–CF_2_, C–S, and C–C. The C 1s peak in AEI deconvolutes into four peaks: C_Ar_–O, C_Ar_–N, C_Alk_–N, and C–C/C–H. C_Alk_–N shifts to higher binding energy when the ionomers are mixed (IMC). **e.** O 1s XPS spectra of CEI (top), AEI (middle), and IMC (bottom). O 1s peak in CEI deconvolutes into three peaks: S=O, C–O–CF_2_, and O_Cu_. The O 1s peak in AEI deconvolutes into two known peaks: C_Ar_–O and O_Cu_. S=O shifts to a lower binding energy when the ionomers are mixed (IMC). **f.** Contact angles for AEI (blue), CEI (pink), and IMC (gray) show enhanced hydrophobicity for IMC.

**Figure 3:**
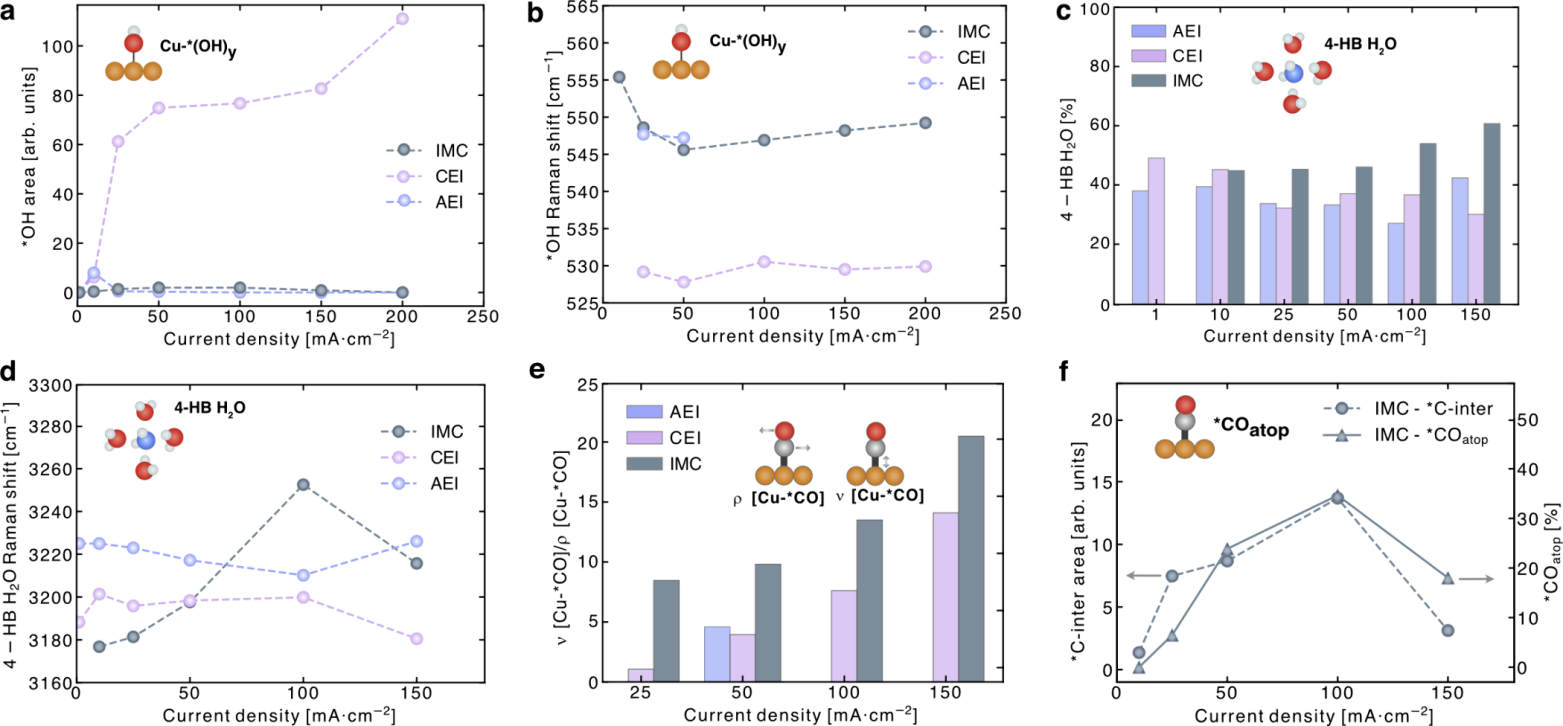
*In situ* electrochemical Raman spectroscopy measurements during CO_2_E. **a.** Comparison of Cu–*OH surface coverage across AEI, CEI, and IMC samples. **b.** Raman shift of the Cu–*OH mode as a function of current density. **c.** The concentration of 4-HB·H_2_O (%) with increasing current density for AEI, CEI, and IMC samples. **d.** Raman shift of the 4-HB·H_2_O mode as a function of the current density **e.** Correlation of Cu–*CO surface coverage with applied potential, represented by the intensity ratio of Cu–*CO stretching to Cu–*CO restricted rotation modes for AEI, CEI, and IMC samples **e.** Cu–*CO coverage potential dependence correlation with the intensity ratio of Cu–*CO stretching over Cu–*CO restricted rotation modes of AEI, CEI, and IMC. **f.** *C-inter and *CO_atop_ modes on the IMC sample at current densities from 10 to 150 mA·cm^–2^. All experiments were conducted in 0.5 M K_2_SO_4_ (pH 2) in a flow cell, with current densities spanning from 1 to 150 mA·cm^–2^.

**Figure 4:**
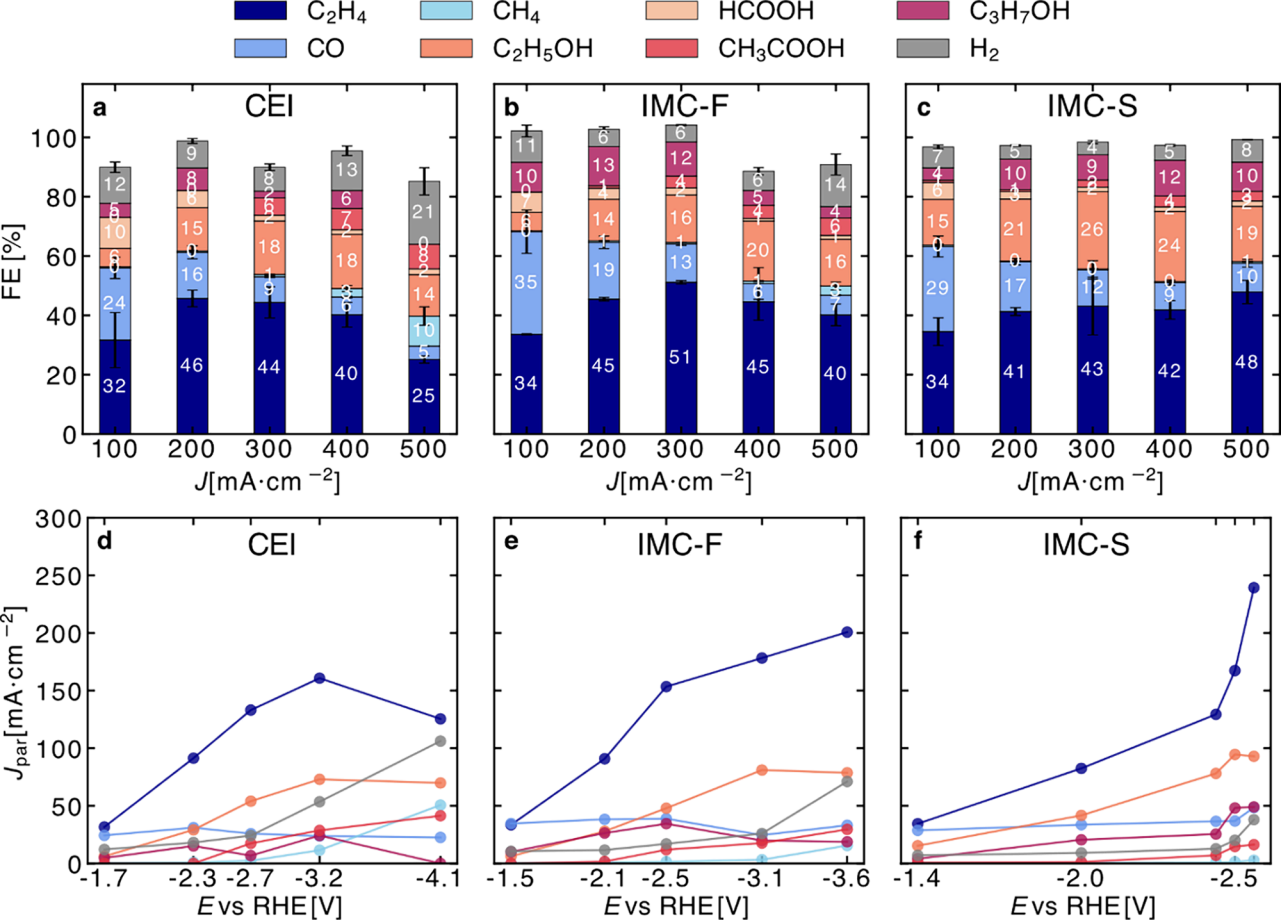
CO_2_E performance across different ionomer configurations: CEI-only, IMC-F (Aquivion + Fumion), and IMC-S (Aquivion + Sustainion XC-2). **a–c.** Faradaic efficiencies (FEs) for major CO_2_ reduction products at total current densities ranging from 100 to 500 mA·cm^–2^. C_2+_ products dominate across all configurations, with the IMC-F and IMC-S structures exhibiting higher selectivity toward C_2_H_4_ and other multicarbon products compared with the CEI-only control. The IMC–Sustainion configuration further enhances C_2+_ selectivity (∼80%) and suppresses HER (<10%) at high current densities (500 mA·cm^–2^). Error bars represent the standard deviation from at least three independent measurements. Missing products correspond to H_2_ not detected in the cathode outlet, due to retention in the catholyte headspace or crossover into the anolyte compartment. **d–f.** Partial current densities (*J*_par_) of each product plotted as a function of measured potential (*E* vs RHE). 85% *iR* compensation was applied based on EIS measurements. The IMC-S system shows a steeper increase in C_2+_ product formation rates with increasing overpotential, indicating improved catalytic utilization and an interfacial environment.

Based on these observations, we posited that the incorporation of a counterionomer with anion exchange properties could address imbalance in ionic flux. Specifically, such a layer would promote the removal of excess surface-bound *OH by enhancing local OH^–^ transport, thereby mitigating anion accumulation at the interface and concurrently limiting proton (H^+^) access to the catalyst surface, which would otherwise favor H_2_ evolution.

To test our hypothesis, we turned our attention to anion exchange ionomers (AEIs) with proven ability to transport hydroxide anions (OH^–^) effectively, thereby facilitating the desorption of adsorbed *OH species. We also selected AEIs with a compatible chemistry with cation exchange ionomer (CEI) dispersions, allowing both components to be processed in similar solvents and enabling the formation of mixed ionomer configurations. In particular, we chose Fumion, a polyaromatic polymer with quaternary ammonium functional groups that conducts electricity by counterion (OH^–^ or CO_3_^2–^/HCO_3_^–^) hopping, as our primary AEI. In addition, we tested Sustainion (imidazolium-based) as an alternative AEI to evaluate how the choice of anion-exchange chemistry influences electrochemical performance. These comparisons highlight that while the IMC concept is general, the specific AEI chemistry can modulate interfacial ion transport and catalytic outcomes.

The AEI and CEI were first characterized independently. Following this, the properties of the optimized mixed ionomer system (IMC) (Supplementary Figures S1–S12) were systematically studied.

Scanning electron microscopy (SEM) (Figure 2a) revealed a uniform distribution of mixed ionomers on the Cu precatalyst surface. Energy dispersive X-ray spectroscopy (EDS) (Figure 2b) confirmed the colocalization of both CEI and AEI ionomers on the IMC sample, which we tracked, monitoring fluorine from the fluorocarbon backbone (CEI) and nitrogen from ammonium groups (AEI), supporting the formation of ion management channels (Supplementary Figures S13–S15). Here, “ion management channels” refers not to fully phase-separated, ordered domains, but to nanoscale spatial structuring within the mixed ionomer layer, where partial segregation and colocalization of CEI and AEI domains create distinct pathways for H^+^ and OH^–^ transport during catalyst layer formation. Control EDS spectra for fluorine in the AEI-only sample and nitrogen in the CEI-only sample are provided in Supplementary Figure S16.

To evaluate the interaction between the ionomers, we measured the attenuated total reflectance-Fourier transformed infrared (ATR-FTIR) spectra of different ionomer implementations. The characteristic peaks associated with the AEI (blue) and with the CEI (pink) exhibited significant shifts in the IMC sample (gray), indicating strong interactions between the two ionomers (Supplementary Figure S21).

Kelvin probe force microscopy (KPFM) reveals a distinct interfacial charge distribution and electronic behavior for IMC- and CEI-coated electrodes (Figure 2c, Supplementary Figures S22 and S23 and Note S1). The CEI layer showed a modest increase in the local work function by ∼0.1 eV relative to bare Cu, consistent with the chemisorption of sulfonate end-groups that orient an interfacial dipole into the surface and withdraw electron density. In contrast, IMC lowers the work function by ∼0.3 eV relative to Cu, which we attribute to the formation of interfacial dipoles from the mixed quaternary ammonium and sulfonate functionalities. We reasoned that this difference in the effective work function, corresponding to an easier extraction of negative charges from Cu, could be a signature of the formation of favorable OH^–^ transport channels; that is, it would also facilitate the extraction of excess adsorbed *OH (Figure 2c, bottom panel).

These findings are consistent with Cu 2p core-level X-ray photoelectron spectroscopy (XPS) (Supplementary Figures S24–S26 and Tables S1–S3), which shows a pronounced shift toward lower binding energies for the IMC-coated electrodes compared to all other samples. This shift reflects a reduced energy requirement to remove electrons from Cu, in agreement with the lower work function observed via KPFM, and supports the presence of more favorable electronic conditions for interfacial charge transfer. To further explore the nature of the electronic interactions between the ionomers, we analyzed high-resolution XPS spectra of C 1s and O 1s core levels (Figure 2d,e). Ionomers over the Cu surface can interact through both electronic and van der Waals forces due to their different charges. In CEI, oxygen atoms in the sulfonate group can transfer their excess electronic charge density to the electron-deficient quartet nitrogen atoms in AEI. The simultaneous blue shift (0.08 eV) and red shift (0.34 eV) observed in the O 1s peak of the sulfonate group and the C 1s peak corresponding to C–N (quartet) suggest the transfer of charge from the sulfonate groups in CEI to the quaternary ammonium groups in AEI within the IMC sample. This insight into the ionomer structure highlights that interactions could be tailored to control the properties of the mixed ionomers and the microenvironment, potentially leading to improved performance.

Contact angle measurements further highlight changes in surface properties, indicating a shift toward a more hydrophobic environment in the IMC sample (Figure 2f, Supplementary Figure S27). We note that the interactions between the AEI and CEI may influence the surface energy and morphology of the ionomer film. Such interactions can modulate the wetting behavior and phase segregation of ion-conducting domains.^24^ While not directly probed in this work, these effects may contribute to the observed electrode coverage and warrant further study. Enhanced hydrophobicity plays a crucial role in increasing local CO_2_ concentration^23,25^ and in the subsequent stabilization of CO_2_E intermediates, such as *CO.^26^ Increased *CO coverage can promote C–C coupling, enhancing pathways that favor multicarbon products. However, our complementary spectroscopic and electrochemical analyses suggest that hydrophobicity alone cannot explain the observed improvements. Instead, the dominant factor appears to be the IMC-enabled regulation of ion transport and the mitigation of *OH poisoning. Post-mortem samples retained a similar hydrophobicity trend (IMC > CEI > AEI), with an overall partially increased hydrophilicity consistent with the formation of Cu oxides, resulting from postelectrolysis air exposure, which can lead to surface oxidation.

Cyclic voltammetry (CV) was performed to evaluate the electrochemical response of Cu-based electrodes under Ar and CO_2_ (Supplementary Figure S28). While bare Cu exhibited minimal differences between the two environments, the presence of ionomer coatings led to distinct shifts in current density. CEI- and IMC-coated electrodes demonstrated the most pronounced separation, indicating enhanced CO_2_ reduction selectivity and suppression of the HER. IMC, in particular, showed the highest current response under CO_2_, suggesting a more favorable interfacial environment for CO_2_ activation and electron transfer.

To assess the preonset local kinetics related to the parasitic HER, we used electrochemical impedance spectroscopy (EIS) under Ar in the absence of CO_2_. Initial analysis using a classical Randles circuit (Supplementary Figure S29) revealed that the IMC-modified electrode exhibited the highest effective double-layer capacitance (*C*_dl_ = 312 μF·cm^–2^) despite having a similar PTFE/Cu base morphology (Supplementary Note S2 and Table S4). This is consistent with the different electrostatic environment revealed by KPFM, contact angle, and XPS measurements. To more accurately capture the physical behavior of the ionomer-coated interfaces, we also applied a mechanistically informed equivalent circuit model (Supplementary Figures S30–S34).^27,28^ This analysis further supports the conclusion that ionomer composition can modulate HER pathways in a way that could potentially favor CO_2_E.

To investigate the potential role of IMC modulating interfacial adsorbates during CO_2_E, we performed *in situ* SERS measurements (see Methods) up to 0.2 A·cm^–2^. These revealed prominent peaks corresponding to the frustrated Cu–*CO rotational mode [ρ­(Cu–*CO)] (295–303 cm^–1^), the Cu–*CO stretching mode [ν­(Cu–*CO)] (382–389 cm^–1^), *CO (1900–2100 cm^–1^), *C-inter (490–520 cm^–1^), *CO_3_^2–^ (∼1062 cm^–1^), and *OH adsorbed species (525–550 cm^–1^) (Supplementary Figure S26, Table S5).^29–32^

Examining the Cu–*­(OH)_*y*_ region (525–550 cm^–1^) as a function of current density for CEI, AEI, and IMC samples provided key insights (Figure 3a). In CEI, *OH accumulation increases sharply with increasing current density, which we hypothesized may limit adjacent available sites for *CO adsorption and C–C coupling. Conversely, the IMC sample exhibits a lower *OH coverage at higher current densities, which could potentially create a more favorable environment for CO adsorption and subsequent C–C coupling.

Additionally, the *OH peak in CEI red-shifts relative to AEI and IMC, indicating weaker interactions with the surface despite higher *OH accumulation (Figure 3b). This trend is corroborated by the similar carbonate peak (∼1,063 cm^–1^) areas and shifts observed for CEI and IMC (Supplementary Figure S45), as well as the absence of a Cu–*OH peak at 450 cm^–1^ in CEI, typically associated with *OH bound to neighboring Cu atoms (Supplementary Figure S39).^31^

Adsorbed *OH species in IMC interfaces may form hydrogen bonds (HB) with surrounding interfacial water molecules, as evidenced by the greater presence of 4-HB·H_2_O (∼3,200 cm^–1^) with increasing current density (Figure 3c and Supplementary Figure S49). This is further supported by the 4-HB·H_2_O peak being blue-shifted in IMC compared to CEI (Figure 3d and Supplementary Figures S47–S49), indicating that water molecules are positioned further from the surface, enabling the formation of more 4-HB interactions. As a result, more active sites in IMC are freed for *CO adsorption, facilitating C–C coupling and enhancing the production of C_2+_ products during CO_2_ electrolysis. Furthermore, this structured water network supports hydroxyl-proton recombination away from the catalyst surface, reducing proton availability for competing HER.^33^

We also observe a higher [ρ­(Cu–CO)]/[ν­(Cu–CO)] ratio for IMC, which increases with the current density (Figure 3e). This ratio is often associated with enhanced C_2+_ selectivity across various catalysts.^20,21^ Multiple *CO vibrational bands between 1900 and 2100 cm^–1^ at high current densities (Supplementary Figure S46) correspond to different vibrational modes of *CO adsorbed on Cu: *CO_bridge_ (∼2,000 cm^–1^) and *CO_atop_ (∼2,060 −2,100 cm^–1^). With increasing current density, the *CO_atop_ adsorption mode becomes more pronounced relative to the *CO_bridge_ (Supplementary Figure S46).^34^ The increased population of weakly bound *CO_atop_ correlates with the rise in the ρ­(Cu–CO)/ν­(Cu–CO) ratio (Figure 3f), while the concurrent suppression of Cu–*OH signals suggests reduced site blocking and a redistribution of surface sites that favors the *CO_atop_ mode. *CO_atop_ may undergo C–C coupling with another *CO_atop_, forming a Raman-active *C-intermediate, which drives C_2_H_4_ (or C_2_H_5_OH) formation.^35^ The multiple *CO vibrational bands observed between 1,900 and 2,100 cm^–1^ at lower currents (≤25 mA·cm^–2^) likely reflect distinct adsorption environments on the Cu surface. These include *CO bound to low-coordinate or defect sites, variations in surface oxidation states (Cu^0^/Cu^+^), and possible enhancement effects under laser excitation. Such spectral complexity is consistent with dynamic surface restructuring and local heterogeneity under electrochemical conditions.^36^

Notably, the adsorbed *C-inter peak (490–525 cm^–1^) is blue-shifted in the IMC sample compared to that in AEI, reflecting stronger interactions with the Cu surface. This enhanced interaction promotes C_2+_ selectivity over HER. Moreover, the *C-inter peak in IMC further blue-shifts with increasing current density, indicating greater stabilization of the intermediate at higher currents (Figure 3f).

Overall, these findings show that the altered environment reshapes the intermediate landscape, potentially enhancing the C–C coupling efficiency.

To assess the catalytic performance of the different ionomers during CO_2_E, IMC electrodes were implemented and measured in a flow-cell reactor (see Methods), where both CO_2_ gas and 0.5 M K_2_SO_4_ (adjusted pH = 2 with conc. H_2_SO_4_) catholyte were continuously flowed into the cell. Figure 4 shows the Faradaic efficiencies (FEs) and partial current densities as a function of potential for CO_2_E on CEI and IMC-coated electrodes (IMC-F: Aquivion + Fumion; IMC-S: Aquivion + Sustainion). Data for bare Cu and AEI-only electrodes are provided in Supplementary Figures S50–S55. Both IMC configurations exhibit a consistent increase in C_2+_ product selectivity compared to CEI, with the enhancement particularly pronounced for C_2_H_4_. At 0.5 A·cm^–2^, the FE for C_2_H_4_ reaches 48% for IMC-S and 40% for IMC-F, compared to 25% for CEI. This trend aligns with *in situ* SERS, suggesting that the presence of a high surface coverage of hydroxyl (*OH) groups in the CEI sample at increasing currents may limit C–C coupling. To further validate the robustness of this strategy, we also tested IMC electrodes in more strongly acidic conditions (pH ∼ 1), where the enhancement in C_2+_ selectivity was maintained (FE toward C_2_H_4_ of 18% at 0.5 A·cm^–2^ for IMC vs 7% for CEI, Supplementary Figure S56), demonstrating that the beneficial effect of IMCs persists even under harsher acidic environments. In addition, we performed CO electroreduction experiments (COR) as a control (Supplementary Figure S57), where carbonate formation does not occur, and observed that IMCs still improved C_2+_ selectivity compared with CEI, further confirming that the benefits of IMCs extend beyond carbonate suppression. Overall, these results indicate that IMCs enhance performance not merely by mitigating carbonate but by fostering a more favorable interfacial ionic environment, facilitating *OH desorption, balancing hydration, and regulating ion transport, which collectively sustain higher *CO coverage and more efficient C–C coupling.

We revisited our initial goal of maximizing carbon utilization in order to minimize product separation costs and evaluated single-pass carbon utilization (SPCU) for our IMC sample. The SPCU is determined by the fraction of the input CO_2_ supply converted to CO_2_E products. We achieved an SPCU of 86.5% at 0.5 sccm for IMC-F at 300 mA·cm^–2^, with 76% of the utilized carbon directed toward C_2+_ products (Figure 5a). For IMC-S, the SPCU reached ∼90% at 1 sccm and 500 mA·cm^–2^, with 86% contributing to C_2+_ products (Figure 5c). Furthermore, through the implementation of an alternating “on” and “off” operating regime (see Methods),^37^ the IMC-F electrode sustained continuous operation for over 60 h (30 h of “on” time) at 0.3 A·cm^–2^ in a strongly acidic environment, maintaining a stable C_2_H_4_ FE of 40% (Figure 5b). Similarly, the IMC-S electrode demonstrated over 70 h of stable operation (35 h of “on” time) under the same conditions, also retaining a C_2_H_4_ FE of ∼40% (Figure 5d).

**Figure 5:**
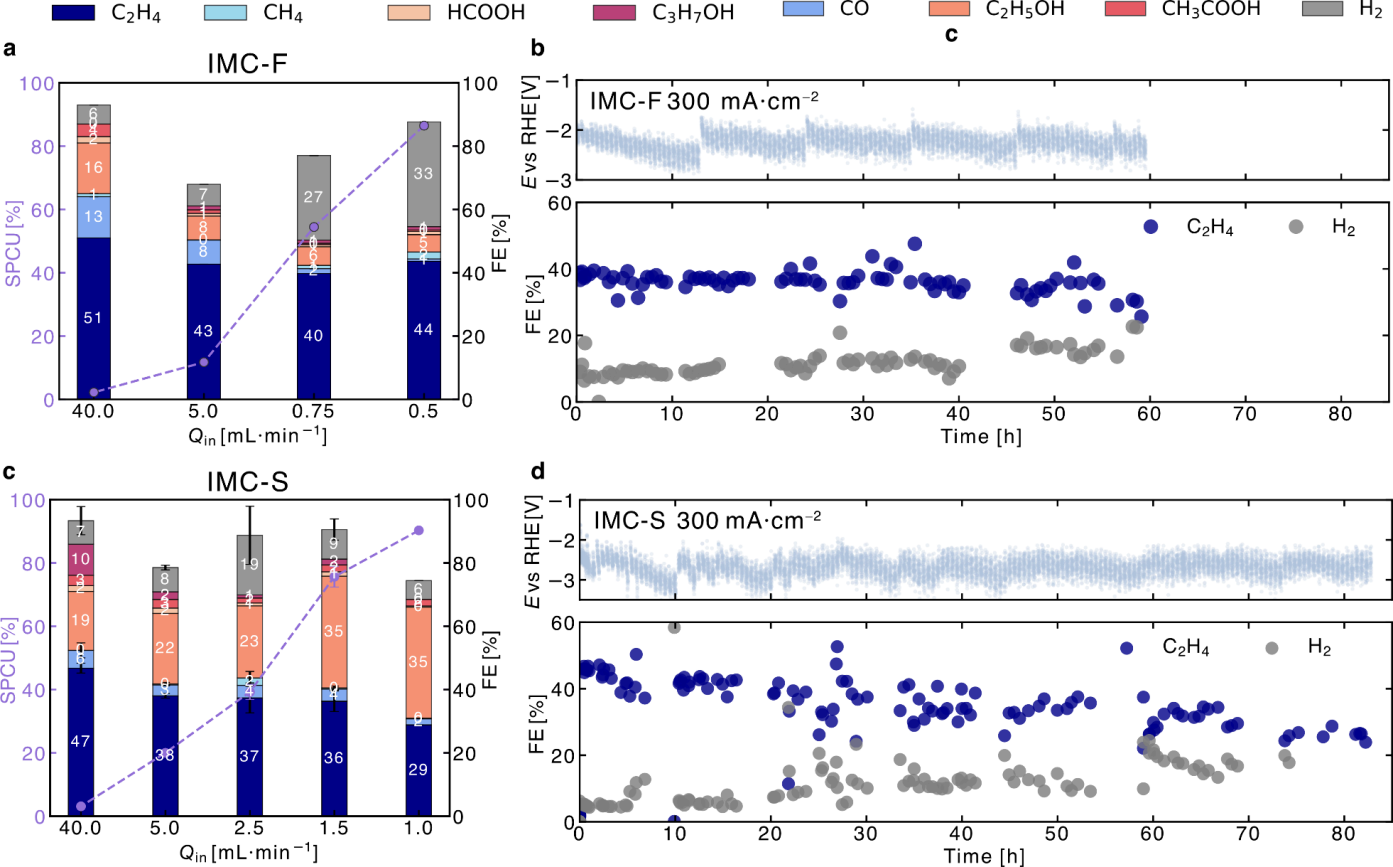
Comparison of CO_2_E performance using IMC-coated Cu electrodes with different AEIs: **a, b** IMC-F (Fumion) and **c,** **d** IMC-S (Sustainion). Single-pass carbon utilization (SPCU, left axis) and Faradaic efficiencies (right axis) toward gaseous and liquid products as a function of decreasing inlet CO_2_ flow rates (*Q*_in_) for **a** IMC-F and **c** IMC-S under a constant current density of 300 mA·cm^–2^ and 500 mA·cm^–2^, respectively. Missing products correspond to H_2_ not detected in the cathode outlet, due to retention in the catholyte headspace or crossover into the anolyte compartment. Chronopotentiometric stability tests at 300 mA·cm^–2^ showing recorded potential vs RHE (top panel, 85% *iR* compensation was applied) and FEs for C_2_H_4_ (dark blue) and H_2_ (gray) (bottom panel) over 80 h of total operational time (40 h of active operation (“on”-time electrolysis)). Results highlight that both IMC-F and IMC-S enhance C_2+_ selectivity while maintaining stable operation, demonstrating that the IMC strategy is effective across different AEI chemistries.

This work showcases the ability to adjust the activity and selectivity of CO_2_E in acid by modulating the chemical microenvironment in close proximity to the surface of a Cu catalyst through the utilization of distributed anion and cation exchange ionomers. The interaction between CEI and AEI leads to the formation of ion management channels that promote transport of alkali cation (K^+^) to the catalyst interface while simultaneously facilitating the desorption and removal of excess surface-bound hydroxyl intermediates (*OH). These *OH species, generated during CO_2_ reduction, are partially desorbed from the catalyst surface when in excess, aided by hydrogen bonding with the restructured interfacial water network. Once desorbed, they exist as solvated OH^–^ and are transported away from the interface through the anion-exchange ionomer (AEI) phase. This dual-ionomer configuration also intercepts protons before they reach the catalyst surface, thereby reducing their availability for the competing HER. While we do not exclude the possibility that near-surface OH^–^ contributes to stabilizing CO,^38^ we attribute the dominant performance enhancement to the IMC architecture, which spatially regulates interfacial ion populations by facilitating excess *OH removal and restructuring interfacial hydration. Catalyst surface reconstruction during CO_2_E is well established and can significantly influence activity and selectivity.^39^ While the central focus of this work is on interfacial ion transport, our postelectrolysis SEM/EDS analysis (Supplementary Figures S17–S19) confirms that the ionomer environment also modulates reconstruction dynamics. All samples displayed moderate surface restructuring, primarily Cu redeposition into oxygen-bearing Cu–O_*x*_ deposits. AEI-only coatings suffered a severe loss of homogeneity, correlating with their poor C_2+_ selectivity, whereas CEI- and IMC-coated electrodes retained their ionomer films and exhibited only moderate reconstruction. Notably, IMC films showed a more pronounced nanoporous texture than CEI after electrolysis, suggesting nanoscale morphological stabilization (Supplementary Figure S20). Elemental mapping further revealed residual K^+^ colocalized with the ionomer domains, consistent with partial uptake by sulfonate groups or entrapment within the polymer network. This retention of K^+^, together with reduced Cu restructuring, may contribute to the enhanced stability and C_2+_ productivity observed with IMC electrodes, in line with recent findings on ionomer-modulated reconstruction.^40^

Operating at an industrially relevant current density of 0.5 A·cm^–2^, we achieve a C_2+_ FE of 80 ± 4% in an acidic environment (pH 2). Additionally, loss of CO_2_ is minimized, and the maximum SPCU exceeds 90% (86% of C_2+_ products), showing a stable performance over 70 h of total operational time, offering valuable insights into ionomer-assisted microenvironment engineering for CO_2_ electrolysis in acid. These findings highlight the potential of IMCs as versatile platforms, where further optimization of CEI–AEI combinations and ratios could unlock even higher C_2+_ selectivity and stability in acidic CO_2_E.

The observed interactions between ionomers and the catalyst surface highlight a promising strategy that could be extended to leverage more sophisticated catalysts tailored to specific intermediates such as bimetallic CuPd or alloyed systems with an intrinsic selectivity for C_2+_ products. Additionally, the principles demonstrated in this study offer guidance for designing catalysts with tailored microenvironments that leverage synergistic effects between ionomer management and surface properties. By systematically integrating ionomer engineering with catalyst surface modifications, this approach could open new avenues for scaling up CO_2_ electrolysis technologies to achieve both higher efficiency and selectivity under industrially relevant conditions.

## Supplementary Material



## Notes

Experimental procedures, surface and morphological characterizations (SEM-EDS, FT-IR, KPFM, XPS, contact angle), voltammetric characterization, EIS data analysis, complete *in situ* SERS data analysis, and electrochemical data sets for catalyst–ionomer interactions (PDF)

## References

[1] Belsa B., Xia L., García de Arquer F. P. (2024). CO_2_ Electrolysis Technologies:
Bridging the Gap toward Scale-up and Commercialization. ACS Energy Lett..

[2] Belsa B., Xia L., Golovanova V., Polesso B., Pinilla-Sánchez A., San Martín L., Ye J., Dinh C. T., García
de Arquer F. P. (2024). Materials Challenges on the Path to Gigatonne CO_2_ Electrolysis. Nat. Rev. Mat.

[3] Li H., Li H., Wei P., Wang Y., Zang Y., Gao D., Wang G., Bao X. (2023). Tailoring Acidic Microenvironments
for Carbon-Efficient CO_2_ Electrolysis over a Ni–N–C
Catalyst in a Membrane Electrode Assembly Electrolyzer. Energy Environ. Sci..

[4] Wakerley D., Lamaison S., Wicks J., Clemens A., Feaster J., Corral D., Jaffer S. A., Sarkar A., Fontecave M., Duoss E. B., Baker S., Sargent E. H., Jaramillo T. F., Hahn C. (2022). Gas Diffusion Electrodes,
Reactor Designs and Key Metrics of Low-Temperature
CO_2_ Electrolysers. Nat. Energy.

[5] Ge L., Rabiee H., Li M., Subramanian S., Zheng Y., Lee J. H., Burdyny T., Wang H. (2022). Electrochemical
CO_2_ Reduction in Membrane-Electrode Assemblies. Chem..

[6] Huang J. E., Li F., Ozden A., Rasouli A. S., de Arquer F. P. G., Liu S., Zhang S., Luo M., Wang X., Lum Y., Xu Y., Bertens K., Miao R. K., Dinh C. T., Sinton D., Sargent E. H. (2021). CO_2_ Electrolysis to Multicarbon
Products in Strong Acid. Science.

[7] Ozden A., García de Arquer F. P., Huang J. E., Wicks J., Sisler J., Miao R. K., O’Brien C. P., Lee G., Wang X., Ip A. H., Sargent E. H., Sinton D. (2022). Carbon-Efficient
Carbon Dioxide Electrolysers. Nat. Sustain.

[8] Zhao Y., Hao L., Ozden A., Liu S., Miao R. K., Ou P., Alkayyali T., Zhang S., Ning J., Liang Y., Xu Y., Fan M., Chen Y., Huang J. E., Xie K., Zhang J., O’Brien C. P., Li F., Sargent E. H., Sinton D. (2023). Conversion
of CO_2_ to Multicarbon Products
in Strong Acid by Controlling the Catalyst Microenvironment. Nat. Synth.

[9] Fan M., Huang J. E., Miao R. K., Mao Y., Ou P., Li F., Li X. Y., Cao Y., Zhang Z., Zhang J., Yan Y., Ozden A., Ni W., Wang Y., Zhao Y., Chen Z., Khatir B., O’Brien C. P., Xu Y., Xiao Y. C., Waterhouse G. I. N., Golovin K., Wang Z., Sargent E. H., Sinton D. (2023). Cationic-Group-Functionalized Electrocatalysts
Enable Stable Acidic CO_2_ Electrolysis. Nat. Catal..

[10] Zeng M., Fang W., Cen Y., Zhang X., Hu Y., Xia B. Y. (2024). Reaction Environment Regulation for Elctrocatalytic
CO_2_ Reduction in Acids. Angew. Chem.
Int. Ed.

[11] Wu W., Xu L., Lu Q., Sun J., Xu Z., Song C., Yu J. C., Wang Y., Wu W., Xu L., Lu Q., Sun J., Xu Z., Song C., Yu J. C., Wang Y. (2025). Addressing the Carbonate Issue: Electrocatalysts for Acidic CO_2_ Reduction Reaction. Adv. Mater..

[12] Li L., Liu Z., Yu X., Zhong M. (2023). Achieving High Single-Pass Carbon
Conversion Efficiencies in Durable CO_2_ Electroreduction
in Strong Acids via Electrode Structure Engineering. Angew. Chem. Int. Ed.

[13] Bondue C. J., Graf M., Goyal A., Koper M. T. M. (2021). Suppression of
Hydrogen Evolution in Acidic Electrolytes by Electrochemical CO_2_ Reduction. J. Am. Chem. Soc..

[14] Gu J., Liu S., Ni W., Ren W., Haussener S., Hu X. (2022). Modulating Electric
Field Distribution by Alkali Cations for CO_2_ Electroreduction
in Strongly Acidic Medium. Nature Catalysis
2022 5:4.

[15] Xie Y., Ou P., Wang X., Xu Z., Li Y. C., Wang Z., Huang J. E., Wicks J., McCallum C., Wang N., Wang Y., Chen T., Lo B. T. W., Sinton D., Yu J. C., Wang Y., Sargent E. H. (2022). High Carbon
Utilization
in CO_2_ Reduction to Multi-Carbon Products in Acidic Media. Nat. Catal.

[16] Ma Z., Yang Z., Lai W., Wang Q., Qiao Y., Tao H., Lian C., Liu M., Ma C., Pan A., Huang H. (2022). CO_2_ Electroreduction to Multicarbon Products in Strongly
Acidic Electrolyte via Synergistically Modulating the Local Microenvironment. Nat. Commun..

[17] Yu X., Xu Y., Li L., Zhang M., Qin W., Che F., Zhong M. (2024). Coverage Enhancement
Accelerates Acidic CO_2_ Electrolysis
at Ampere-Level Current with High Energy and Carbon Efficiencies. Nat. Commun..

[18] Liu X., Koper M. T. M. (2024). Tuning the Interfacial
Reaction Environment for CO_2_ Electroreduction to CO in
Mildly Acidic Media. J. Am. Chem. Soc..

[19] Zhang Q., Ren D., Gao J., Wang Z., Wang J., Pan S., Wang M., Luo J., Zhao Y., Grätzel M., Zhang X. (2023). Regulated CO Adsorption by the Electrode with OH– Repulsive
Property for Enhancing C–C Coupling. Green Chem. Eng..

[20] Gao J., Zhang H., Guo X., Luo J., Zakeeruddin S. M., Ren D., Grätzel M. (2019). Selective C-C Coupling in Carbon
Dioxide Electroreduction via Efficient Spillover of Intermediates
as Supported by Operando Raman Spectroscopy. J. Am. Chem. Soc..

[21] Zhan C., Dattila F., Rettenmaier C., Bergmann A., Kühl S., García-Muelas R., López N., Roldan Cuenya B. (2021). Revealing
the CO Coverage-Driven C-C Coupling Mechanism for Electrochemical
CO_2_ Reduction on Cu_2_O Nanocubesvia OperandoRaman
Spectroscopy. ACS Catal..

[22] Herzog A., Lopez Luna M., Jeon H. S., Rettenmaier C., Grosse P., Bergmann A., Roldan Cuenya B. (2024). Operando Raman
Spectroscopy Uncovers Hydroxide and CO Species Enhance Ethanol Selectivity
during Pulsed CO_2_ Electroreduction. Nat. Commun..

[23] García
de Arquer F. P., Dinh C. T., Ozden A., Wicks J., McCallum C., Kirmani A. R., Nam D. H., Gabardo C., Seifitokaldani A., Wang X., Li Y. C., Li F., Edwards J., Richter L. J., Thorpe S. J., Sinton D., Sargent E. H. (2020). CO_2_ Electrolysis to Multicarbon Products
at Activities Greater than 1 A cm^–2^. Science.

[24] Karan K. (2019). Interesting
Facets of Surface, Interfacial, and Bulk Characteristics of Perfluorinated
Ionomer Films. Langmuir.

[25] Wakerley D., Lamaison S., Ozanam F., Menguy N., Mercier D., Marcus P., Fontecave M., Mougel V. (2019). Bio-Inspired Hydrophobicity
Promotes CO_2_ Reduction on a Cu Surface. Nat. Mater..

[26] Zeng D., Li C., Wang W., Zhang L., Zhang Y., Wang J., Zhang L., Zhou X., Wang W. (2023). Insights into the Hydrophobic
Surface Promoting Electrochemical CO_2_ Reduction to Ethylene. Chemical Engineering Journal.

[27] Schott C., Hofbauer L., Gubanova E., Schneider P., Bandarenka A. S. (2025). Scanning Impedance Microscopy under
Oxygen Reduction
Reaction Conditions. Proof of the Concept. Electrochim.
Acta.

[28] Haimerl F., Kumar S., Heere M., Bandarenka A. S. (2024). Electrochemical
Impedance Spectroscopy of PEM Fuel Cells at Low Hydrogen Partial Pressures:
Efficient Cell Tests for Mass Production. Industrial
Chemistry & Materials.

[29] An H., de Ruiter J., Wu L., Yang S., Meirer F., van der Stam W., Weckhuysen B. M. (2023). Spatiotemporal Mapping of Local Heterogeneities
during Electrochemical Carbon Dioxide Reduction. JACS Au.

[30] Moradzaman M., Mul G. (2021). In Situ Raman Study of Potential-Dependent Surface Adsorbed Carbonate,
CO, OH, and C Species on Cu Electrodes During Electrochemical Reduction
of CO_2_. ChemElectroChem..

[31] Niaura G. (2000). Surface-Enhanced
Raman Spectroscopic Observation of Two Kinds of Adsorbed OH^–^ Ions at Copper Electrode. Electrochim. Acta.

[32] Zhao Y., Zhang X. G., Bodappa N., Yang W. M., Liang Q., Radjenovica P. M., Wang Y. H., Zhang Y. J., Dong J. C., Tian Z. Q., Li J. F. (2022). Elucidating Electrochemical CO_2_ Reduction Reaction Processes on Cu­(Hkl) Single-Crystal Surfaces
by in Situ Raman Spectroscopy. Energy Environ.
Sci..

[33] Wang Y., Zhang J., Zhao J., Wei Y., Chen S., Zhao H., Su Y., Ding S., Xiao C. (2024). Strong Hydrogen-Bonded
Interfacial Water Inhibiting Hydrogen Evolution Kinetics to Promote
Electrochemical CO_2_ Reduction to C_2+_. ACS Catal..

[34] An H., Wu L., Mandemaker L. D. B., Yang S., de Ruiter J., Wijten J. H. J., Janssens J. C. L., Hartman T., van der
Stam W., Weckhuysen B. M. (2021). Sub-Second Time-Resolved Surface-Enhanced
Raman Spectroscopy Reveals Dynamic CO Intermediates during Electrochemical
CO_2_ Reduction on Copper. Angew. Chem.
Int. Ed.

[35] Ren D., Fong J., Yeo B. S. (2018). The Effects of Currents and Potentials
on the Selectivities of Copper toward Carbon Dioxide Electroreduction. Nat. Commun..

[36] Zang J., Ye W., Liu Q., Meng J., Yang W. (2025). Plasmonic-Promoted
Formation of Surface Adsorbed Stochastic CO during Electrochemical
CO_2_ and CO Reduction on Cu at Extreme Low Overpotentials. J. Am. Chem. Soc..

[37] Nguyen T. N., Chen Z., Zeraati A. S., Shiran H. S., Sadaf S. M., Kibria M. G., Sargent E. H., Dinh C. T. (2022). Catalyst Regeneration
via Chemical Oxidation Enables Long-Term Electrochemical Carbon Dioxide
Reduction. J. Am. Chem. Soc..

[38] Iijima G., Inomata T., Yamaguchi H., Ito M., Masuda H. (2019). Role of a
Hydroxide Layer on Cu Electrodes in Electrochemical CO_2_ Reduction. ACS Catal..

[39] Kwon W., Kim D., Lee Y., Jung J., Nam D. H. (2025). Advancements in
Understanding Catalyst Reconstruction During Electrochemical CO_2_ Reduction. Exploration.

[40] Peerlings M. L. J., Vink-Van Ittersum M. E. T., de Rijk J. W., de Jongh P. E., Ngene P. (2025). Ionomer-Modulated Electrochemical
Interface Leading to Improved Selectivity
and Stability of Cu_2_O-Derived Catalysts for CO_2_ Electroreduction. ACS Catal..

