# Supplementary material for: Carbon Efficient CO2 Interfaces in Acid through Ion Management Channels
Source: ACS Energy Lett. 2025 Dec 15;11(1):498–507. doi: 10.1021/acsenergylett.5c02981 (PMC12797322; doi:10.1021/acsenergylett.5c02981)
Supplement: Supplementary file 1 [file nz5c02981_si_001.pdf]

Carbon Efficient CO_2 Interfaces in Acid through Ion Management Channels

Blanca Belsa [a], Anku Guha [a], Barbara Polesso [a], Ranit Ram [a], Viktoria Golovanova [a], Marinos Dimitropoulos [a], Sunil Kadam [a], Prathama Haldar [a], Aliaksandr S. Bandarenka [b,c], and F. Pelayo García de Arquer [a]*

[a] ICFO - Institut de Ciències Fotòniques, The Barcelona Institute of Science and Technology, Castelldefels (Barcelona) 08860, Spain

[b] Physik-Department ECS, Technische Universität München, James-Franck-Str. 1, Garching D-85748, Germany

[c] Catalysis Research Center TUM, Ernst-Otto-Fischer-Straße 1, Garching bei München 85748, Germany

*Email: pelayo.garciadearquer@icfo.eu

Supporting information

This PDF file includes:

Supplementary Figures 1 – 58

Supplementary Tables 1 – 7

Supplementary References 1 – 34

METHODS AND MATERIALS

Preparation of Cu:ionomer electrodes

Cu electrodes, with 300 nm nominal thickness, were prepared by sputtering pure Cu target onto a polytetrafluoroethylene (PTFE) gas diffusion layer (GDL) with 450 nm pore size. The reference 16 cm^2 PTFE/Cu electrodes were modified by spray-coating an ionomer layer from a solution of 20 µL Fumion (FM-FAA-3-SOLUT-10 anion exchange polymer solution in N-methyl-2-pyrrolidone (NMP) solvent solution purchased from QuinTech) (AEI electrode); 40 µl Aquivion (Aquivion D79-25BS perfluorinated resin solution, product #802565-25 ml purchased from Sigma Aldrich) (CEI electrode); 20 µL Fumion + 40 µL Aquivion (IMC-F electrode) in 16 mL methanol (99.9%, Scharlau) and sonicated at least 30 min before spray coating. The final ionomer loadings were 1.25 $\mu L \cdot cm^{-2}$ (AEI), 2.5 $\mu L \cdot cm^{-2}$ (CEI), and 3.75 $\mu L \cdot cm^{-2}$ (IMC-F). For the IMC with Sustainion (IMC-S); 40 µL Sustainion XC-2 (Sustainion® XC-2 Alkaline Ionomer 5% in ethanol from Dioxide Materials) + 40 µL Aquivion were mixed in 16 mL methanol.

Materials characterization

Ionomer Compositions for Characterization Samples: All characterization measurements (SERS, XPS, KPFM, EDS) were conducted on sputtered Cu on PTFE (300 nm) modified with IMC layers using 2.5 $\mu L \cdot cm^{-2}$ Aquivion (CEI) and 1.25 $\mu L \cdot cm^{-2}$ Fumion (AEI), corresponding to a CEI:AEI volume ratio of 3:2. This composition was chosen based on electrochemical performance optimization and used consistently across all interfacial analyses. CEI(AEI)-modified electrodes were prepared on sputtered Cu on PTFE (300 nm) using 2.5 (1.25) $\mu L \cdot cm^{-2}$ Aquivion (Fumion).

Contact angle measurements: Water contact angles were measured using the sessile drop method on a DataPhysics OCA 15EC video-based contact angle goniometer equipped with automated image analysis software. A droplet of ultrapure Milli-Q water (3–5 µL) was carefully deposited on the sample surface using a microliter syringe. After 15 seconds of equilibration to allow droplet stabilization and surface wetting, the contact angle was recorded from the captured side-view image using the instrument's integrated software. Measurements were performed under ambient laboratory conditions (22-25 °C, ~50-60% relative humidity). All samples were measured after drying under ambient conditions, without further post-treatment.

Attenuated total reflectance-Fourier transform infrared (ATR-FTIR): spectra were obtained from the electrodes by using Agilent Cary 630 FTIR. Measurements were performed in transmittance mode over a wavenumber range of 4000 – 650 cm^{-1}, with a spectral resolution of 2 cm^{-1}. Each spectrum was averaged over 128 scans to improve the signal-to-noise ratio, with 128 background scans recorded immediately before measurement to minimize interference from ambient water vapor and CO_2.

Scanning electron microscopy (SEM): SEM images were acquired using a Zeiss Gemini FE-SEM apparatus at 5 kV accelerating voltage and different magnifications.

Energy-dispersive X-ray spectroscopy (EDS): EDS maps were acquired at 5 kV using a windowless EDS detector (Ultim Extreme Oxford) at 5 kV and 30 μm aperture at different magnifications. Each map was acquired for 100 frames at process time = 6.

X-ray photoelectron spectroscopy (XPS): was carried out using SPECS PHOIBOS 150. XPS data analysis and fitting were carried out using CasaXPS software. The bindihong energy of all peaks was corrected with respect to the C1s peak (284.5 eV).

Atomic force microscopy (AFM): AFM measurements were performed on a Park Systems NX20 under ambient conditions in non-contact mode. For electrical measurements, the specimens were mounted on a steel disk and were electrically grounded using conductive silver paste (RS Components).

Topography and nanomechanical data: AC160TS probes (R=7nm, k=26 $N{\cdot}m^{-1}$, f=300kHz) were used for the topography and nanomechanical maps. The deflection sensitivity was calibrated on a sapphire standard.

Kelvin Probe Force Microscopy (KPFM): Pt-Ir coated PPP-EFM probes (R = 25 nm, k = 2.8 $N{\cdot}m^{-1}$, f = 75 kHz) were employed for the surface potential mapping. The scan rates were maintained low at 0.2-0.3 Hz which enabled the feedback control to keep a stable scan regime, reliably tracking larger topographic features. In order to get quantitative results, the work function of the tip was calibrated on a HOPG standard via the following equation:

$$\Phi_s = \Phi_t - eV_{CPD},$$

where, Φ_S is the work function of the sample, Φ_t is the work function of the tip, e is the electron charge and V_{CPD} is the contact potential difference.

In situ Raman measurements

In situ Raman spectra were recorded with a Renishaw Raman spectrometer equipped an L63x (Leica) magnification water-immersion objective covered with a double layer of PTFE film (25 μm thick) with 0.9 numerical aperture with using a 785 nm excitation laser with 0.1% of laser power and 1800 l/mm grating. Spectral data were collected within the range of 100 – 2,250 cm^{-1} over 30 acquisitions with each acquisition having an exposure time of 2 seconds in a custom-made PTFE flow cell. The *in situ* flow cell featured a liquid electrolyte reservoir (K_2SO_4 0.5M adjusted with concentrated H_2SO_4 until pH = 2) in which the immersion objective was dipped. A GDE separated the electrolyte reservoir from the gas channel, which continuously supplied CO_2 gas to the catalyst at a flow rate of 40 sccm. The area of the electrode in this configuration was 0.5 cm^2 and the required currents were applied using a single-channel Autolab 204 potentiostat/galvanostat. The counter electrode, a Pt wire, and the reference electrode, Ag/AgCl (3M KCl), were dipped in the electrolyte reservoir ~1 cm from the cathode. All spectra were single-point normalized to background signal. Baseline correction was carried out using asymmetrically reweighted penalized least squares smoothing[1] and bands were deconvoluted into one-to-three Gaussian peaks depending on goodness of fit using the lmfit (Non-linear Least-Squares Minimization and Curve -Fitting) package for Python.[2]

For the water structure analysis, *in situ* Raman spectroscopy was conducted using a custom-made single-compartment Raman cell. The same water immersion objective (L63x), covered with a 25 μm thick PTFE film, was used with a 633 nm laser equipped with a 1200 l/mm grating. All measurements were taken at 5% laser power, with a 2-second laser exposure and 30 accumulations. The data were deconvoluted into 3 or 2 Gaussian peaks using OriginPro 2022 software.

Electrochemical reduction of CO_2

<u>*Flow-cell components*</u>*:* The flow cell consists of three compartments: anolyte, catholyte and gas chambers. The anolyte chamber (12 mm x 12 mm; 15 mm depth) houses the counter electrode (Pt mesh). In the catholyte chamber (12 mm x 12 mm; 15 mm depth), a square through hole accommodates the insertion of the Ag/AgCl (3M KCl) reference electrode, positioned such that the frit of the reference electrode lies in the center of the chamber. Separating the anolyte and catholyte chambers is a cation exchange membrane (Nafion 117). The gas chamber (12 mm x 12 mm; 15 mm depth) is designated for supplying the reactant gas. The cathode separates the gas chamber from the catholyte chamber. The catalyst side of the cathode faces into the catholyte chamber, while the PTFE gas diffusion layer faces the gas chamber. To ensure proper

sealing, silicone gaskets featuring a 1 cm^2 window are interposed between each layer. Each chamber is equipped with inlet and outlet connections (1/8" OD; 1/16" ID) for the flow either electrolyte or gas.

Flow-cell assembly and operation: The cathode and commercially available Pt mesh anodes were installed in their designated chambers using copper tape leads to establish electrical contact and Kapton tape to ensure sealing. Starting from the anolyte chamber, the entire assembly was sealed with uniform compression achieved by tightening four evenly spaced bolts. In this setup, the cathode functions as the working electrode. The electrode potentials were adjusted to the reversible hydrogen electrode (RHE) scale using the following expression: $E_{\mathrm{RHE}} = E_{\mathrm{Ag/AgCl}} + 0.223\ \mathrm{V} + 0.059\ \times pH$; where E_{RHE} is the potential of the RHE, $E_{\mathrm{Ag/AgCl}}$ is the applied potential, and pH is the acidity of the catholyte. The electrochemical tests were performed using a potentiostat (Autolab PGSTAT302N), a custom-made flow cell with a fixed 1 cm^2 electrode geometric area, a digital mass flow controller (Sierra, SmartTrack 100) setting a CO_2 flow of 40 mL·min^{-1}, a current booster (Metrohm Autolab, 10 A), and two peristaltic pumps with silicone tubing. For all measurements, the anolyte was H_2SO_4 0.5 M solution, and the catholyte K_2SO_4 0.5 M adjusted to pH = 2 using H_2SO_4. The electrolyte flow rate was set to 30 mL·min^{-1} and the volumes used for circulation were 25 mL. The ohmic resistance and charge transfer resistance were measured through electrochemical impedance spectroscopy (EIS), and the data points were obtained between 0.1 Hz and 100 kHz.

Stability measurements: Stability measurements were performed using the abovementioned flow-cell. The sample underwent 1 hour of continuous electrolysis at 0.3 A·cm^{-2} and then objected to an electrolysis sequence of alternating – /15 min " “on”/15 min “off”/ – segments. Argon is introduced in the catholyte headspace with a flow rate of 40 sccm to account for the products left out there. The catholyte (anolyte) was changed by a new 0.5 M K_2SO_4/H_2SO_4, pH 2 (0.5 M H_2SO_4) every 3-4 (10) hours of operation, approximately. Moreover, salt accumulated at the cathode was washed with MiliQ water and N_2 during the “off” time.

CO_2E gaseous product analysis: Gas products resulting from reduction reactions were collected in 1 mL volumes using gas-tight syringes (Hamilton chromatography syringes) at least three times for each applied current. Subsequently, this volume was injected into a gas chromatograph (PerkinElmer Clarus 590), which was outfitted with a thermal conductivity detector (TCD), a flame ionization detector (FID), and two packed columns (Carboxen-1000). Argon served as the carrier gas in the gas.

The Faradic Efficiencies were determined as:

$$FE\ (\%) = \frac{n \times F \times V_{\mathrm{m}} \times f_{\mathrm{m}}}{J} \times 100$$

where n is the number of electrons for a given product; F is the Faradaic constant; V_{m} is the molar volume; f_{m} is the molar reacting gas flow rate and J is the total current.

<u>CO_2E liquid product analysis</u>: liquid products were analyzed using proton nuclear magnetic resonance spectroscopy (^{1}H-NMR) under water suppression mode. Dimethyl sulfoxide (DMSO) was used as the reference and deuterium oxide (D_2O) as the lock solvent. The flow cell was operated at the desired applied current for at least for 800 s before sample collection. Cathode electrolyte was collected from the flow cell end tubing, sealed and stored in a fridge until ^{1}H-NMR sample preparation. For ^{1}H-NMR sample preparation, 500 μL of stored solutions were mixed with 500 μL of 14.04 mM DMSO solution in D_2O. The FE of liquid was calculated via the following equation:

$$FE\ (\%) = \frac{z \times n \times F}{J \times t} \times 100$$

where z represents the mole number of products, n is the number of electrons for a given product; F is the Faradaic constant; J is the total current and t represents the electrolysis time.
<u>Single-pass carbon utilization</u>: Single-pass carbon utilization (SPCU) was calculated using the following equation:

$$SPCU\ (\%) = \frac{\frac{j}{n \times F} \times V_m}{f_m} \times 100$$

where j represents the partial current density of a specific product; n is the number of electrons for a given product; F is the Faradaic constant; V_{m} is the molar volume and f_{m} is the molar reacting gas flow rate.

Ionomer loading optimization

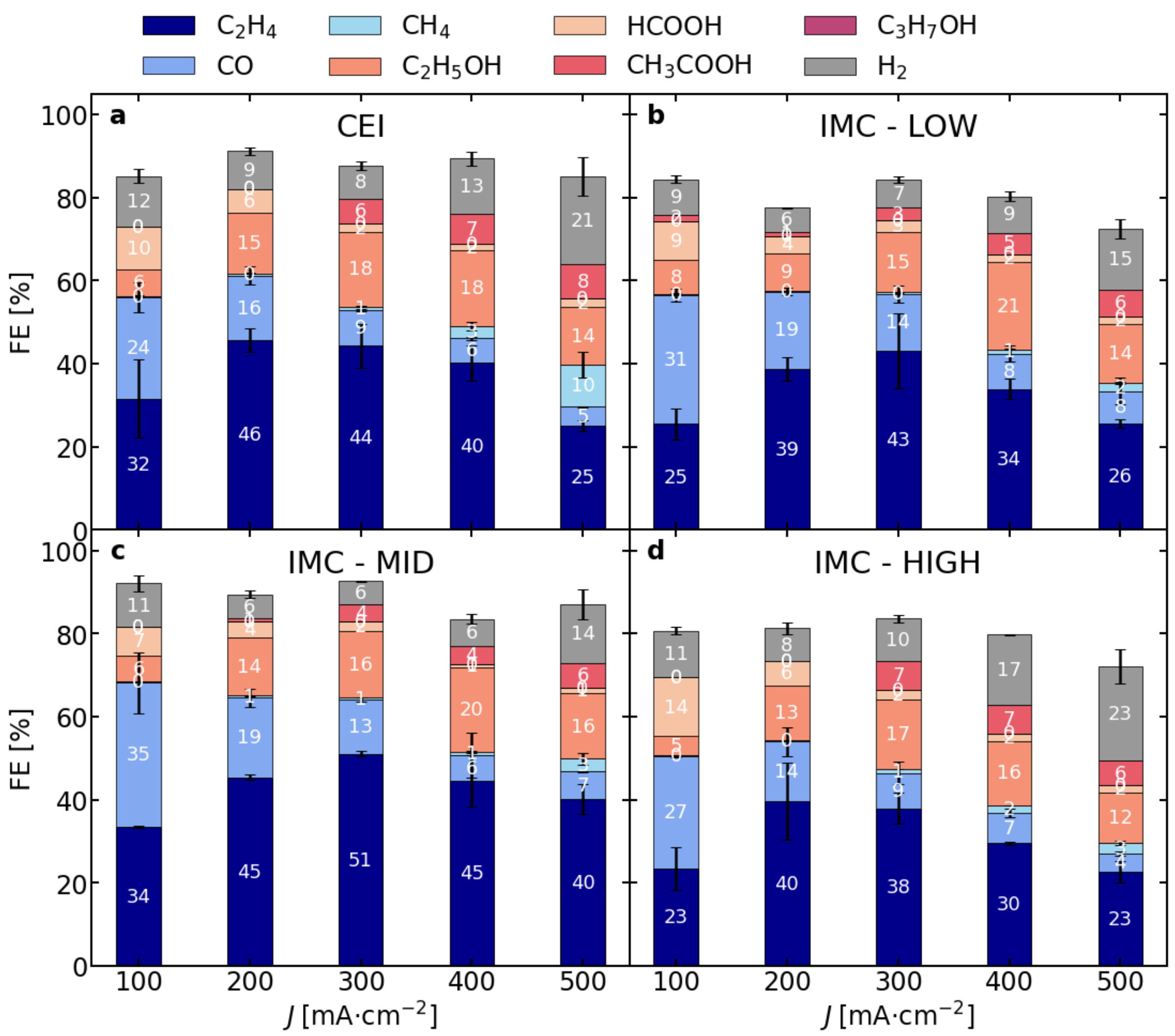

Figure S1. Distributed ionomer loading optimization. Faradaic efficiencies (%FE) of CO_2E for **a**, CEI (Aquivion 2.5 µL·cm^{-2}) **b**, IMC-low (Aquivion 2.5 µL·cm^{-2} + Fumion 0.5 µL·cm^{-2}) **c**, IMC-mid (Aquivion 2.5 µL·cm^{-2} + Fumion 1.25 µL·cm^{-2}) and **d**, IMC-high (Aquivion 2.5 µL·cm^{-2} + Fumion 2.0 µL·cm^{-2}) in the presence of acidic electrolyte (0.5 M K_2SO_4 + H_2SO_4 (pH = 2)). Error bars indicate standard deviation among values from three repeated measurements. Missing products correspond to H_2 not detected in the cathode outlet, due to retention in the catholyte headspace or crossover into the anolyte compartment.

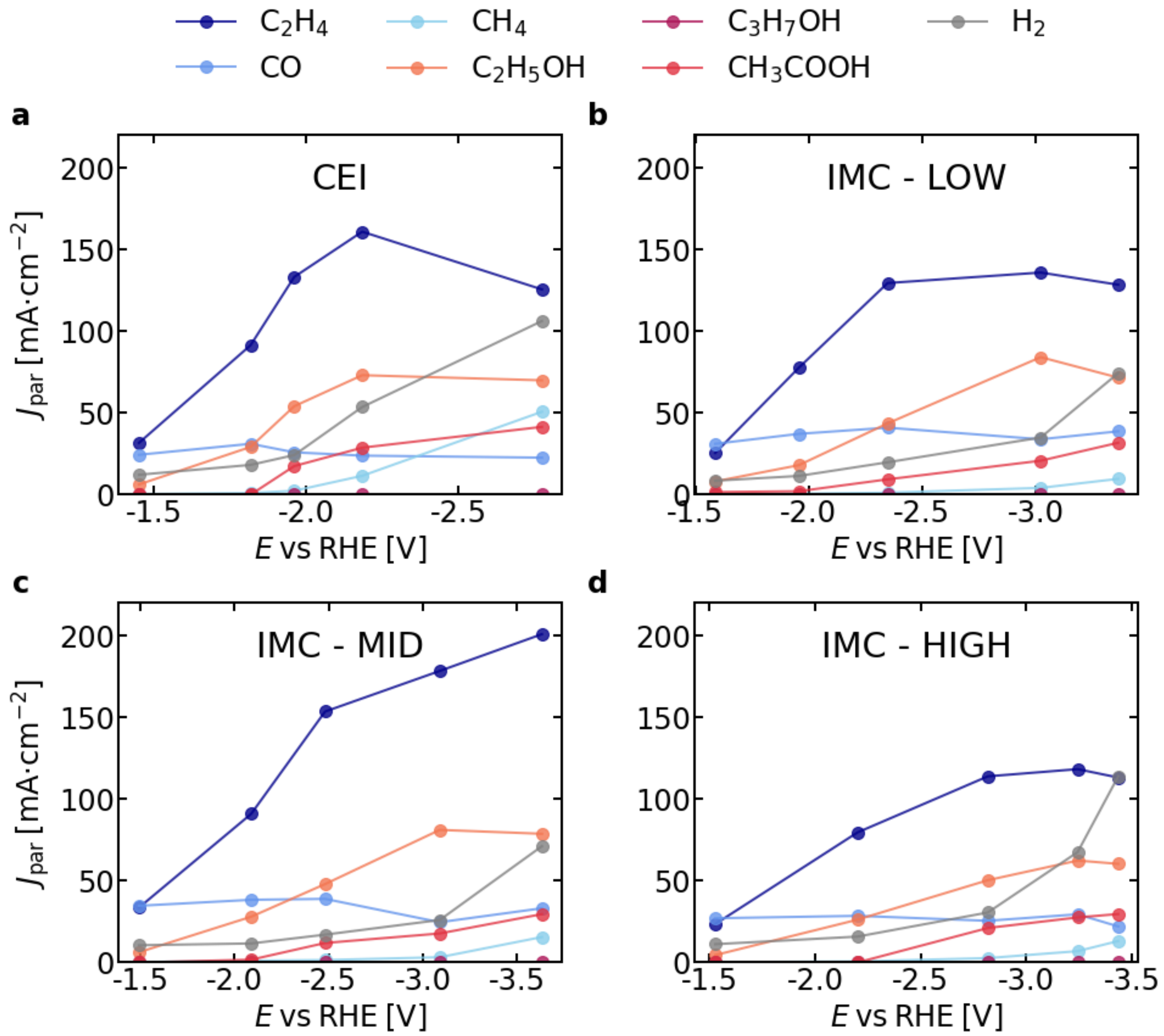

Figure S2. Distributed ionomer loading optimization. Partial current densities of CO_2E using **a**, CEI (Aquivion 2.5 μL·cm^{-2}) **b**, IMC-low (Aquivion 2.5 μL·cm^{-2} + Fumion 0.5 μL·cm^{-2}) **c**, IMC-mid (Aquivion 2.5 μL·cm^{-2} + Fumion 1.25 μL·cm^{-2}) and **d**, IMC-high (Aquivion 2.5 μL·cm^{-2} + Fumion 2.0 μL·cm^{-2}) in the presence of acidic electrolyte (0.5 M K_2SO_4 + H_2SO_4 (pH = 2)). 85% *iR* compensation was applied based on EIS measurements.

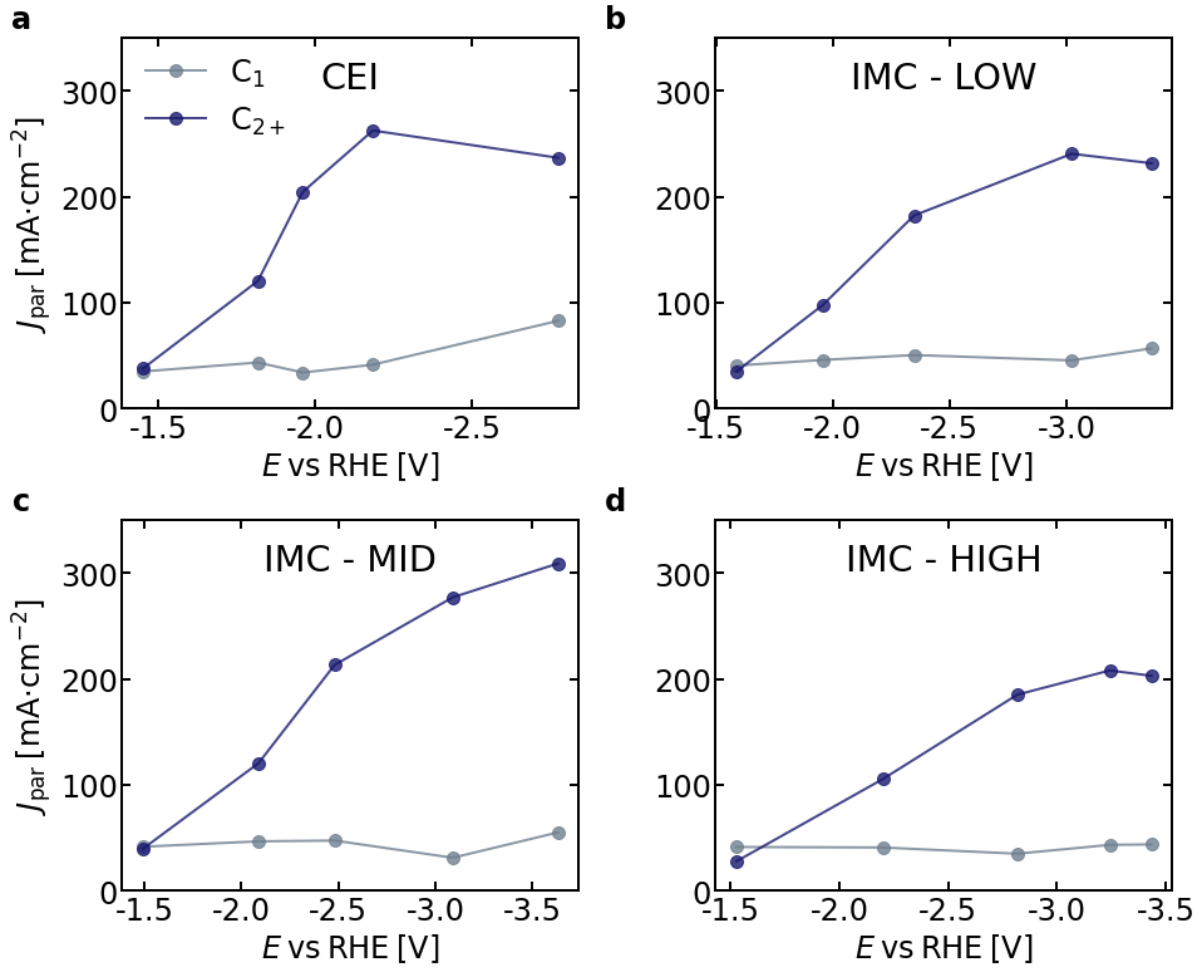

Figure S3. Distributed ionomer loading optimization. C_1 (gray) vs C_{2+} (blue) partial current densities for **a**, CEI (Aquivion 2.5 μL·cm^{-2}) **b**, IMC-low (Aquivion 2.5 μL·cm^{-2} + Fumion 0.5 μL·cm^{-2}) **c**, IMC-mid (Aquivion 2.5 μL·cm^{-2} + Fumion 1.25 μL·cm^{-2}) and **d**, IMC-high (Aquivion 2.5 μL·cm^{-2} + Fumion 2.0 μL·cm^{-2}) in the presence of acidic electrolyte (0.5 M K_2SO_4 + H_2SO_4 (pH = 2)). 85% *iR* compensation was applied based on EIS measurements.

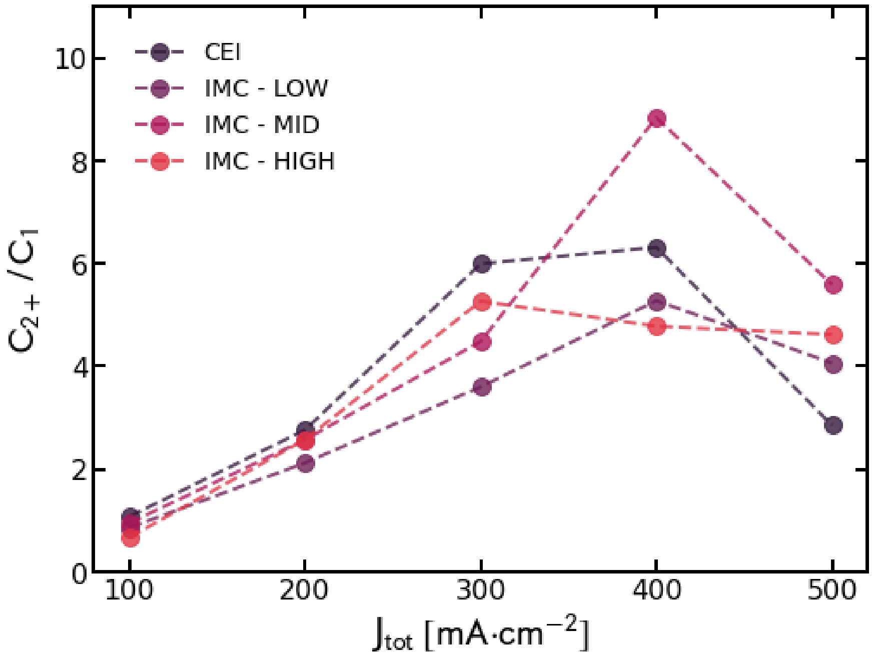

Figure S4. C_{2+}/C_1 ratio for CEI (Aquivion 2.5 μL·cm^{-2}) (dark purple), IMC-low (Aquivion 2.5 μL·cm^{-2} + Fumion 0.5 μL·cm^{-2}) (purple), IMC-mid (Aquivion 2.5 μL·cm^{-2} + Fumion 1.25 μL·cm^{-2}) (violet) and, IMC-high (Aquivion 2.5 μL·cm^{-2} + Fumion 2.0 μL·cm^{-2}) (orange) in the presence of acidic electrolyte (0.5 M K_2SO_4 + H_2SO_4 (pH = 2)).

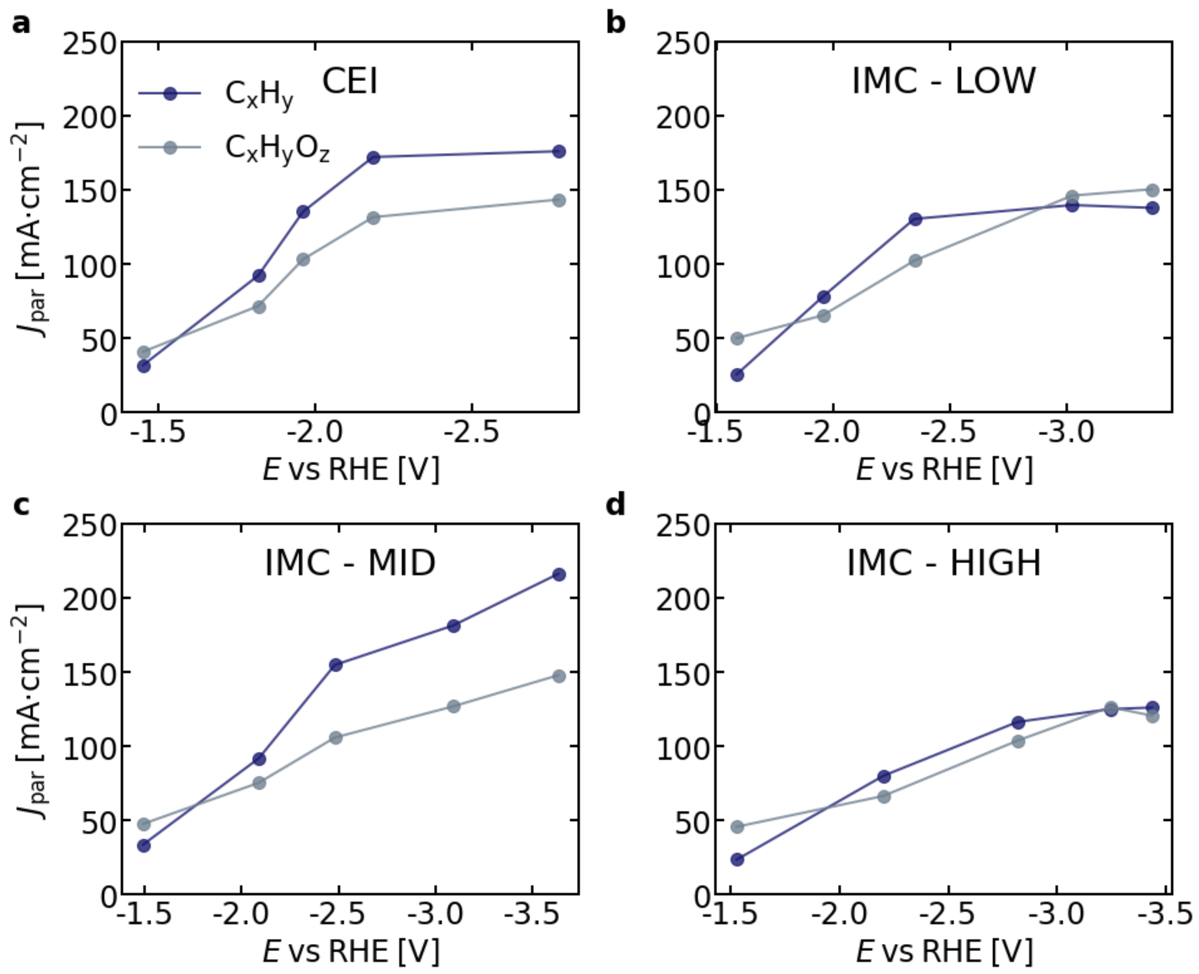

Figure S5. Distributed ionomer loading optimization. Hydrocarbons, C_xH_y (blue) vs oxyhydrocarbons $C_xH_yO_z$ (gray) partial current densities for **a**, CEI (Aquivion 2.5 μL·cm^{-2}) **b**, IMC-low (Aquivion 2.5 μL·cm^{-2} + Fumion 0.5 μL·cm^{-2}) **c**, IMC-mid (Aquivion 2.5 μL·cm^{-2} + Fumion 1.25 μL·cm^{-2}) and **d**, IMC-high (Aquivion 2.5 μL·cm^{-2} + Fumion 2.0 μL·cm^{-2}) in the presence of acidic electrolyte (0.5 M K_2SO_4 + H_2SO_4 (pH = 2)). 85% *iR* compensation was applied based on EIS measurements.

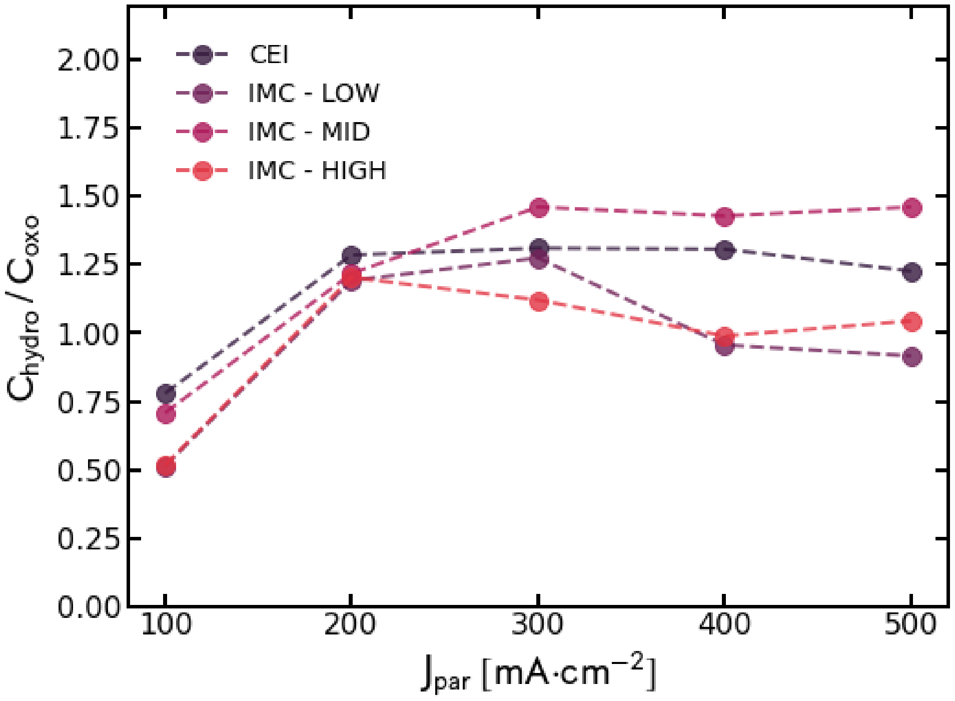

Figure S6. Hydrocarbons, C_xH_y vs oxyhydrocarbons $C_xH_yO_z$ ratio (C_{hydro}/C_{oxo}) for CEI (Aquivion 2.5 µL·cm^{-2}) (dark purple), IMC-low (Aquivion 2.5 µL·cm^{-2} + Fumion 0.5 µL·cm^{-2}) (purple), IMC-mid (Aquivion 2.5 µL·cm^{-2} + Fumion 1.25 µL·cm^{-2}) (violet) and, IMC-high (Aquivion 2.5 µL·cm^{-2} + Fumion 2.0 µL·cm^{-2}) (orange) in the presence of acidic electrolyte (0.5 M K_2SO_4 + H_2SO_4 (pH = 2)).

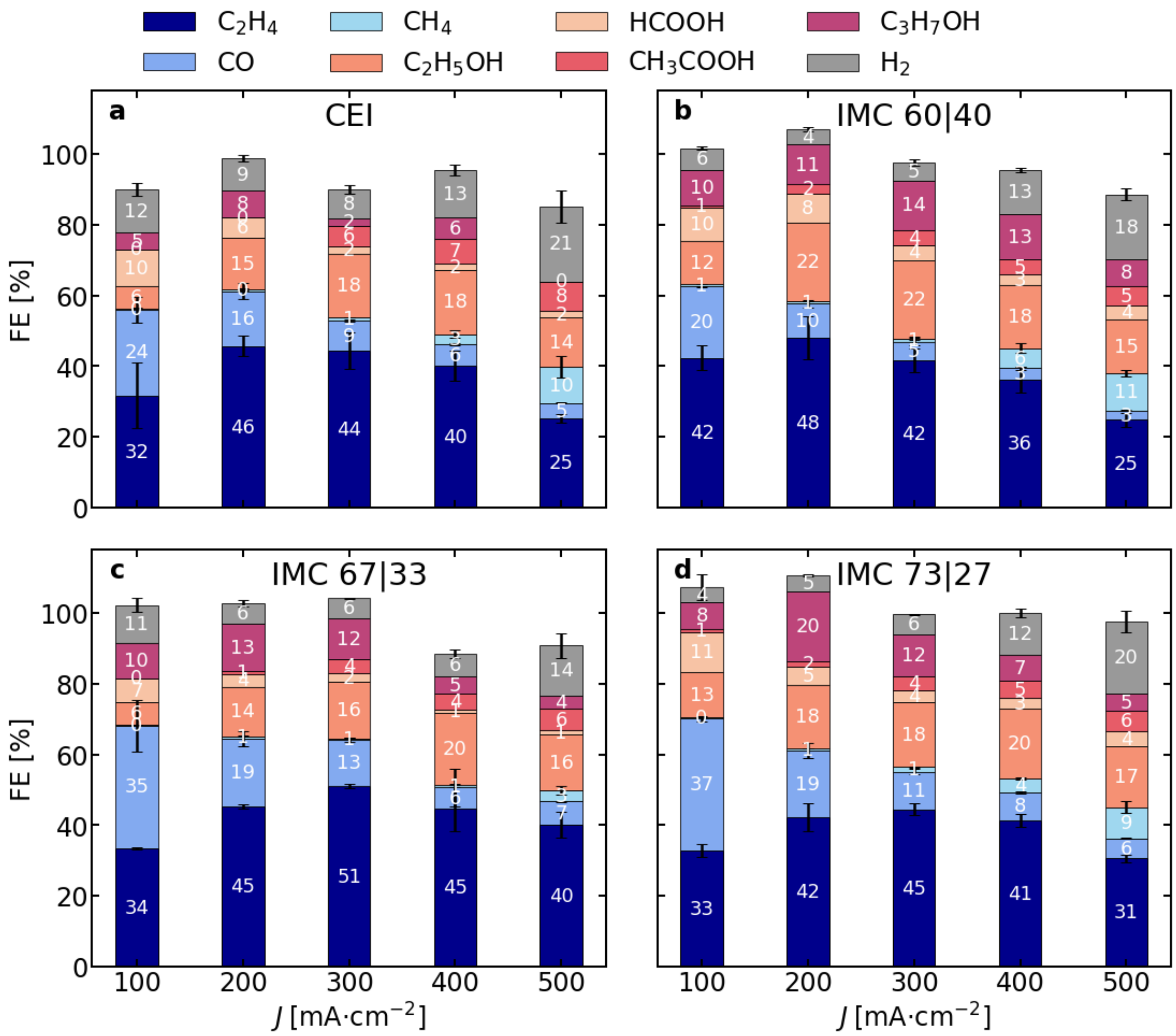

Figure S7. Distributed ionomer loading optimization at fixed total loading (3.75 μL·cm^{-2})**.** Faradaic efficiencies (%FE) of CO_2E for **a**, CEI (Aquivion 2.5 μL·cm^{-2}) **b**, IMC 60|40 (Aquivion 2.25 μL·cm^{-2} + Fumion 1.5 μL·cm^{-2}) **c**, IMC 67|33 (Aquivion 2.5 μL·cm^{-2} + Fumion 1.25 μL·cm^{-2}) and **d**, IMC 73|27 (Aquivion 2.75 μL·cm^{-2} + Fumion 1.0 μL·cm$^{\square 2}$) in the presence of acidic electrolyte (0.5 M K_2SO_4 + H_2SO_4 (pH = 2)). Error bars indicate standard deviation among values from three repeated measurements. Multicarbon product (C_{2+}) selectivity is consistently enhanced in the IMC configurations compared to the CEI-only control. Among the IMC formulations, the 67|33 (v/v) ratio achieves the highest C_2H_4 selectivity and lowest HER across all current densities tested. Error bars represent the standard deviation from at least three independent measurements. Missing products correspond to H_2 not detected in the cathode outlet, due to retention in the catholyte headspace or crossover into the anolyte compartment.

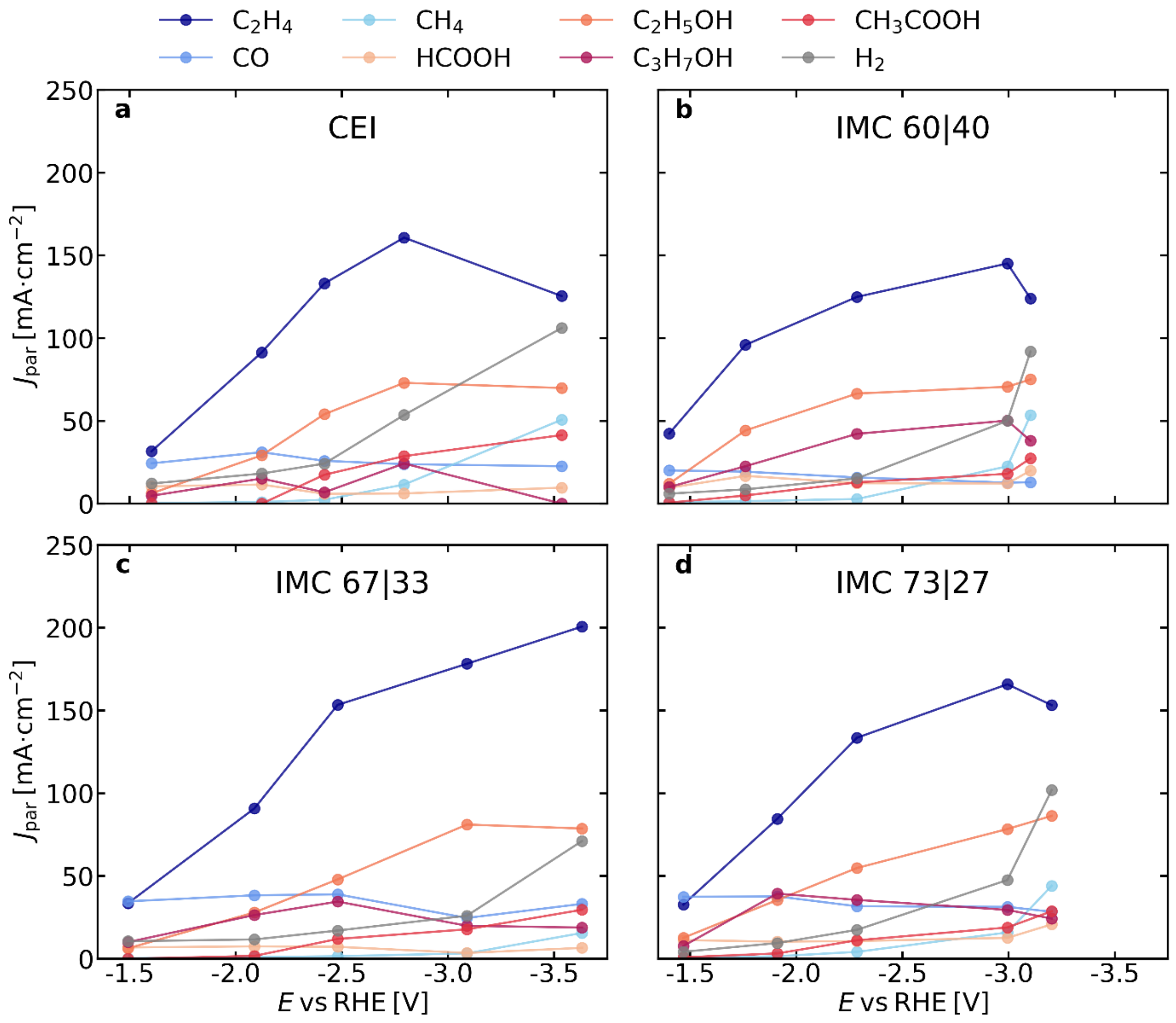

Figure S8. Distributed ionomer loading optimization at fixed total loading (3.75 µL·cm^{-2}). Partial current densities (J_{par}) of CO_2E as a function of measured potential (E vs RHE) for **a**, CEI (Aquivion 2.5 µL·cm^{-2}) **b**, IMC 60|40 (Aquivion 2.25 µL·cm^{-2} + Fumion 1.5 µL·cm^{-2}) **c**, IMC 67|33 (Aquivion 2.5 µL·cm^{-2} + Fumion 1.25 µL·cm^{-2}) and **d**, IMC 73|27 (Aquivion 2.75 µL·cm^{-2} + Fumion 1.0 µL·cm^{-2}) in the presence of acidic electrolyte (0.5 M K_2SO_4 + H_2SO_4 (pH = 2)). 85% *iR* compensation was applied based on EIS measurements. Multicarbon product (C_{2+}) selectivity is consistently enhanced in the IMC configurations compared to the CEI-only control. The IMC 67|33 configuration achieves the highest partial current density for C_2H_4 and lowest for HER. The performance of other IMC variants also exceeds the CEI-only case, confirming the benefits of mixed ionomer structuring.

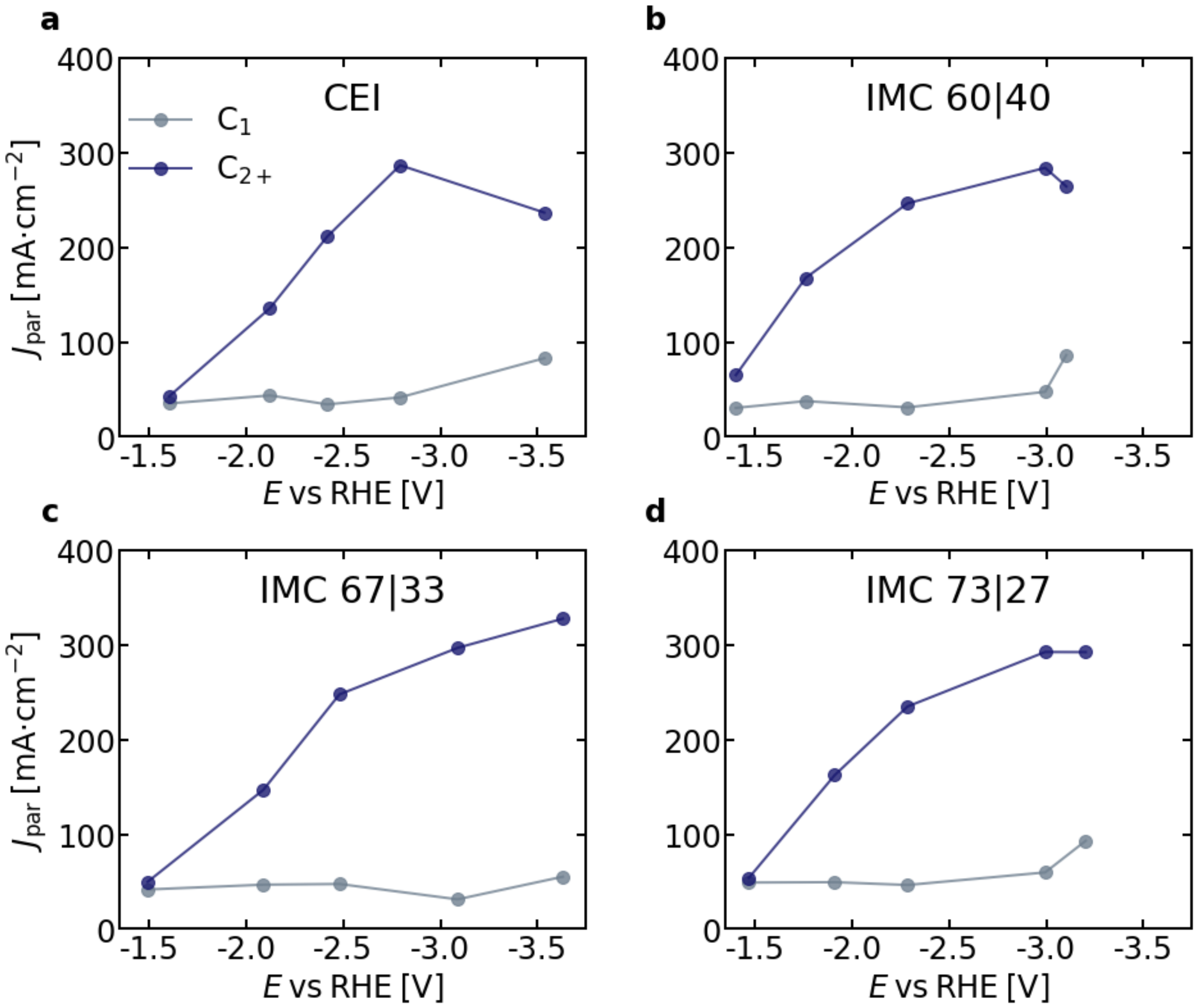

Figure S9. Distributed ionomer loading optimization at fixed total loading (3.75 µL·cm^{-2}). C_1 (gray) vs C_{2+} (blue) partial current densities for **a**, CEI (Aquivion 2.5 µL·cm^{-2}) **b**, IMC 60|40 (Aquivion 2.25 µL·cm^{-2} + Fumion 1.5 µL·cm^{-2}) **c**, IMC 67|33 (Aquivion 2.5 µL·cm^{-2} + Fumion 1.25 µL·cm^{-2}) and **d**, IMC 73|27 (Aquivion 2.75 µL·cm^{-2} + Fumion 1.0 µL·cm^{-2}) in the presence of acidic electrolyte (0.5 M K_2SO_4 + H_2SO_4 (pH = 2)). 85% *iR* compensation was applied based on EIS measurements.

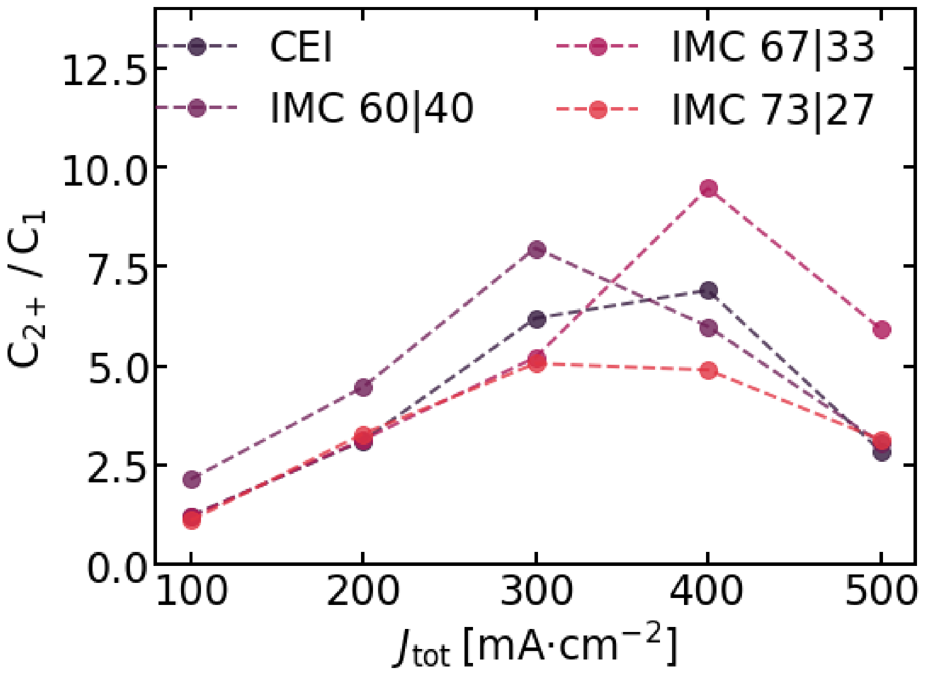

Figure S10. C_{2+}/C_1 ratio for CEI (Aquivion 2.5 µL·cm^{-2}) (dark purple), IMC 67|33 (Aquivion 2.5 µL·cm^{-2} + Fumion 1.25 µL·cm^{-2}) (purple), IMC 60|40 (Aquivion 2.25 µL·cm^{-2} + Fumion 1.5 µL·cm^{-2}) (violet) and, IMC 73|27 (Aquivion 2.75 µL·cm^{-2} + Fumion 1.0 µL·cm^{-2}) (orange) in the presence of acidic electrolyte (0.5 M K_2SO_4 + H_2SO_4 (pH = 2)).

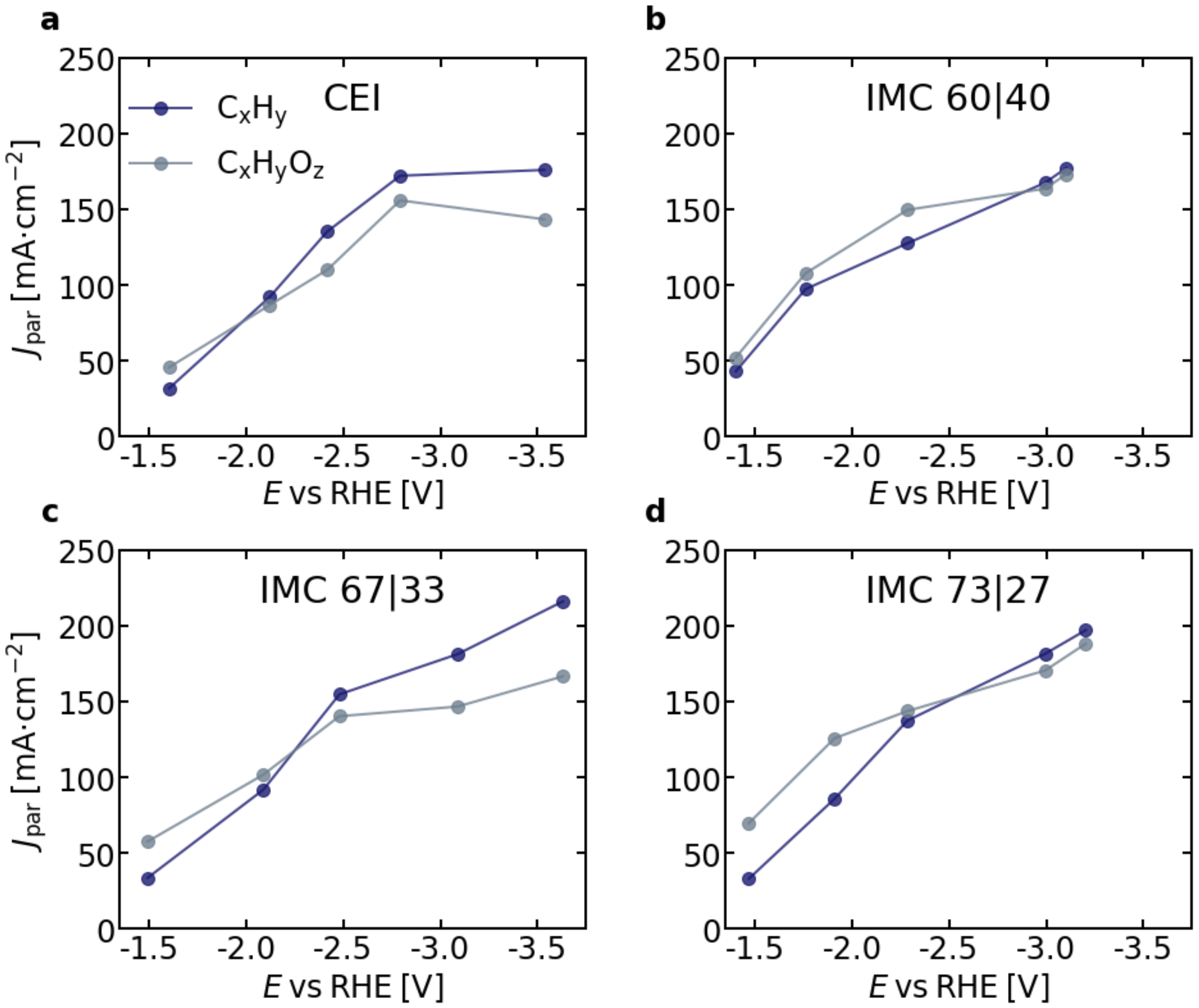

Figure S11. Distributed ionomer loading optimization at fixed total loading (3.75 µL·cm^{-2}). Hydrocarbons, C_xH_y (blue) vs oxyhydrocarbons $C_xH_yO_z$ (gray) partial current densities for **a**, CEI (Aquivion 2.5 µL·cm^{-2}) **b**, IMC 60|40 (Aquivion 2.25 µL·cm^{-2} + Fumion 1.5 µL·cm^{-2}) **c**, IMC 67|33 (Aquivion 2.5 µL·cm^{-2} + Fumion 1.25 µL·cm^{-2}) and **d**, IMC 73|27 (Aquivion 2.75 µL·cm^{-2} + Fumion 1.0 µL·cm^{-2}) in the presence of acidic electrolyte (0.5 M K_2SO_4 + H_2SO_4 (pH = 2)). 85% *iR* compensation was applied based on EIS measurements.

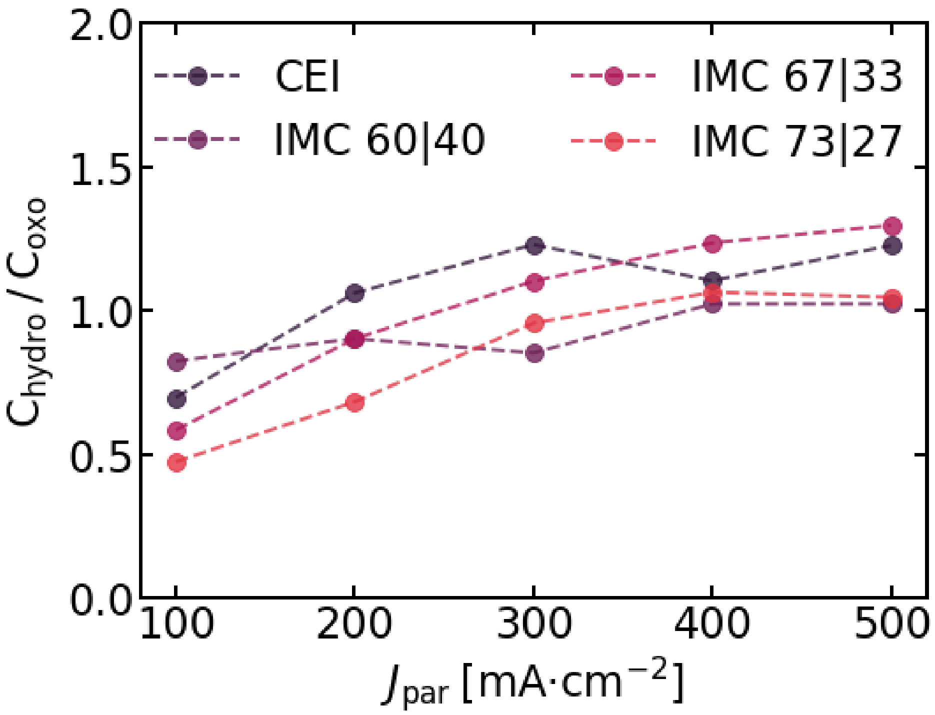

Figure S12. Hydrocarbons, C_xH_y vs oxyhydrocarbons $C_xH_yO_z$ ratio (C_{hydro}/C_{oxo}) for CEI (Aquivion 2.5 µL·cm^{-2}) (dark purple), IMC 67|33 (Aquivion 2.5 µL·cm^{-2} + Fumion 1.25 µL·cm^{-2}) (purple), IMC 60|40 (Aquivion 2.25 µL·cm^{-2} + Fumion 1.5 µL·cm^{-2}) (violet) and, IMC 73|27 (Aquivion 2.75 µL·cm^{-2} + Fumion 1.0 µL·cm^{-2}) (orange) in the presence of acidic electrolyte (0.5 M K_2SO_4 + H_2SO_4 (pH = 2)).

Morphological analysis

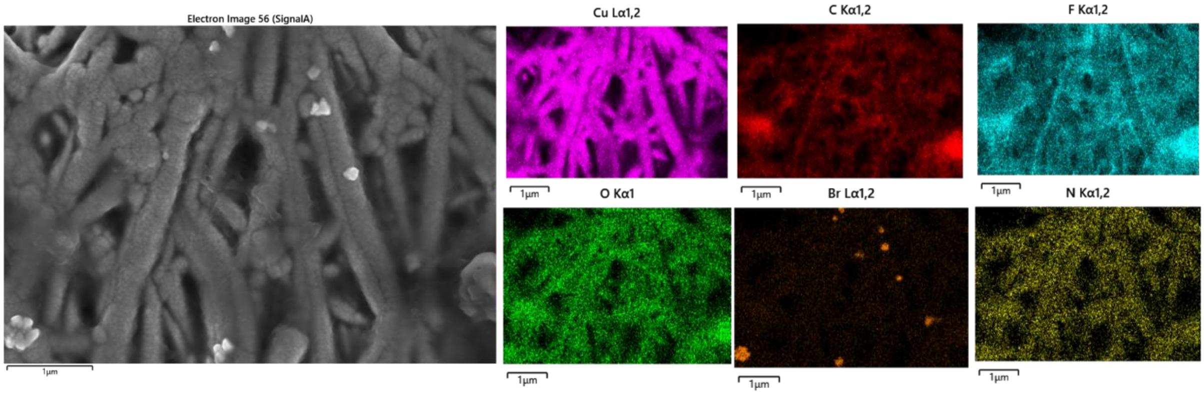

Figure S13. Scanning electron microscopy (SEM) and energy-dispersive X-ray spectroscopy (EDS) elemental mapping of the IMC-coated electrode. The left panel presents a high-resolution SEM image, revealing the intricate nanostructured morphology of the IMC sample. The coloured panels on the right display EDS elemental maps for key elements (Cu, C, F O, Br and N), demonstrating their homogeneous distribution across the structure.

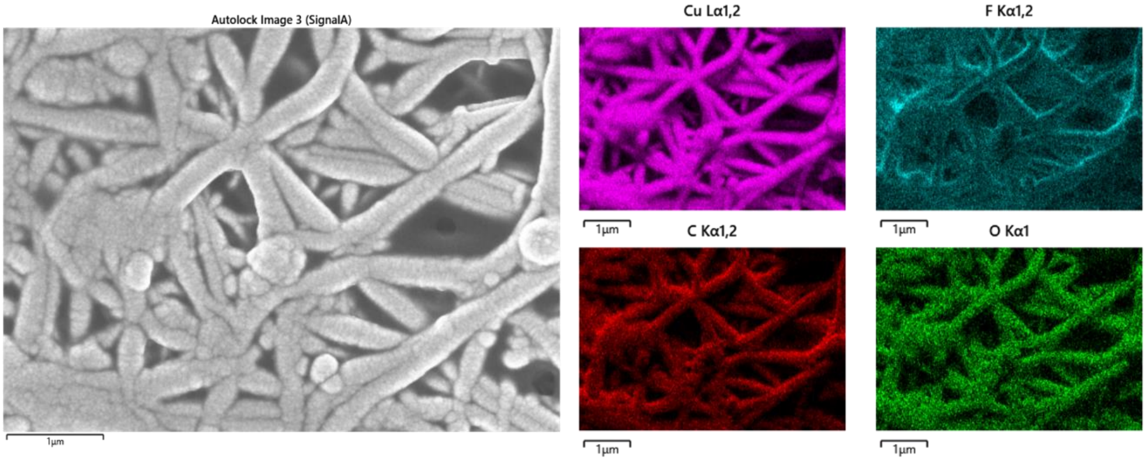

Figure S14. Scanning electron microscopy (SEM) and energy-dispersive X-ray spectroscopy (EDS) elemental mapping of CEI-coated electrode. The left panel presents a high-resolution SEM image, revealing the intricate nanostructured morphology of the CEI sample. The coloured panels on the right panels display EDS elemental maps for key elements (Cu, F, C and O), demonstrating their homogeneous distribution across the structure.

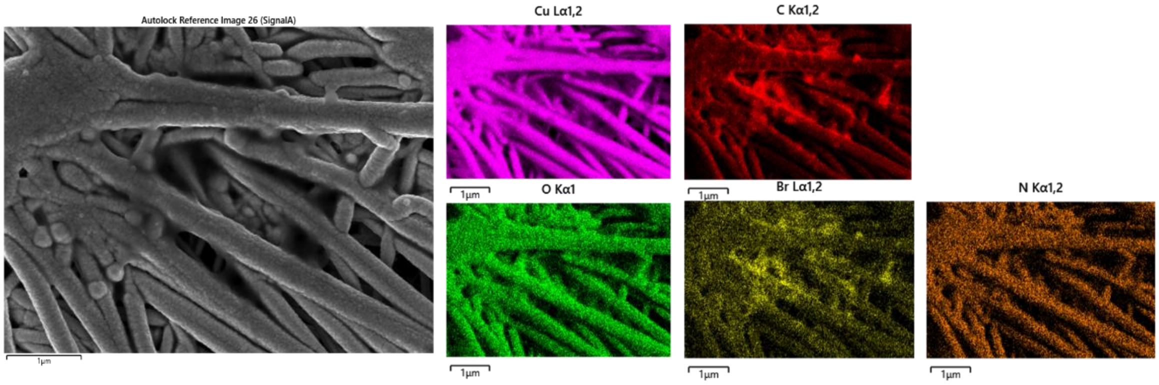

Figure S15. Scanning electron microscopy (SEM) and energy-dispersive X-ray spectroscopy (EDS) elemental mapping of AEI-coated electrode. The left panel presents a high-resolution SEM image, revealing the intricate nanostructured morphology of the AEI sample. The coloured panels on the right display EDS elemental maps for key elements (Cu, C, O, Br and N), demonstrating their homogeneous distribution across the structure.

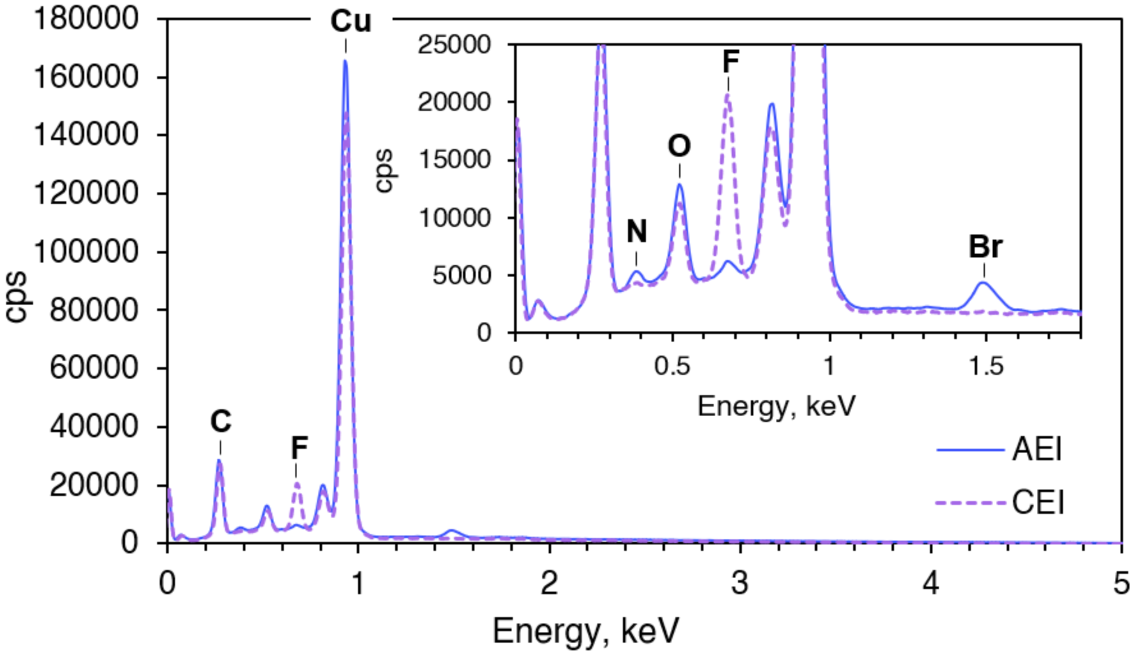

Figure S16. Energy-dispersive X-ray spectroscopy (EDS) spectra of Cu electrodes coated with individual cation exchange ionomer (CEI, dashed purple) or anion exchange ionomer (AEI, solid blue). The N signal in the CEI-only sample is negligible, confirming minimal AEI contamination. Conversely, the F signal in the AEI-only sample is low and may partially originate from the PTFE gas diffusion layer substrate rather than CEI contamination. The inset highlights the N, O, F, and Br peaks at lower energies. These control measurements were used to assess background contributions and ionomer purity.

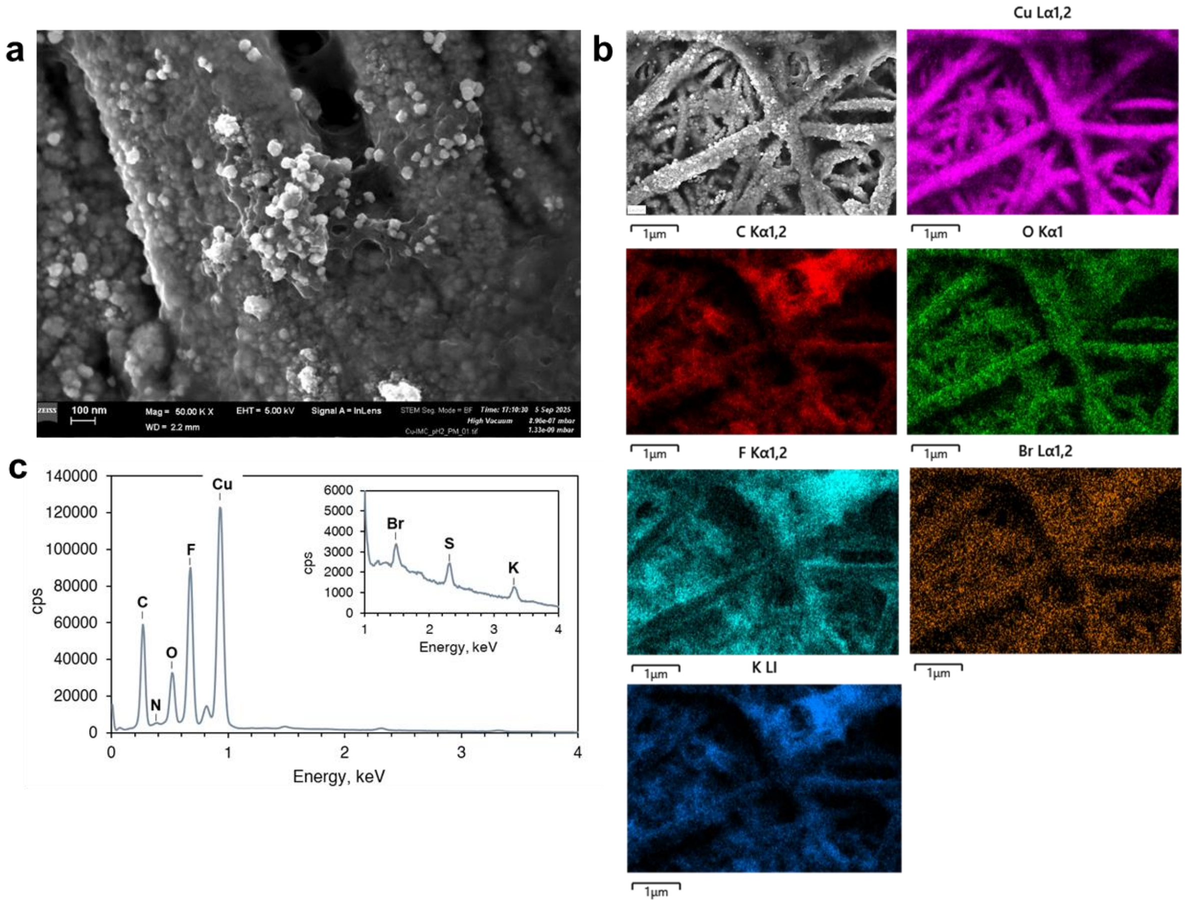

Figure S17. Scanning electron microscopy (SEM) and energy-dispersive X-ray spectroscopy (EDS) elemental mapping of the post-mortem IMC-coated electrode. a, A high-resolution SEM image, revealing the intricate nanostructured morphology of the IMC sample after reaction. **b,** The panels display a lower magnification SEM image and EDS elemental maps for key elements (Cu, C, F, O, Br and K), demonstrating homogeneous ionomer coverage across the Cu catalyst after reaction, as well as K retention in the ionomer from the electrolyte. **c,** EDS energy spectrum, corresponding to the elemental maps. The inset displays a zoomed in region of the spectrum, featuring Br, S and K peaks.

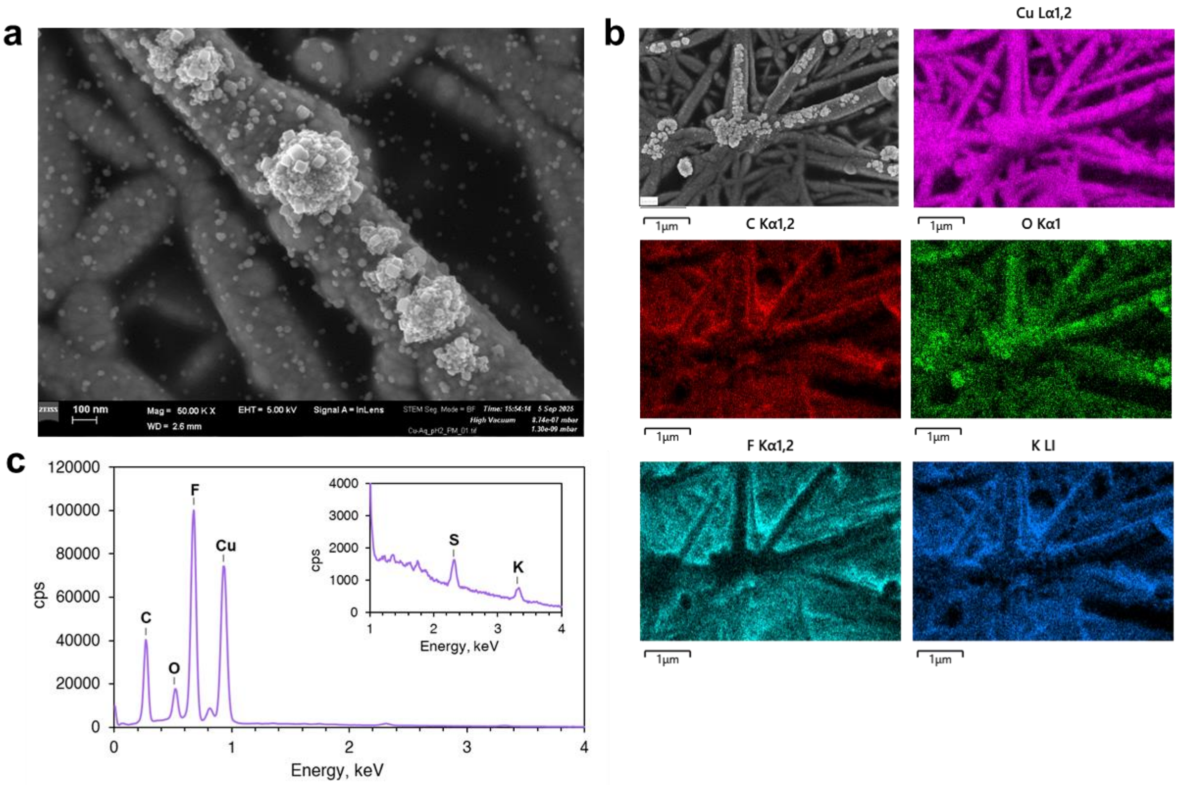

Figure S18. Scanning electron microscopy (SEM) and energy-dispersive X-ray spectroscopy (EDS) elemental mapping of the post-mortem CEI-coated electrode. a, A high-resolution SEM image, revealing the intricate nanostructured morphology of the CEI sample after reaction. **b,** The panels display a lower magnification SEM image and EDS elemental maps for key elements (Cu, C, F, O, and K), demonstrating homogeneous ionomer coverage across the Cu catalyst after reaction, as well as K retention in the ionomer from the electrolyte. **c,** EDS energy spectrum, corresponding to the elemental maps. The inset displays a zoomed in region of the spectrum, featuring S and K peaks. Ex situ SEM/EDS indicates that the observed surface deposits are Cu-rich, oxygen-bearing ($Cu–O_x$), as evidenced by the strong co-localization of Cu and O signals. Because the analysis is post-electrolysis and conducted in air, we do not assign a specific phase and instead refer to them generically as Cu–oxygen species.

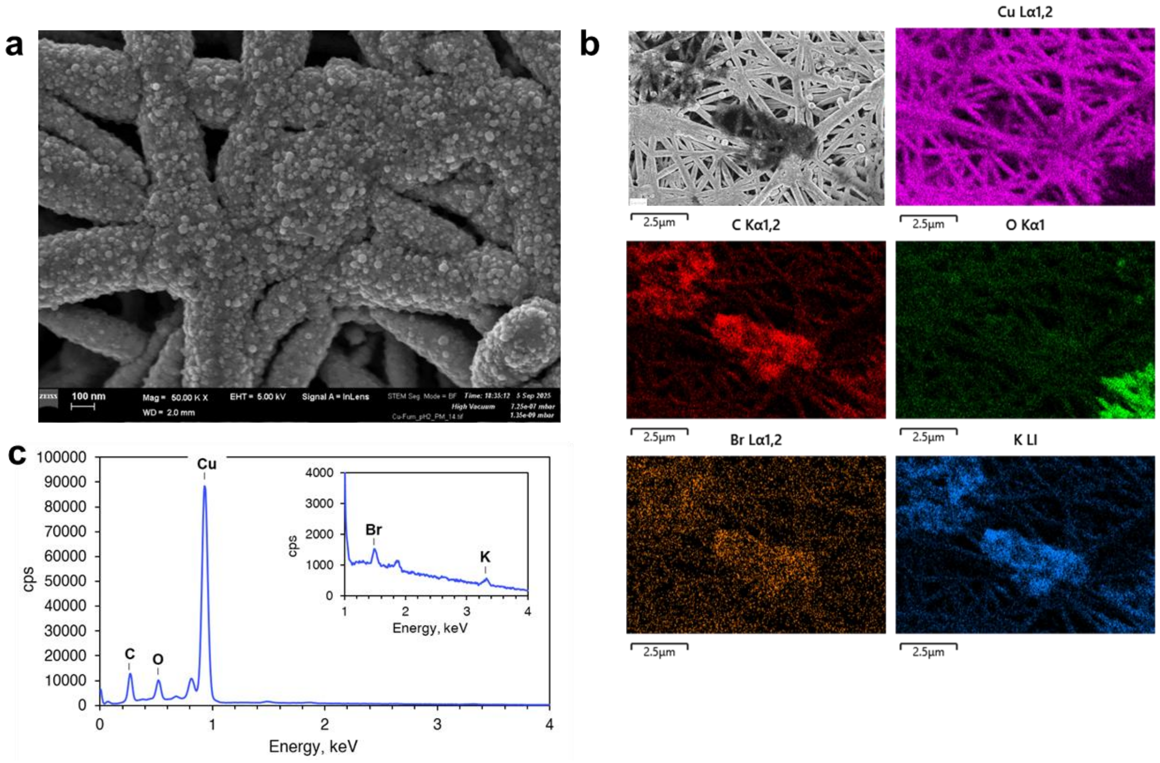

Figure S19. Scanning electron microscopy (SEM) and energy-dispersive X-ray spectroscopy (EDS) elemental mapping of the post-mortem AEI-coated electrode. a, A high-resolution SEM image, revealing the intricate nanostructured morphology of the AEI sample after reaction. **b,** The panels display a lower magnification SEM image and EDS elemental maps for key elements (Cu, C, Br, O, and K), demonstrating inhomogeneous ionomer coverage across the Cu catalyst after reaction, as well as K retention only in the parts where the ionomer remains. **c,** EDS energy spectrum, corresponding to the elemental maps. The inset displays a zoomed in region of the spectrum, featuring Br and K peaks.

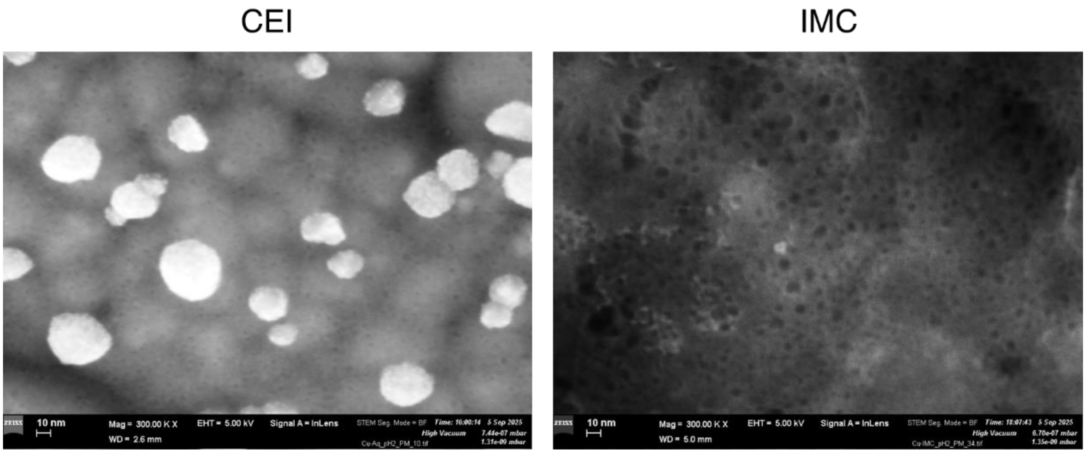

Figure S20. Nanoporous structure of the ionomer coating on the post-mortem electrodes. The left panel presents a high-resolution SEM image, revealing the CEI-coated post-mortem Cu catalyst with redeposited Cu–O_x species on the surface. The ionomer coating features a porous structure of ~ 1 nm pore size. The right panel displays a high-resolution SEM image of IMC-coated post-mortem catalyst, featuring a porous structure with the pore size ~10 nm. The AEI-coated post-mortem electrode

FT-IR measurements

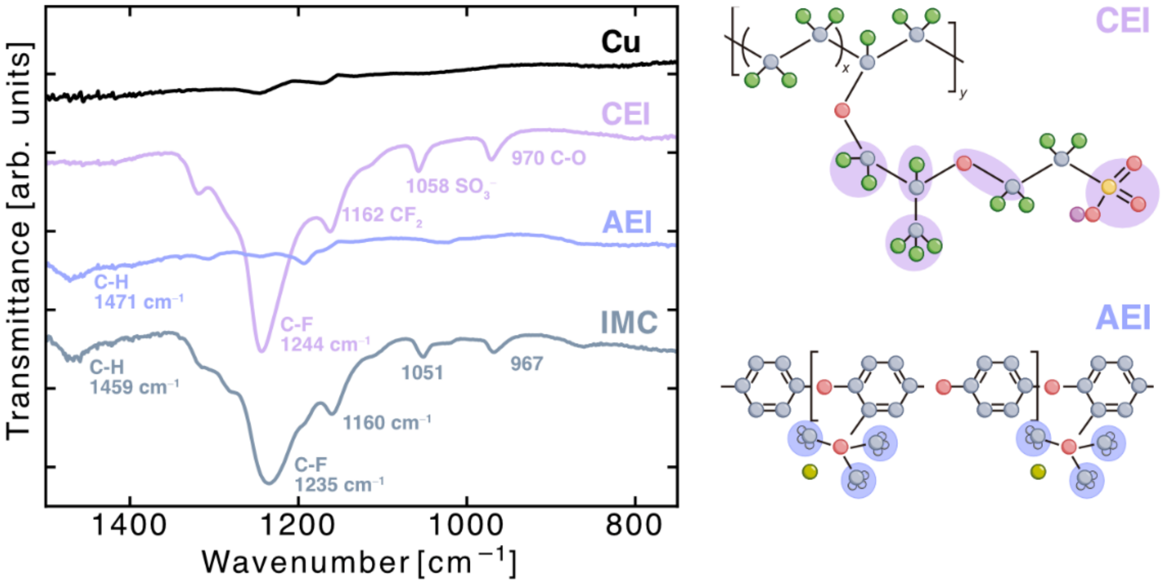

Figure S21. FT-IR analysis of ionomer interactions. FT-IR measurements reveal interactions between CEI (Aquivion chemical structure shown in grey) and AEI (Fumion chemical structure shown in brown) ionomers, as evidenced by shifts in the C–H and C–F vibrational bands, indicating changes in their molecular environment.

Kelvin Probe Force Microscopy (KPFM)

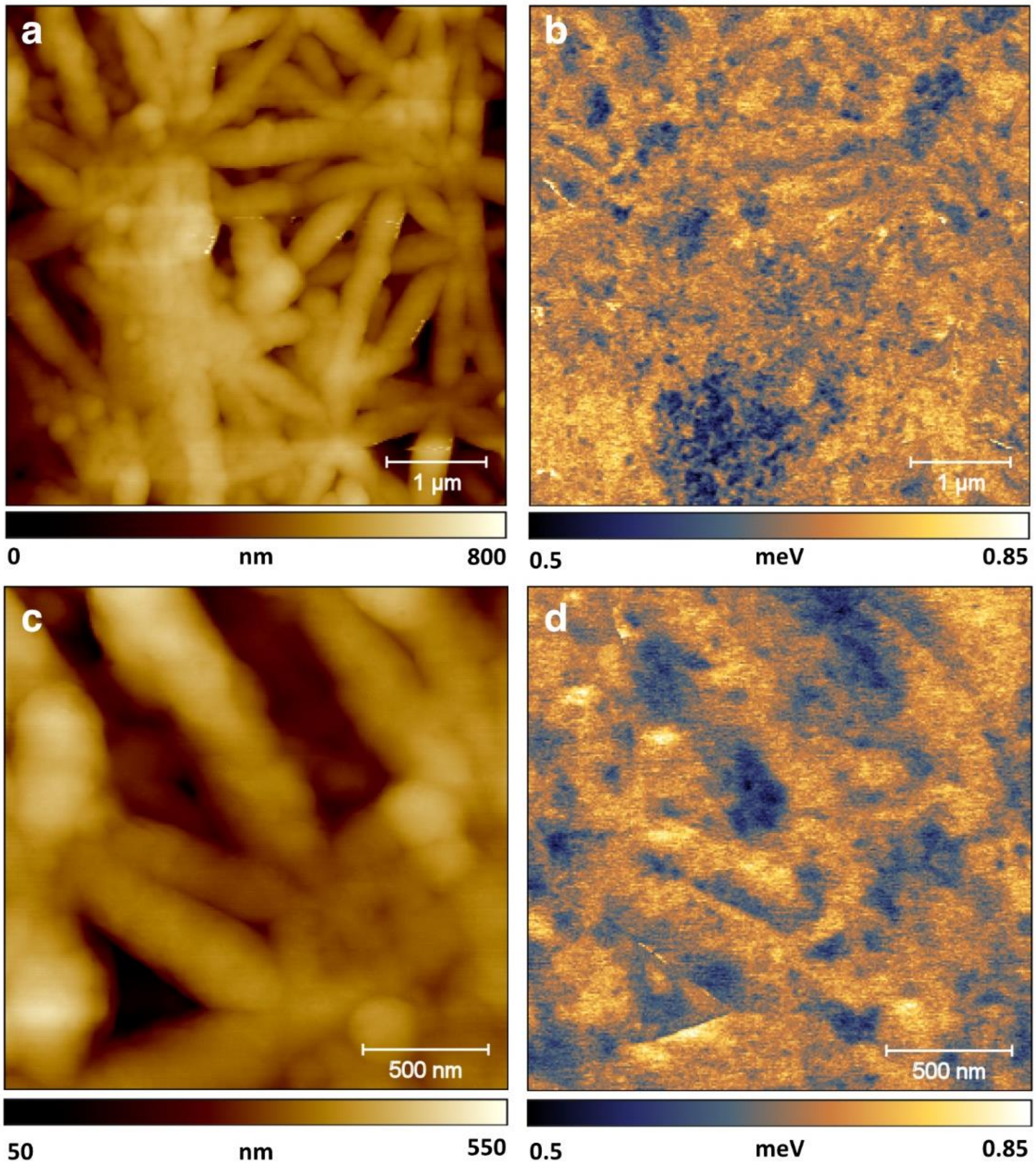

Figure S22. Kelvin Probe Force Microscopy (KPFM) measurements of CEI-coated Cu/PTFE gas diffusion electrodes (GDEs), showing surface topography (a, c) and corresponding work function maps (b, d). The CEI layer forms a homogeneous coating, with only minor inhomogeneities near the fiber regions. A work function increase of ~ 0.1 eV relative to bare Cu is observed, attributed to the formation of an interfacial dipole from chemisorbed SO_3^- end-groups. This dipole, oriented into the surface, withdraws electron density from the interface, deepening the Fermi level and enhancing the electron-accepting character of the coated electrode. The KPFM measurements on CEI were collected with a PtIr coated tip (NuNano Spark 70).

Note S1. KPFM report on CEI and AEI-coated electrodes

Aquivion (CEI) and Fumion (AEI) vary fundamentally in chemistry and mechanics, which makes KPFM measurements behave differently. Aquivion is a short-side-chain perfluorosulfonic-acid (PFSA) ionomer whose fully fluorinated matrix retains semi-crystalline order and a Young's modulus in the 0.3–1 GPa range, which persists even after hydration.[3] On the other hand, Fumion is a quaternary-ammonium poly(phenyleneoxide) where residual solvents and absorbed water plasticise the film, cutting its modulus by up to ~95 % and leaving a soft layer that presents positive charge at the surface.[4] That makes the AEI coating much softer, less well adhered and with a positive fixed charge which are all prerequisites for an electrostatically driven "pickup" event.

When a KPFM scan starts, an AC bias (VAC, typically 1–3 V at 17 kHz) and a slowly adjusted DC bias (VDC) between tip and sample is added. The electrostatic force is:

$$F_{es} = -\frac{1}{2}\frac{dC(z)}{dz}\left[V_{DC} + V_{AC}\sin\omega t\right]^2,$$

so the force-gradient at the tip apex can reach $10^7 – 10^9$ N·m^{-2} even for a 1 V excitation because the effective capacitor spacing is only a few nm.

For a soft polymer that carries real net charge, the field exerts both (i) an electrophoretic force on the fixed quaternary-ammonium chains (QA+) toward a negatively biased tip, and (ii) a dielectrophoretic force on any polarizable solvent rich domains even if the net charge was zero. Capture of nanoparticles with exactly this mechanism has been demonstrated with conductive AFM tips.[5,6]

Therefore, there is a chain of events that happens while conducting KPFM measurements on the AEI coated GDEs: Initially, when applying the electrostatic field, the surface bulges locally which in turn forms a meniscus with the conductive tip. This polymer-tip interface, supplemented by AC field oscillations, shear the meniscus depositing a uniform film on the PtIr coated tip. This has the effect of dulling the tip while drifting the contact potential difference (CPD). After a few scan lines, the tip is completely insulated and the KPFM feedback can no longer null the electrostatic force (image collapses).

CEI coated electrodes do not foul the probe because their sulfonate groups chemisorb strongly on the Cu-based GDE and pin the stiff PFSA network, so the electrostatic stress never exceeds the film's yield threshold. In contrast, AEI's cationic backbone adheres only weakly, leaving the soft, positively charged film free to migrate under the same field.[7]

In the case of the mixed ionomer coating (IMC), initially it appears stable because the stiff, low-surface-energy CEI forms a thin layer that anchors to the Cu GDE and shields the softer AEI below. However, during extended KPFM scanning, the intense, localized electric field exerts electrophoretic and dielectrophoretic forces on the QA segments and solvent-rich AEI domains, steadily carrying them upward through the PFSA matrix.[8] Each line retrace thins the PFSA cover, the newly exposed AEI swells, softens, and further enriches the contact zone, until the surface behaves like a pure AEI film.[9] For that reason, KPFM for IMC coatings were collected in higher speeds and were normalized for any CPD drift (Fig 2c, main text).

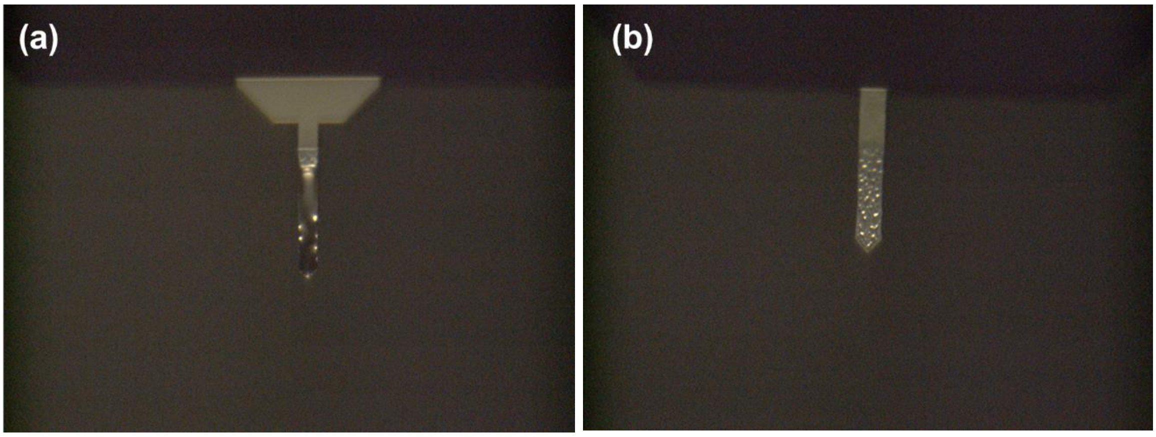

Figure S23. **Snapshots of the conductive tips after conducting KPFM measurement**s on (a) AEI and (b) IMC coatings respectively. In the case of AEI, the deposition is more abrupt than the gradual deposition of IMC.

X-ray Photoelectron Spectroscopy (XPS)

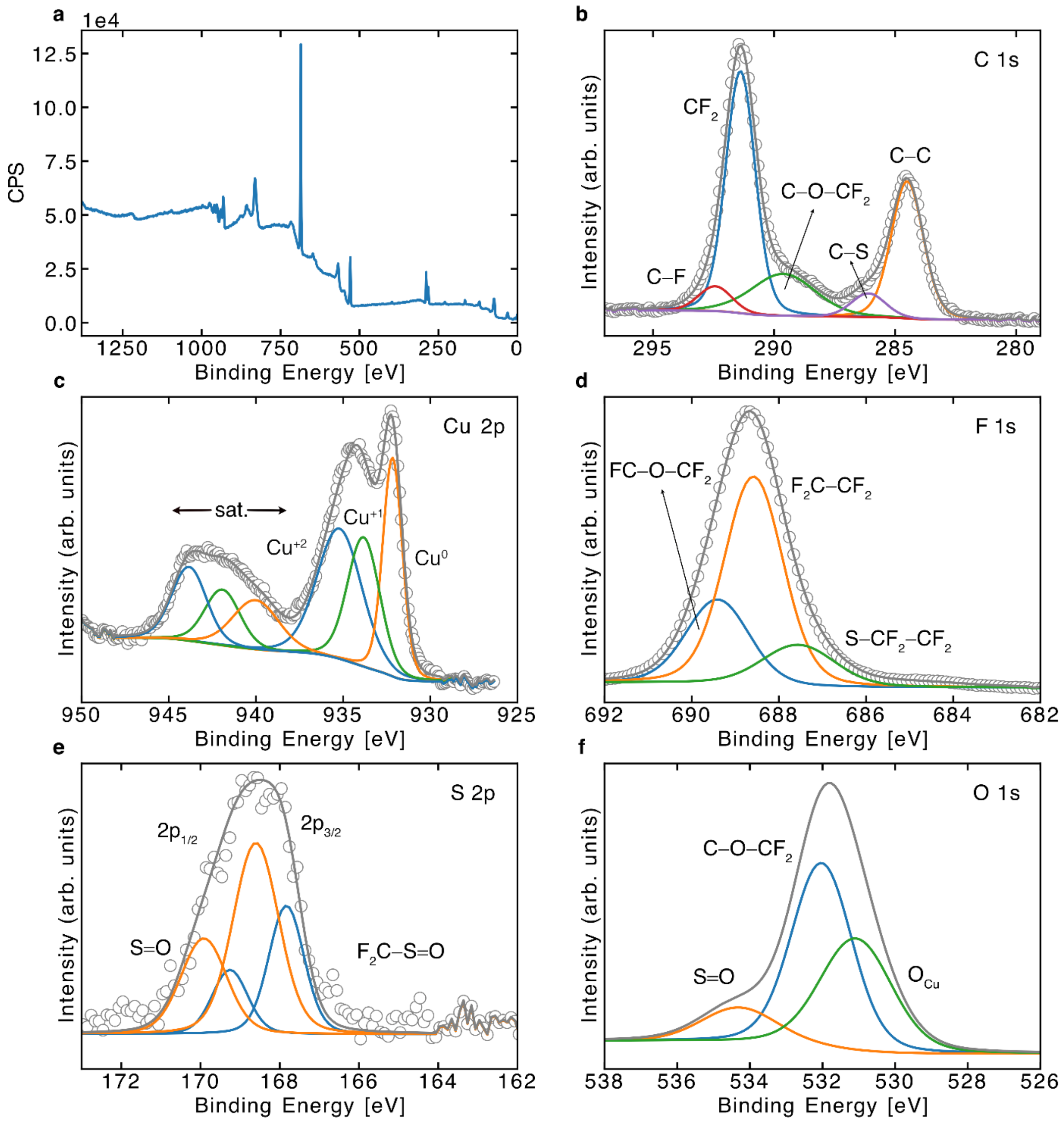

Figure S24. XPS spectra of CEI-coated electrodes. **a,** Survey spectrum and high-resolution spectra for **b**, C 1s **c**, Cu 2p **d**, F1s **e**, S 2p and **f**, O1s. Measurements were conducted using a SPECS PHOIBOS 150, and data fitting was performed using Casa XPS software.

Table S1. **XPS peak fitting for CEI-coated electrodes.**[10–13] The binding energy of all peaks were corrected with respect to C1s peak (284.5 eV).

**C 1s**	**eV**
C-C	287.5
C-O-CF_2	289.6
C-S	286.1
CF	292.4
CF_2	291.4
**Cu 2p**	**eV**
Cu(0)	932.2
Cu(I)	933.8
Cu(II)	935.2
**F 1s**	**eV**
S-CF_2-CF_2	687.5
F_2C-CF_2	688.6
CF-O-CF_2	689.4
**S 2p**	**eV**
F_2C-S=O $2p^{3/2}$	167.8
F_2C-S=O $2p^{1/3}$	169.2
O-S-O $2p^{3/2}$	168.6
O-S-O $2p^{1/3}$	168.2
**O 1s**	**eV**
Cu-O	531.1
C-O-CF_2	532.0
S-O	534.3

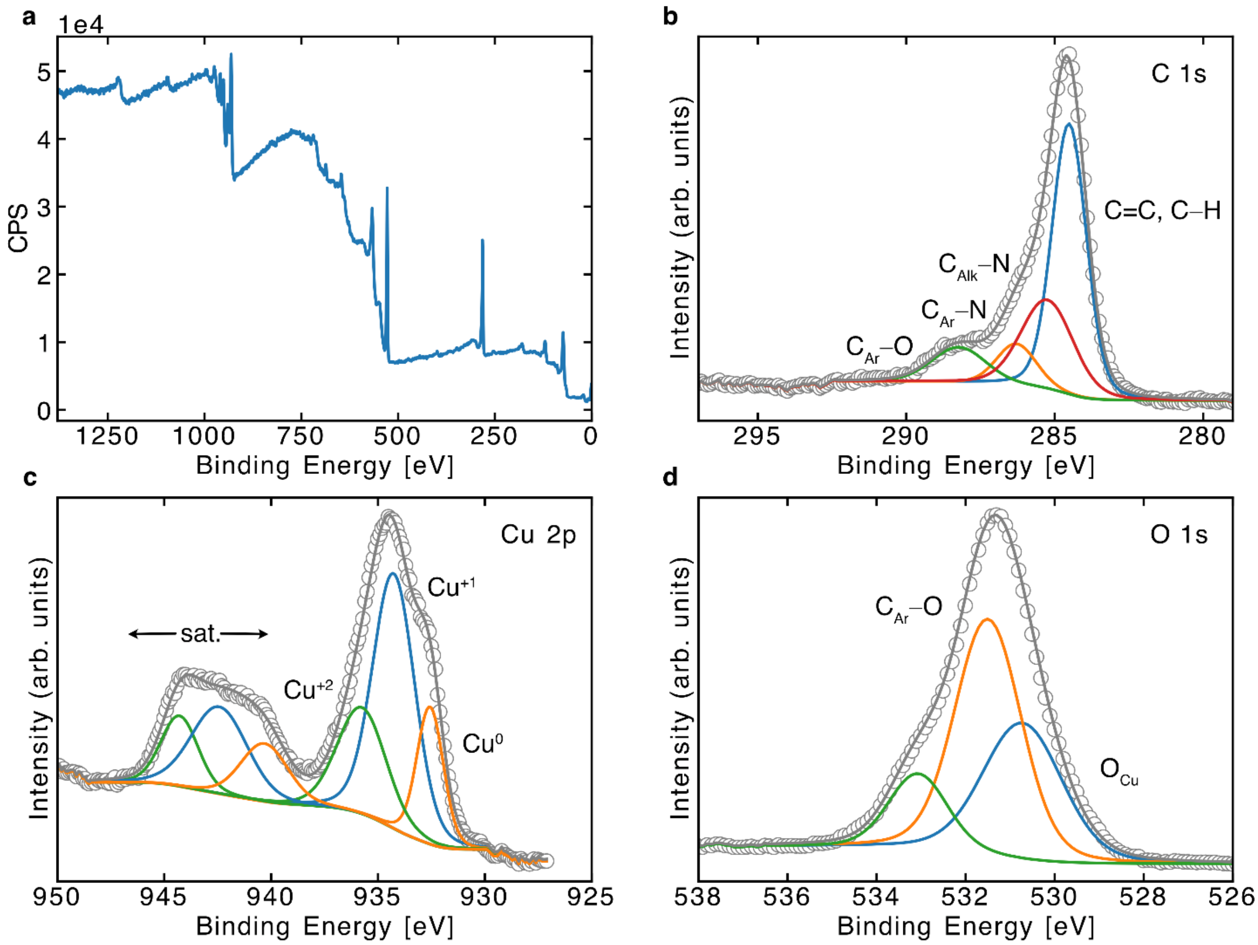

Figure S25. XPS spectra of AEI-coated electrodes. **a,** Survey spectrum and high-resolution XPS spectra for **b**, C 1s **c**, Cu 2p and **d**, O 1s. Measurements were conducted using a SPECS PHOIBOS 150, and data fitting was performed using Casa XPS software.

Table S2. **XPS peak fitting for AEI-coated electrodes.**[10–13] The binding energy of all peaks were corrected with respect to C1s peak (284.5 eV).

**C 1s**	**eV**
C-C	284.5
C-O (Aryl)	288.2
C-N (Aryl)	286.3
C-N (Alkyl)	285.2
**Cu 2p**	**eV**
Cu(0)	932.6
Cu(I)	934.3
Cu(II)	935.8
**O 1s**	**eV**
Cu-O	530.7
Aryl C-O	531.5
O H_2O	533.1

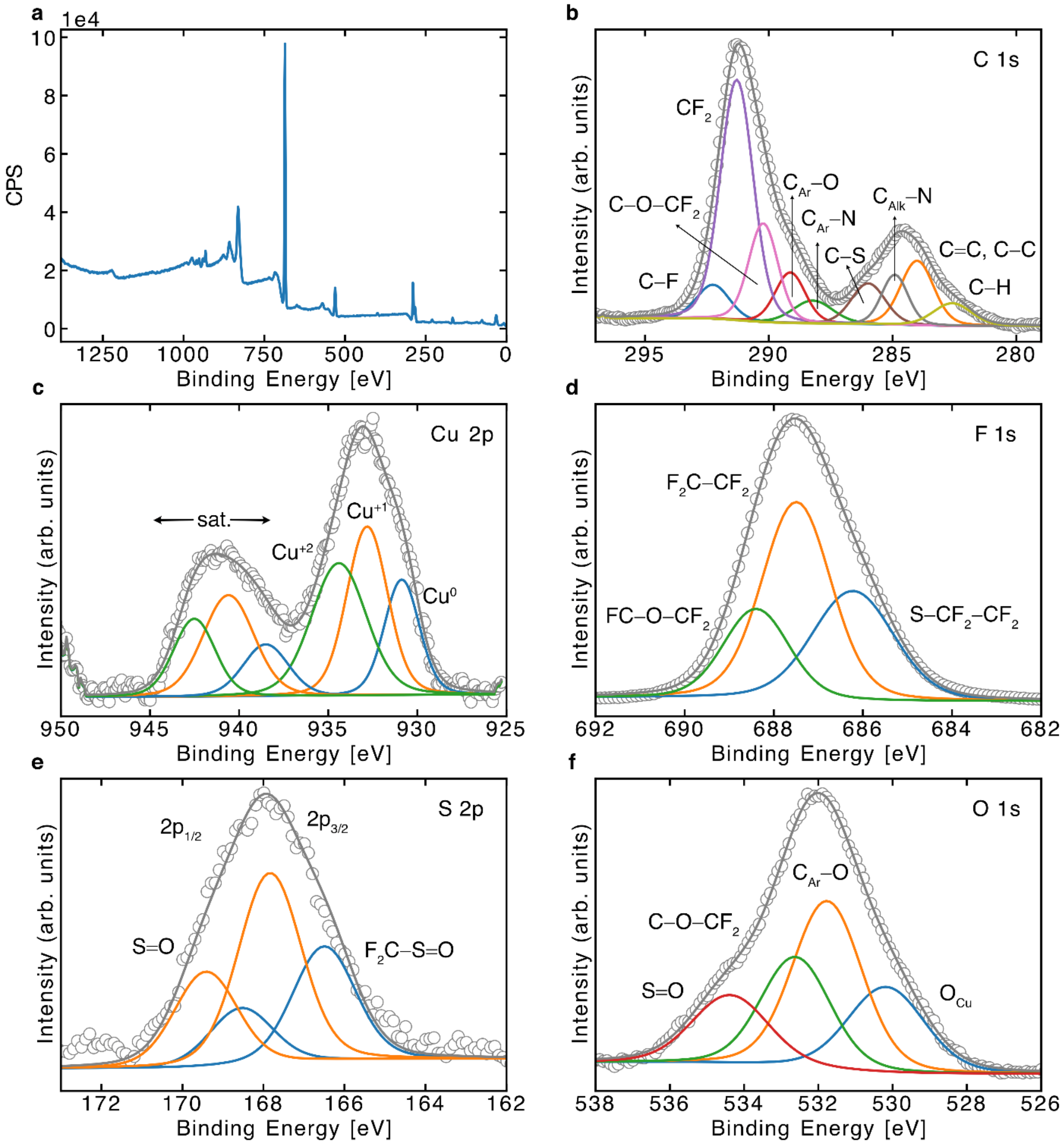

Figure S26. XPS spectra of IMC-coated electrodes. **a,** Survey spectrum and high-resolution spectra for **b**, C 1s **c**, Cu 2p **d**, F1s **e**, S 2p and **f**, O1s. Measurements were conducted using a SPECS PHOIBOS 150, and data fitting was performed using Casa XPS software.

Table S3. **XPS peak fitting for IMC-coated electrodes.**[10–13] The binding energy of all peaks were corrected with respect to C1s peak (284.5 eV).

**C 1s**	**eV**
C-C	284.0
C-O-CF_2	290.2
C-S	286.0
CF	292.2
CF_2	291.3
C-O (Aryl)	289.1
C-N (Aryl)	288.2
C-N (Alkyl)	284.9
**Cu 2p**	**eV**
Cu(0)	930.9
Cu(I)	932.8
Cu(II)	934.4
**F 1s**	**eV**
S-CF_2-CF_2	686.2
F_2C-CF_2	687.5
CF-O-CF_2	688.4
**S 2p**	**eV**
F_2C-S=O $2p^{3/2}$	166.5
F_2C-S=O $2p^{1/3}$	168.5
O-S-O $2p^{3/2}$	167.8
O-S-O $2p^{1/3}$	169.4
**O 1s**	**eV**
Cu-O	530.2
C-O-CF_2	532.6
S-O	534.4
Aryl C-O	531.8

Contact angle measurements

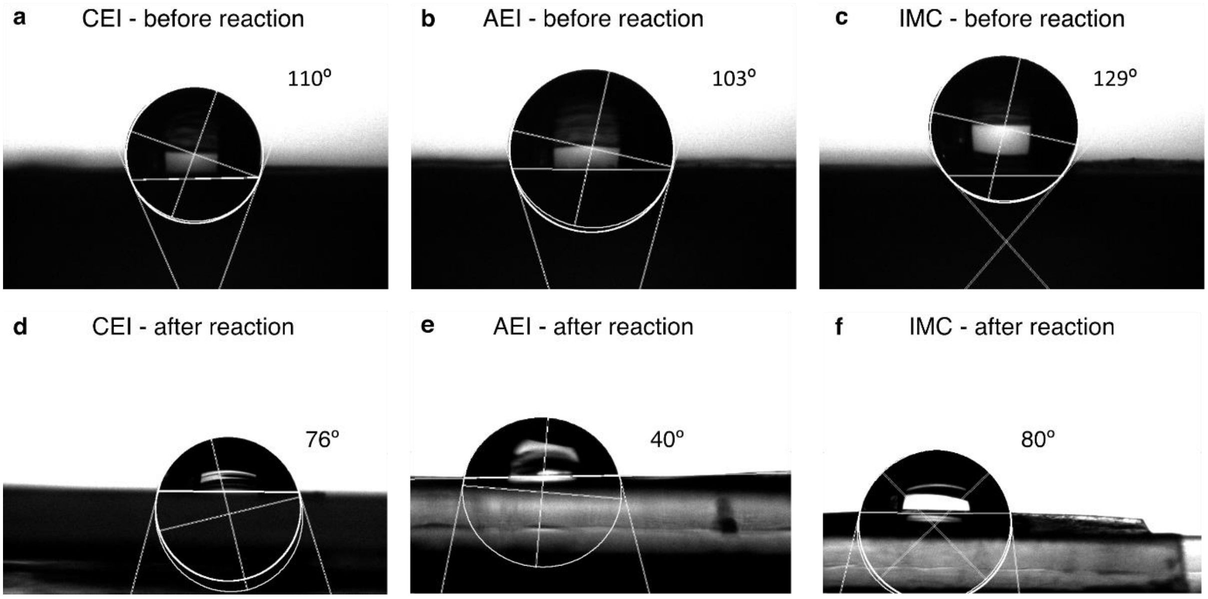

Figure S27. Contact angle measurements of ionomer-coated samples. Contact angle measurements for **a**, CEI **b**, AEI, and **c**, IMC samples before reaction. We observe an increased hydrophobic character in the IMC sample, indicating changes in surface wettability due to the combined ionomer configuration. Contact angle measurements for **d**, CEI **e**, AEI, and **f**, IMC samples after reaction.

Voltammetric Characterization

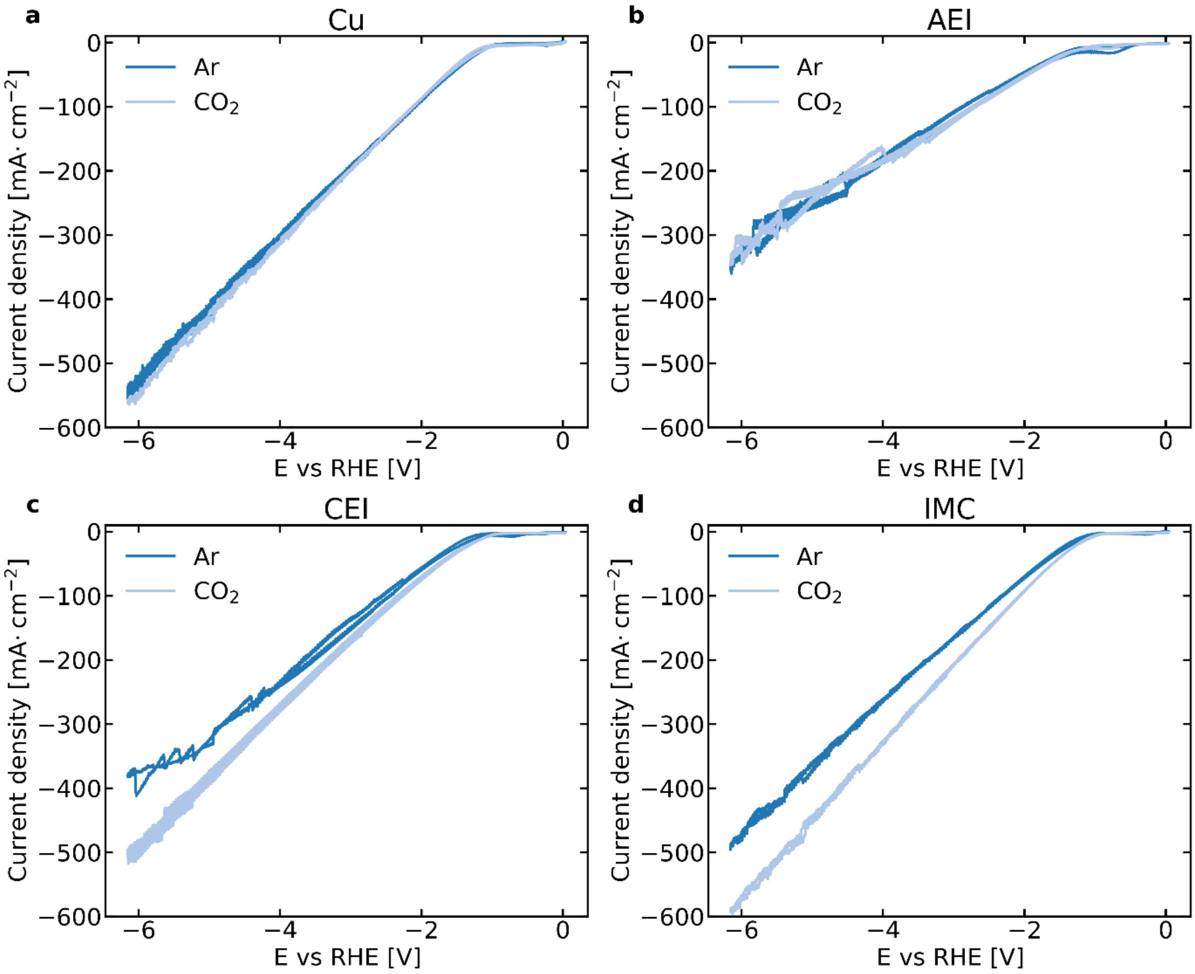

Figure S28. Cyclic voltammetry of Cu-based electrodes with Ar and CO_2 as the feeding gas. CV curves of **a** bare Cu, **b** AEI-coated Cu, **c** CEI-coated Cu, and **d** IMC electrodes recorded in Ar (dark blue) and CO_2 (light blue) at a scan rate of 100 mV·s^{-1} in 0.5 M K_2SO_4 + H_2SO_4 (pH = 2). Enhanced current densities under CO_2 conditions for CEI and IMC suggest increased CO_2E selectivity over HER.

Electrochemical Impedance Spectroscopy (EIS)

Simple Randles Circuit

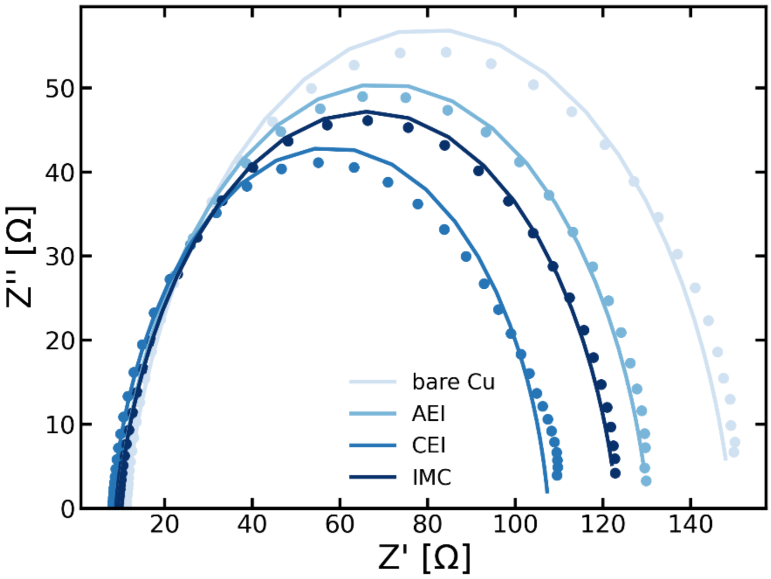

Figure S29. Electrochemical Impedance Spectroscopy (EIS) measurements (dots) and fittings (solid lines) for effective EDL capacitance. Bare Cu, AEI, CEI and IMC in K_2SO_4 0.5 M + H_2SO_4 (pH = 2) catholyte and 0.5 M H_2SO_4 anolyte. EIS was carried out purging Ar as the feeding gas at -0.31 V vs RHE. The EIS data were obtained between 0.1 Hz and 10 kHz. The data was fitted using the simple Randles circuit. The effective double-layer capacitance (C_{dl}) was obtained from the constant phase element (CPE) parameters and the two resistances using the Brug formula.

Note S2. Electric double layer (EDL) capacitance calculation

The effective double-layer capacitance (C_{dl}) was obtained from the constant phase element (CPE) parameters and the two resistances using the Brug formula:

$$C_{dl} = \frac{1}{Y_0^N}\left(\frac{1}{R_S} + \frac{1}{R_{ct}}\right)^{\frac{N-1}{N}}$$

where R_s is the solution resistance, R_{ct} is the charge transfer resistance, Y_0 is the CPE constant and N is the CPE exponent, indicating the deviation from ideal capacitive behaviour ($0 \leq N \leq 1$).

Table S4. EIS fitting parameters by using the simple Randles equivalent circuit:

	R_s [Ω]	R_{ct} [Ω]	Y_0 [μMho^N]	N	C_{dl} [μF]
**Cu**	11.1	138	483	0.877	229.5
**AEI**	9.33	121	525	0.885	260.5
**CEI**	8.04	99.6	480	0.905	265.7
**IMC**	9.31	114	635	0.880	312.1

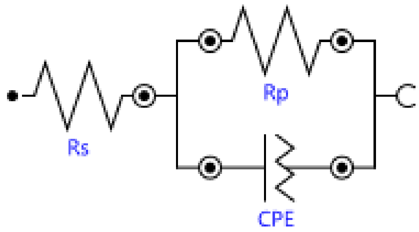

Mechanistically Informed Equivalent Circuit Model

Bare Cu, AEI, CEI, and IMC in K_2SO_4 0.5 M + H_2SO_4 (pH = 2) catholyte and 0.5 M H_2SO_4 anolyte. EIS was carried out by purging Ar as the feeding gas at -0.31 V vs RHE. The EIS data were obtained between 0.1 Hz and 10 kHz and analyzed using EIS Data Analysis 1.2 software.[14] The analysis presented here excludes the real surface area and is related to an "average" active site; the overall current will depend on the number of those sites.[15,16]

For the spectra fitting, the following equivalent electric circuit was used (**Figure S30**):[17,18]

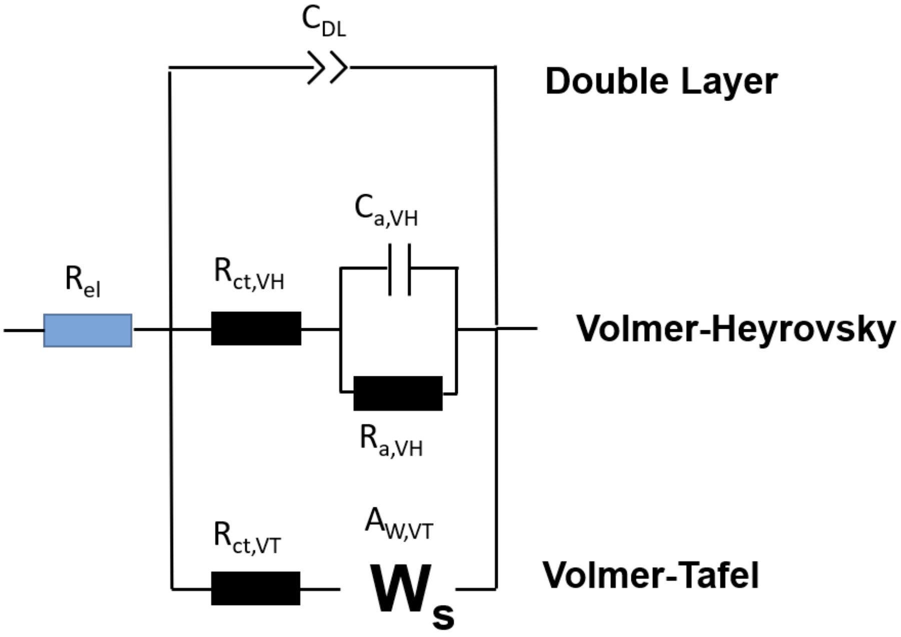

Figure S30. The equivalent electric circuit used for the fitting of impedance data. The first electroactive step (Volmer step) of the two mechanisms of hydrogen evolution is the same: the formation of the adsorbed hydrogen, which might occur with some diffusion contribution (classical R-Ws branch, where Ws is "Warburg short" for finite length diffusion). The Tafel step is not electroactive. So, the second branch in parallel only appears if, at the active site (occupied by hydrogen), another proton is reduced (assumed not diffusion limited, Heyrovsky mechanism). Therefore, only one Warburg element is used.

A typical impedance spectrum, together with the fitting for the case of pure Cu, is shown in **Figure S31**.

a

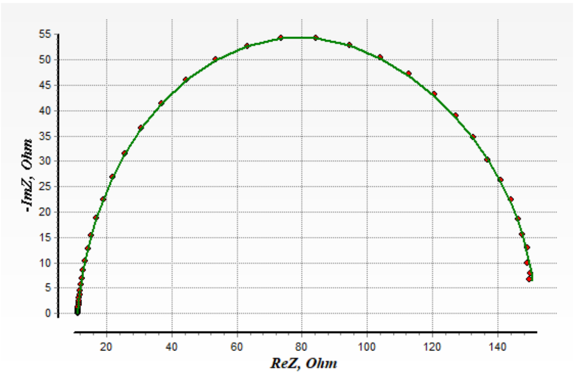

b

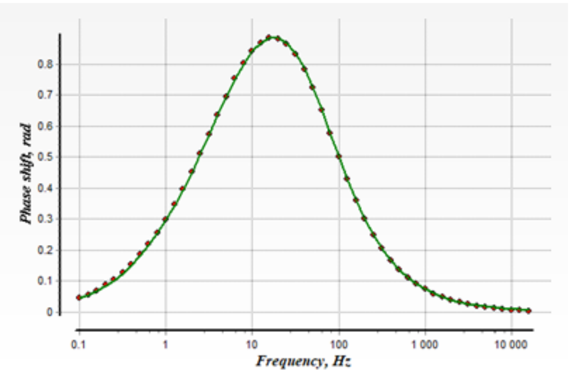

c

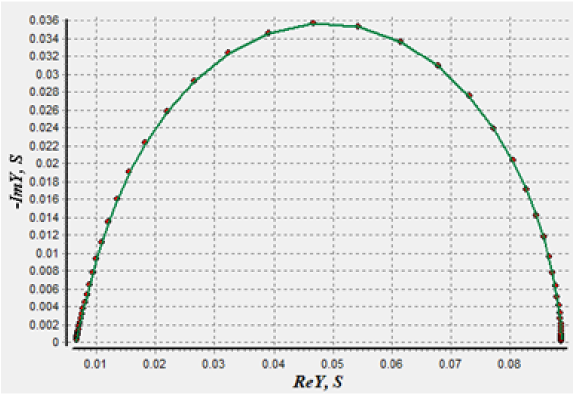

d

Assume, that in water D_{H+}= 9.3 x 10^{-5} cm^2/s

Nernst diffusion layer thickness = W_{sc1} x $(D_{H+})^{0.5}$ =

= **67 ± 2 μm**

For the Volmer step:

$K_{app}/(2D_{H+})^{0.5}$= W_{wsr1}/R_2 = 0.3123$s^{-0.5}$

K_{app}= **4.3 ± 0.2 x 10^{-3} cm/s (related to a single site)**

Two mechanisms almost equally contribute: R_3/R_2 = VT/VH = **0.82**

e

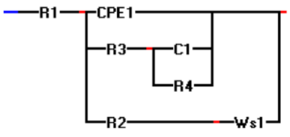

Normalized RMSD = 0.25%

Parameters	Result/In.Val.	Error %
C1	0.00072443	6.6254
R1	11.244	0.15297
R2	240.01	0.52711
R3	198.47	0.47127
R4	55.788	1.917
P1	0.00037029	0.26054
n1	0.9232	0.055008
Wsr1	74.961	2.3693
Wsc1	0.69599	2.8223

Figure S31. EIS spectrum for bare Cu in **a** Nyquist impedance, **b** Bode phase, and **c** Nyquist admittance representations together with the fitting to the equivalent circuit shown in **Figure S30**. Estimation of important kinetic parameters is shown in **d**. The fitting parameters with individual parameter errors are represented in **e**.

Figure S32 shows similar sets of data for the case of AEI.

a

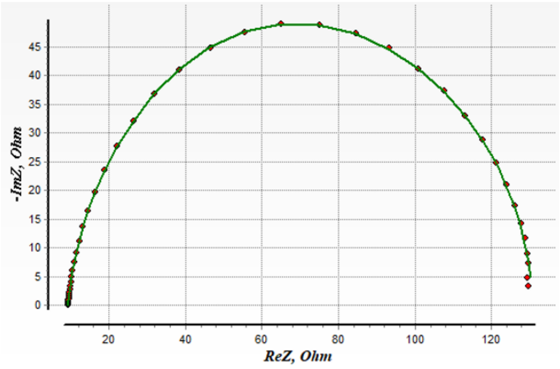

b

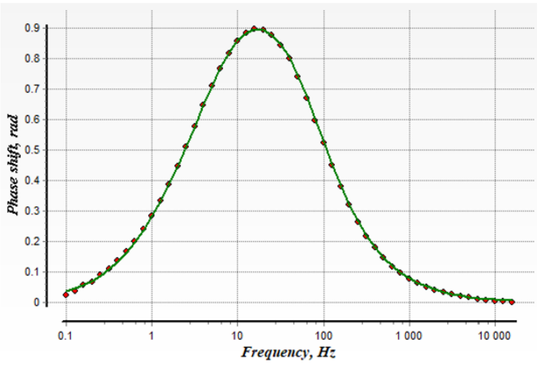

c

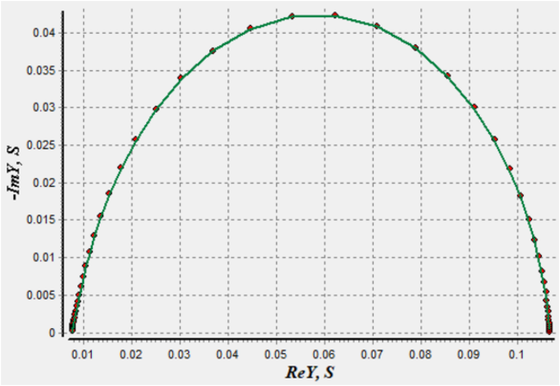

d

Assume, that in water D_{H+}= 9.3 x 10^{-5} cm^2/s

Nernst diffusion layer thickness = W_{sc1} x $(D_{H+})^{0.5}$ =

= **54 ± 3 µm**

For the Volmer step:

$K_{app}/(2D_{H+})^{0.5}$= W_{wsr1}/R_2 = 0.5595$s^{-0.5}$

K_{app}= **7.6 ± 0.4 x 10^{-3} cm/s (related to a single site)**

Two mechanisms do not equally contribute: R_3/R_2 = VT/VH = **0.52**

e

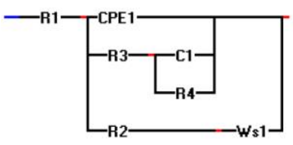

Normalized RMSD = 0.4%

Parameters	Result/In.Val.	Error %
C1	0.00040721	12.892
R1	9.372	0.24391
R2	266.74	1.2601
R3	138.96	0.61363
R4	38.924	2.295
P1	0.00042276	0.40718
n1	0.91948	0.085831
Wsr1	149.25	3.7489
Wsc1	0.55788	4.318

Figure S32. EIS spectrum for AEI in **a** Nyquist impedance, **b** Bode phase, and **c** Nyquist admittance representations together with the fitting to the equivalent circuit shown in **Figure S30**. Estimation of important kinetic parameters is shown in **d**. The fitting parameters with individual parameter errors are represented in **e**.

Figure S33 shows similar sets of data for the case of CEI.

a

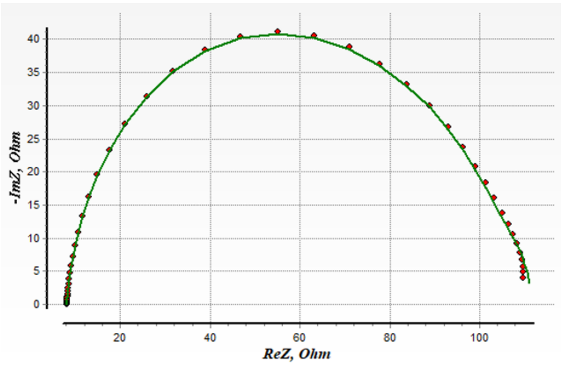

b

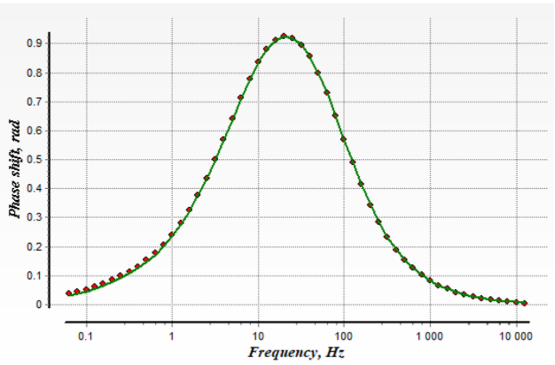

c

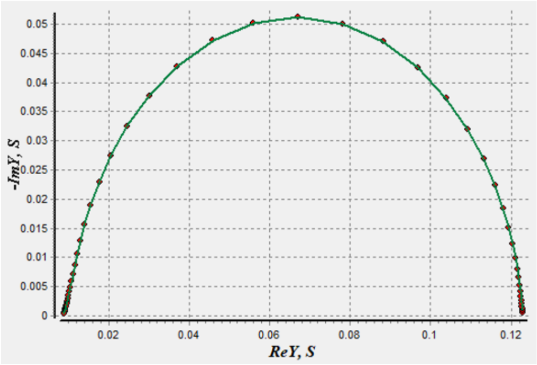

d

Assume, that in water D_{H+}= 9.3 x 10^{-5} cm^2/s

Nernst diffusion layer thickness = W_{sc1} x $(D_{H+})^{0.5}$ =

= **41 ± 2 μm**

For the Volmer step:

$K_{app}/(2D_{H+})^{0.5}$= W_{wsr1}/R_2 = 0.2748$s^{-0.5}$

K_{app}= **3.7 ± 0.2 x 10^{-3} cm/s (related to a single site)**

Two mechanisms do not equally contribute: R_3/R_2 = VT/VH = **4.9**

e

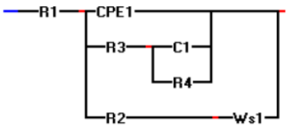

Normalized RMSD = 0.4%

Parameters	Result/In.Val.	Error %
C1	0.0030774	20.411
R1	8.1303	0.31136
R2	103.19	0.49995
R3	502.63	2.4282
R4	233.42	16.559
P1	0.0003945	0.50072
n1	0.93972	0.10063
Wsr1	28.358	3.447
Wsc1	0.42563	3.7228

Figure S33. EIS spectrum for CEI in **a** Nyquist impedance, **b** Bode phase, and **c** Nyquist admittance representations together with the fitting to the equivalent circuit shown in **Figure S30**. Estimation of important kinetic parameters is shown in **d**. The fitting parameters with individual parameter errors are represented in **e**.

Figure S34 shows similar sets of data for the case of the IMC mixture.

a

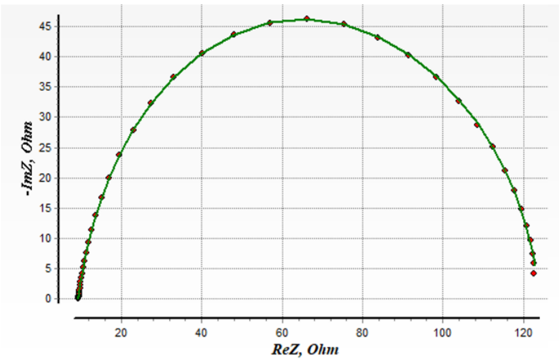

b

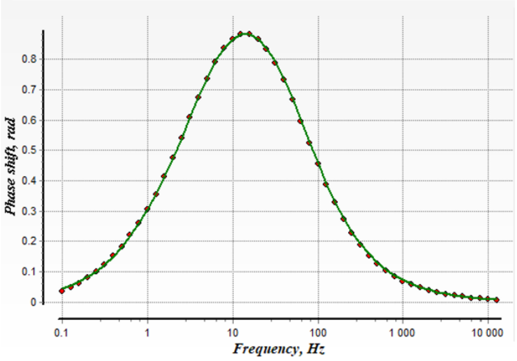

c

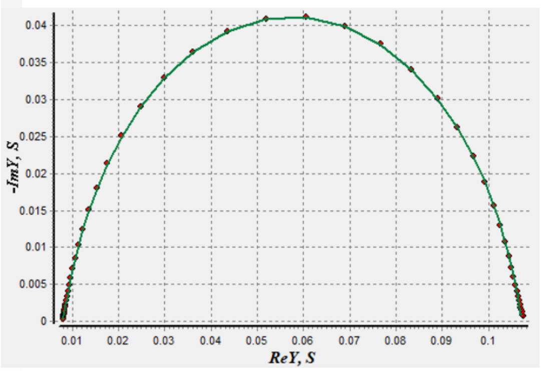

d

Assume, that in water D_{H+}= 9.3 x 10^{-5} cm^2/s

Nernst diffusion layer thickness = W_{sc1} x $(D_{H+})^{0.5}$ =

= **57 ± 4 μm**

For the Volmer step:

$K_{app}/(2D_{H+})^{0.5}$= W_{wsr1}/R_2 = 0.1811$s^{-0.5}$

K_{app}= **2.5 ± 0.2 x 10^{-3} cm/s (related to a single site)**

Two mechanisms do not equally contribute: R_3/R_2 = VT/VH = **0.071**

e

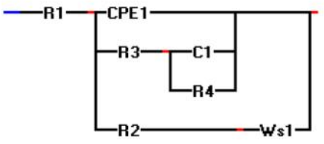

Normalized RMSD = 0.4%

Parameters	Result/In.Val.	Error %
C1	1.9053E-05	8.5973
R1	9.3313	0.24483
R2	196.56	0.72136
R3	14.258	11.147
R4	218.76	0.74059
P1	0.00057467	0.41451
n1	0.88886	0.094528
Wsr1	35.59	5.9115
Wsc1	0.5873	6.6027

Figure S34. EIS spectrum for IMC in **a** Nyquist impedance, **b** Bode phase, and **c** Nyquist admittance representations together with the fitting to the equivalent circuit shown in **Figure S30**. Estimation of important kinetic parameters is shown in **d**. The fitting parameters with individual parameter errors are represented in **e**.

In situ Raman Spectroscopy

Table S5. *In situ* Raman wavenumber and corresponding intermediate/species.[19–24]

**Wave number (cm^{-1})**	**Correspondence**
295 – 303	ρ(Cu–*CO)
382 – 389	ν(Cu–*CO)
430 – 470	Cu-*OH
490 – 520	*C-intermediate
525 – 550	Cu-*$(OH)_y$
614 - 624	Cu-O_{ads}
~730	C-F (ionomer backbone)
~977	SO_4^{2-}
~1062	*CO_3^{2-}
~1015	*HCO_3^-
~1400	*O–C–(X)
1607 – 1637	H_2O
1900 – 2100	*CO
3000 – 3700	H – OH stretching

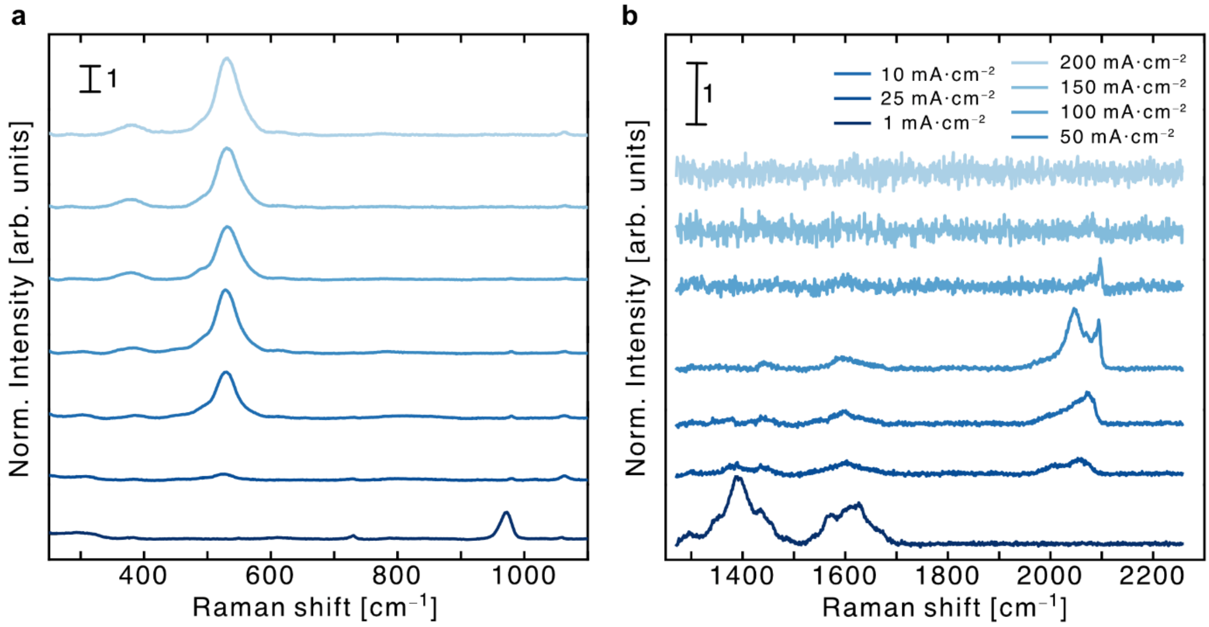

Figure S35. *In situ* normalised Raman spectra of CEI. The normalised Raman spectra of CEI at two different regions **a** R1 (250 to 1100 cm^{-1}), and **b** R2 (1250 to 2300 cm^{-1}) show the presence of specific intermediates and groups. Samples were operated in 0.5 M K_2SO_4 (pH = 2) in a flow cell under increasing current densities from 1 to 200 $mA{\cdot}cm^{-2}$.

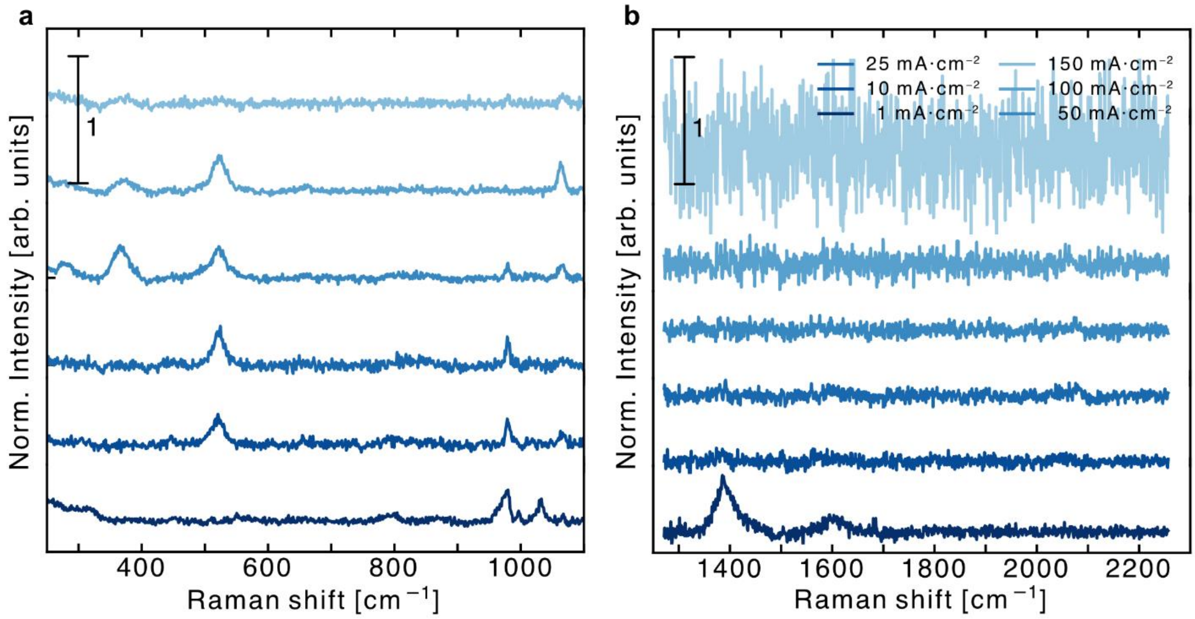

Figure S36. ***In situ* normalised Raman spectra of AEI.** The normalised Raman spectra of AEI at two different regions **a** R1 (250 to 1100 cm^{-1}), and **b** R2 (1250 to 2250 cm^{-1}) show the presence of specific intermediates and groups. Samples were operated in 0.5 M $K_2SO_4 + H_2SO_4$ (pH = 2) in a flow cell under increasing current densities from 1 to 150 $mA \cdot cm^{-2}$.

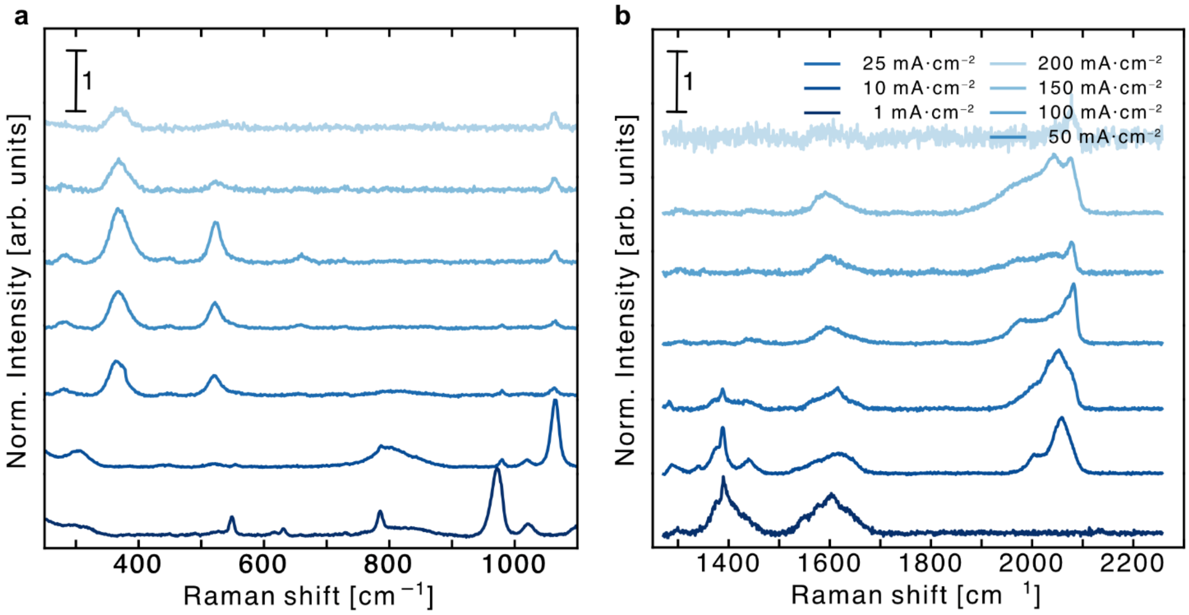

Figure S37. *In situ* normalised Raman spectra of IMC. The normalised Raman spectra of IMC at two different regions **a** R1 (250 to 1100 cm^{-1}), and **b** R2 (1250 to 2300 cm^{-1}) show the presence of specific intermediates and groups. Samples were operated in 0.5 M $K_2SO_4 + H_2SO_4$ (pH = 2) in a flow cell under increasing current densities from 1 to 200 $mA \cdot cm^{-2}$.

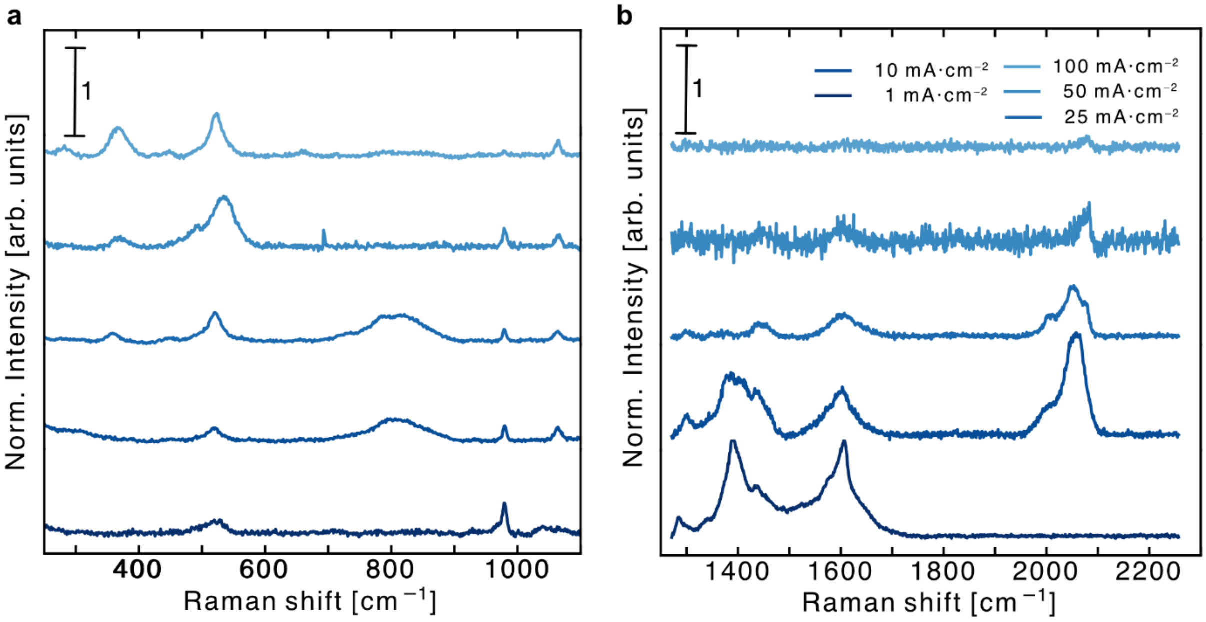

Figure S38. ***In situ* normalised Raman spectra of bare Cu/PTFE.** The normalised Raman spectra of bare Cu/PTFE at two different regions **a** R1 (250 to 1100 cm^{-1}), and **b** R2 (1250 to 2300 cm^{-1}) show the presence of specific intermediates and groups. Samples were operated in 0.5 M K_2SO_4 + H_2SO_4 (pH = 2) in a flow cell in a flow cell under increasing current densities from 1 to 100 $mA \cdot cm^{-2}$.

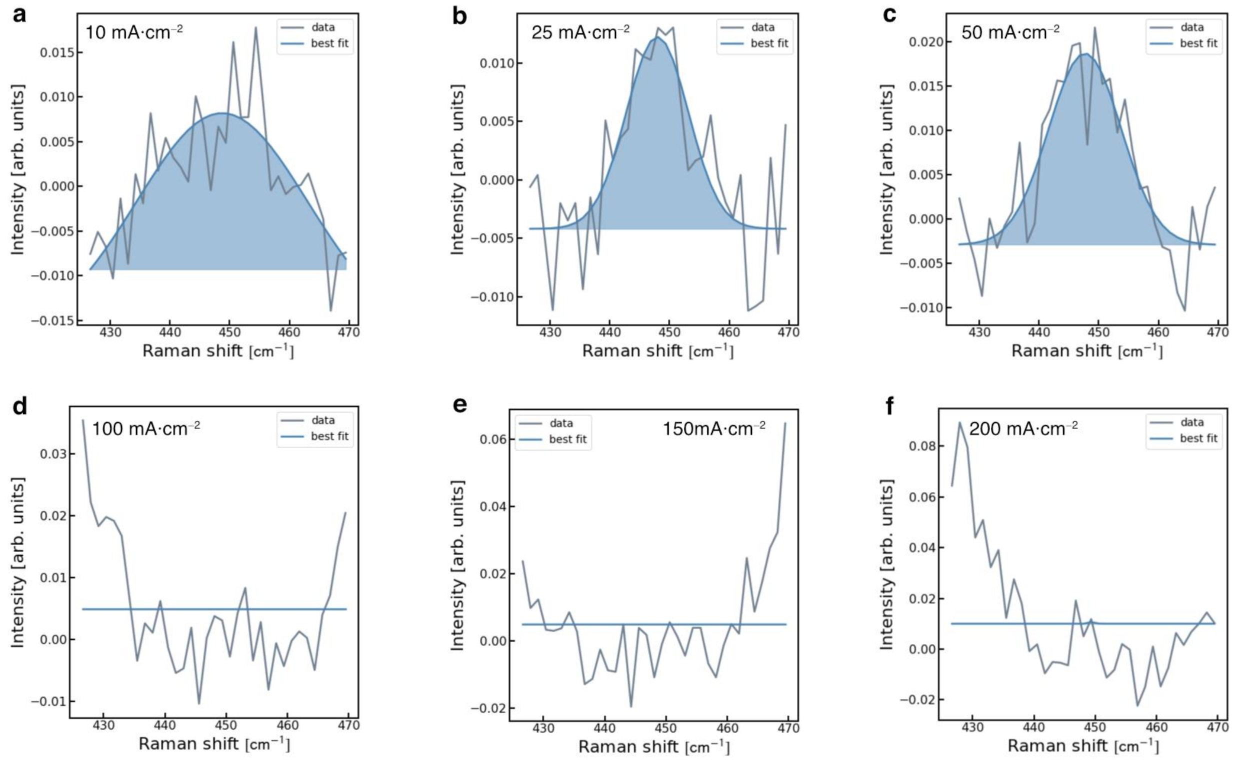

Figure S39. ***In situ*** **Raman peak fitting for OH-related band for CEI.** Peak fitting analysis of the OH vibration mode around ~450 cm^{-1} in the *in situ* Raman spectra of the CEI-coated electrode for current densities from 10 to 200 mA·cm^{-2} (**a** – **f**).

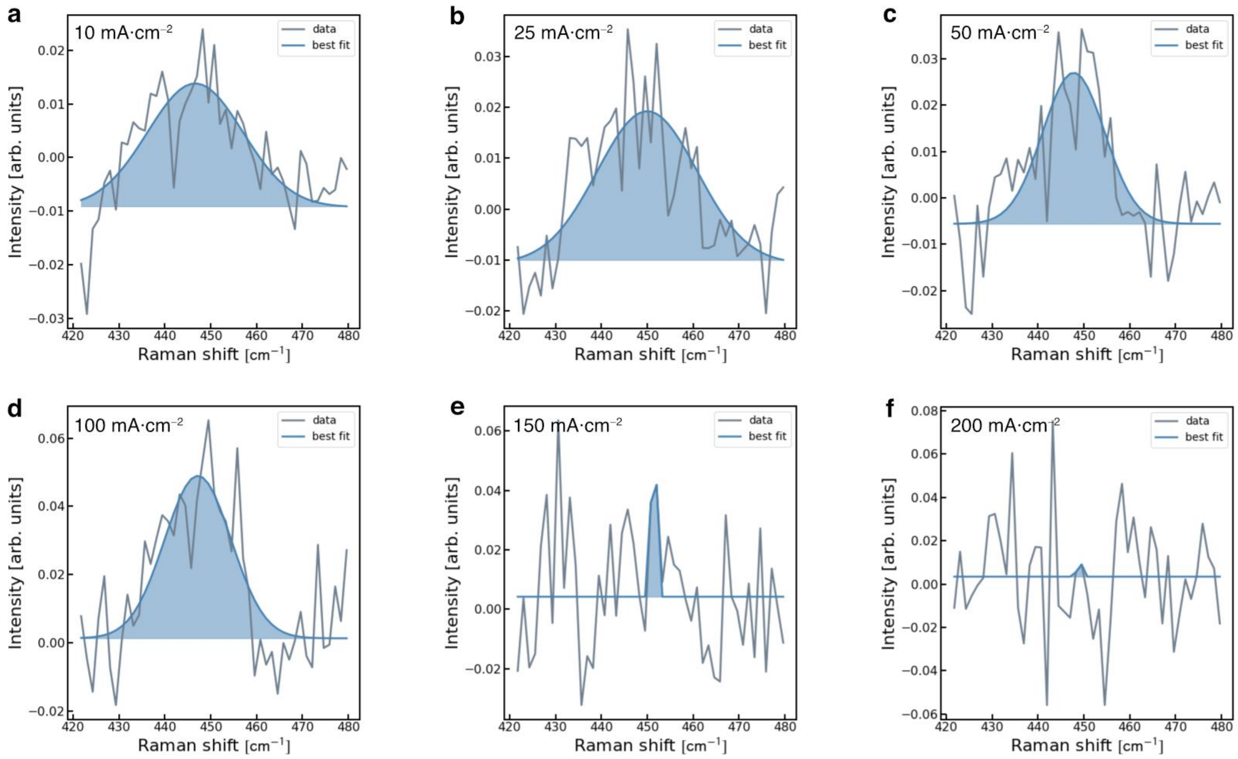

Figure S40. ***In situ*** **Raman peak fitting for OH-related band for IMC.** Peak fitting analysis of the OH vibration mode around ~450 cm^{-1} in the *in situ* Raman spectra of the IMC-coated electrode for current densities from 10 to 200 mA·cm^{-2} (**a** – **f**).

Table S6. ***In situ*** **Raman peak position and amplitude for OH peak at ~ 450 cm^{-1} for IMC and CEI.**

	**IMC**		**CEI**	
Current density [mA·cm^{-2}]	Peak position [cm^{-1}]	Peak amplitude [arb. units]	Peak position [cm^{-1}]	Peak amplitude [arb. units]
10	447	0.60	449	0.90
25	450	0.82	448	0.21
50	448	0.55	448	0.33
100	447	0.86	-	0
150	-	0	-	0
200	-	0.02		0

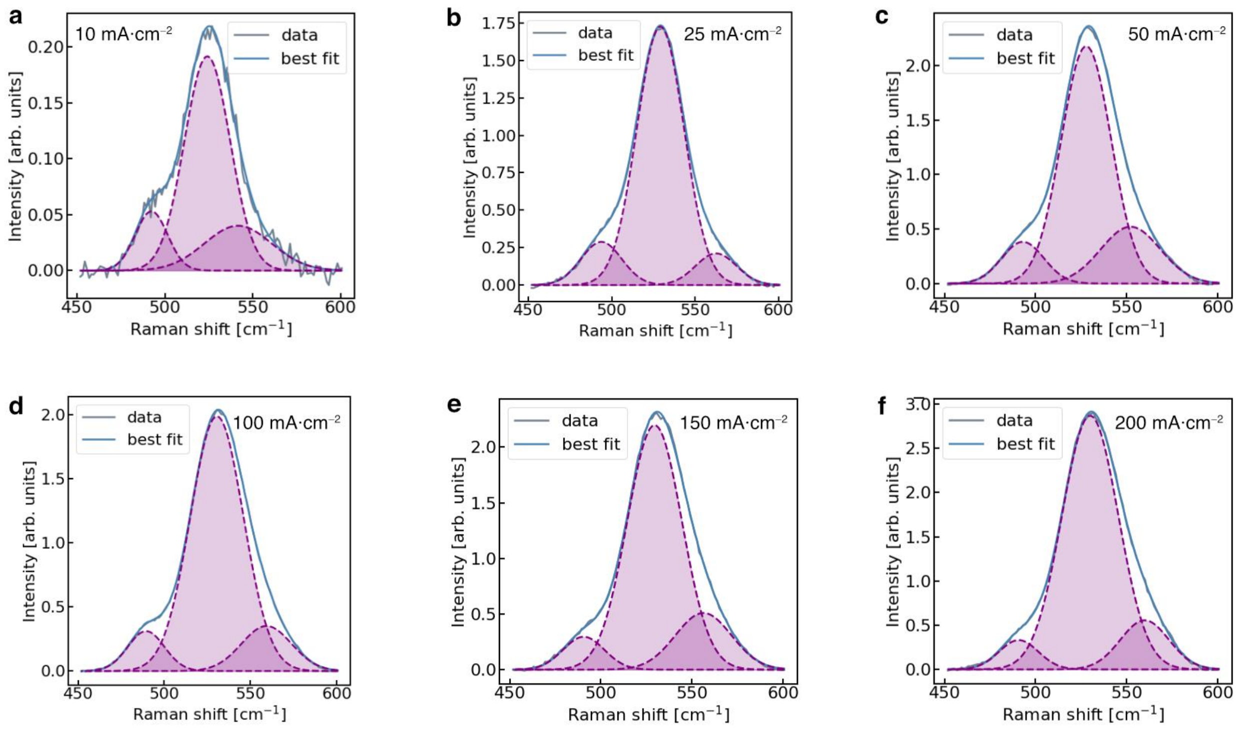

Figure S41. *In situ* Raman peak fitting for *C-inter and *OH species for CEI. Peak fitting analysis of the *in situ* Raman spectra for the CEI-coated electrode, highlighting the *C-inter (intermediate carbon species) and *OH vibrational modes for current densities from 10 to 200 $mA \cdot cm^{-2}$ (**a** – **f**).

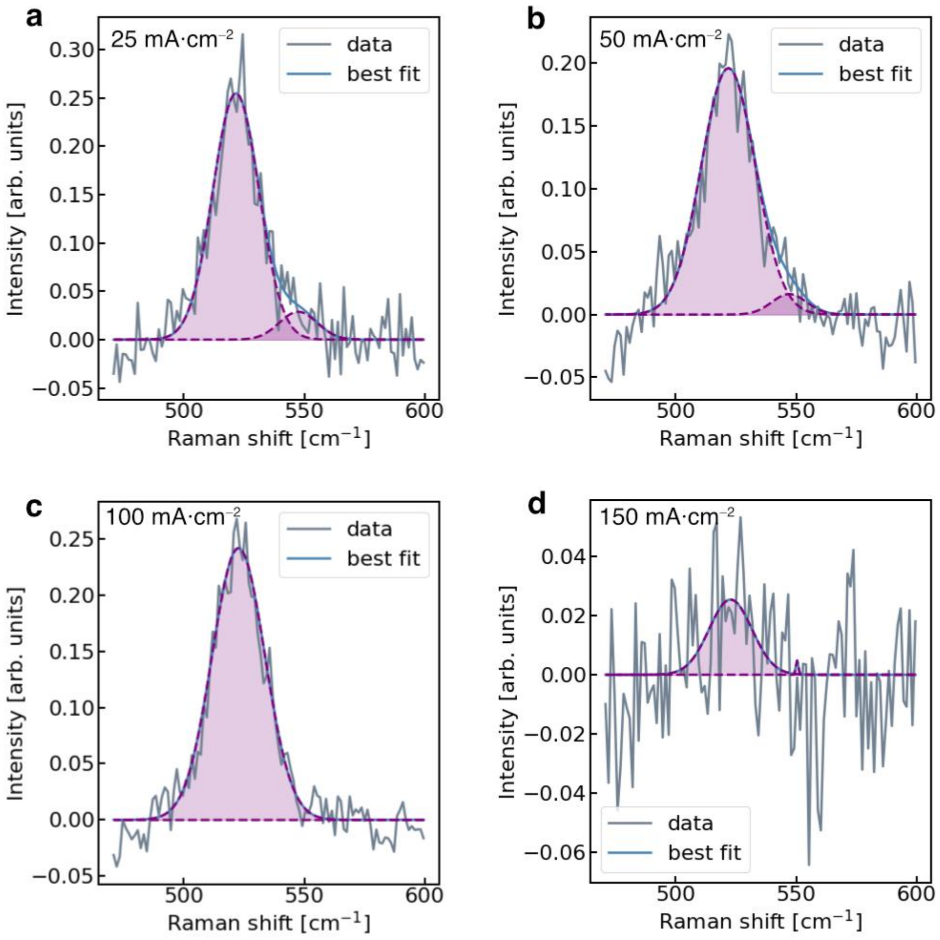

Figure S42. *In situ* Raman peak fitting for *C-inter and *OH species for AEI. Peak fitting analysis of the *in situ* Raman spectra for the AEI-coated electrode, highlighting the *C-inter (intermediate carbon species) and *OH vibrational modes for current densities from 25 to 150 mA·cm^{-2} (**a – d**). The weak signal at 150 mA·cm^{-2} limits the relevance of detailed fit accuracy.

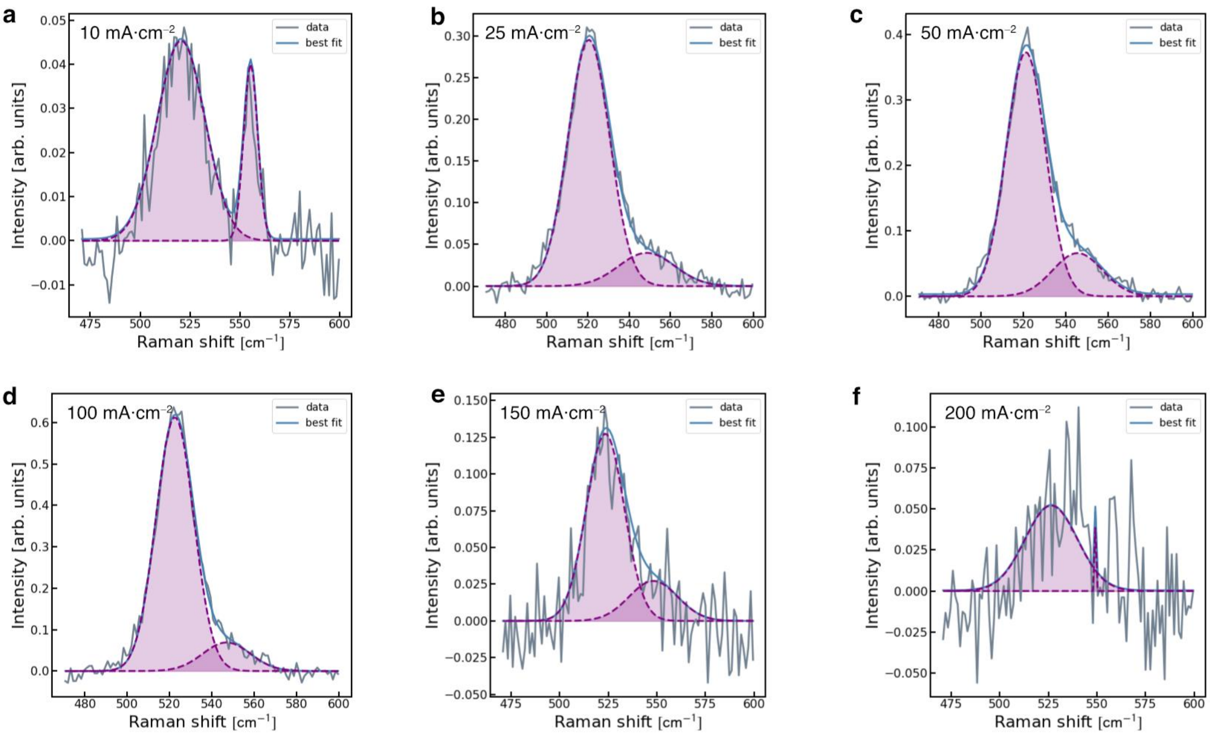

Figure S43. ***In situ*** **Raman peak fitting for *C-inter and *OH species for IMC.** Peak fitting analysis of the *in situ* Raman spectra for the IMC-coated electrode, highlighting the *C-inter (intermediate carbon species) and *OH vibrational modes for current densities from 10 to 200 mA·cm^{-2} (**a – d**). The weak signal at 200 mA·cm^{-2} limits the relevance of detailed fit accuracy.

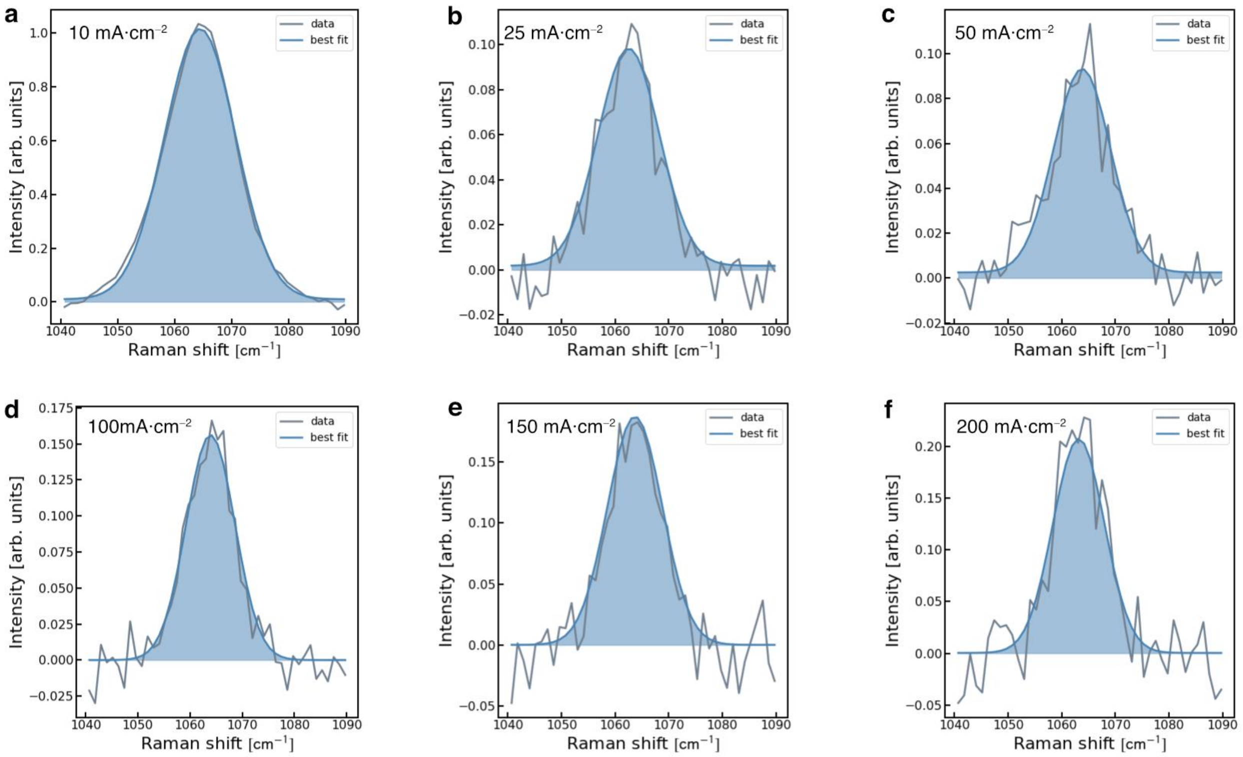

Figure S44. *In situ* Raman peak fitting for *CO_3^{2-} species for IMC. Peak fitting analysis of the *in situ* Raman spectra for the IMC-coated electrode, highlighting the * CO_3^{2-} species for current densities from 10 to 200 mA·cm^{-2} (**a – d**).

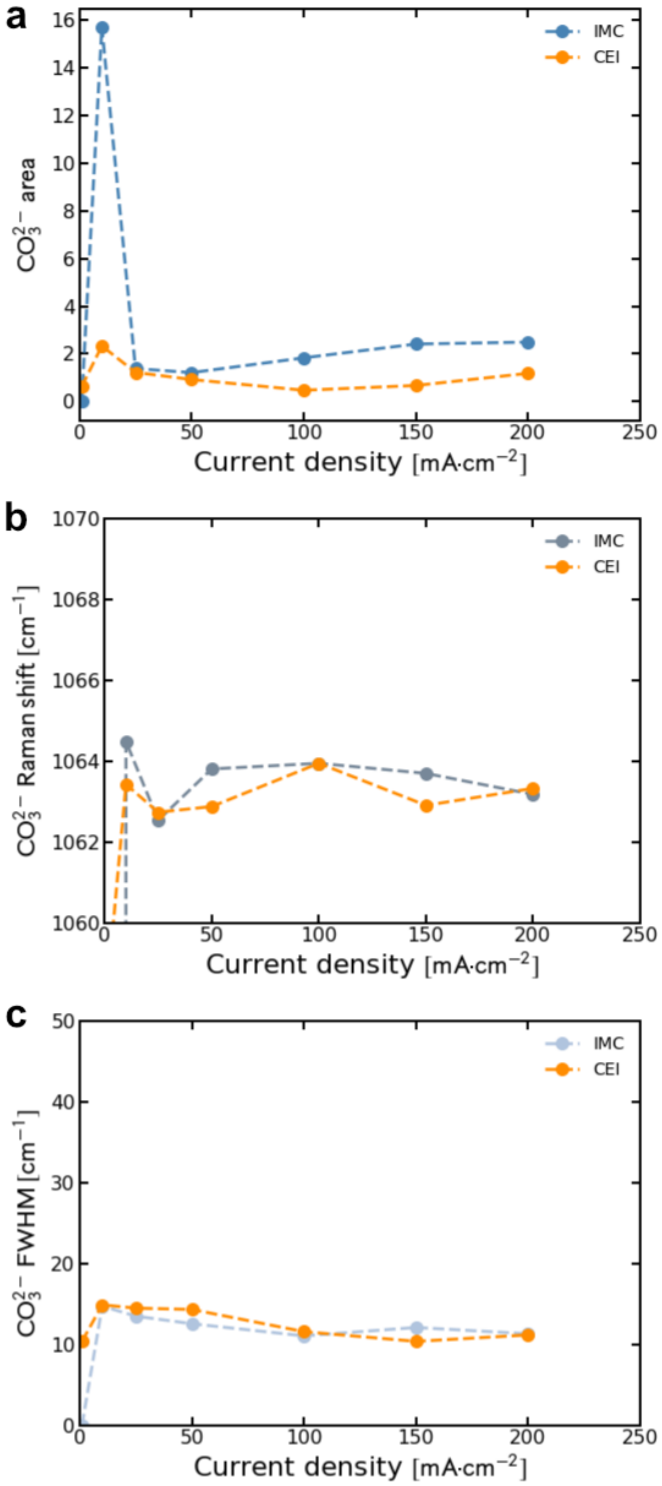

Figure S45. *In situ* Raman peak fitting results for $*CO_3^{2-}$ for IMC and CEI electrodes. Peak fitting analysis of the $*CO_3^{2-}$ vibrational mode in the *in situ* Raman spectra for IMC and CEI samples. **a** Peak area evolution, **b** Raman shift variations, and **c** full width at half maximum (FWHM) changes as a function of current density.

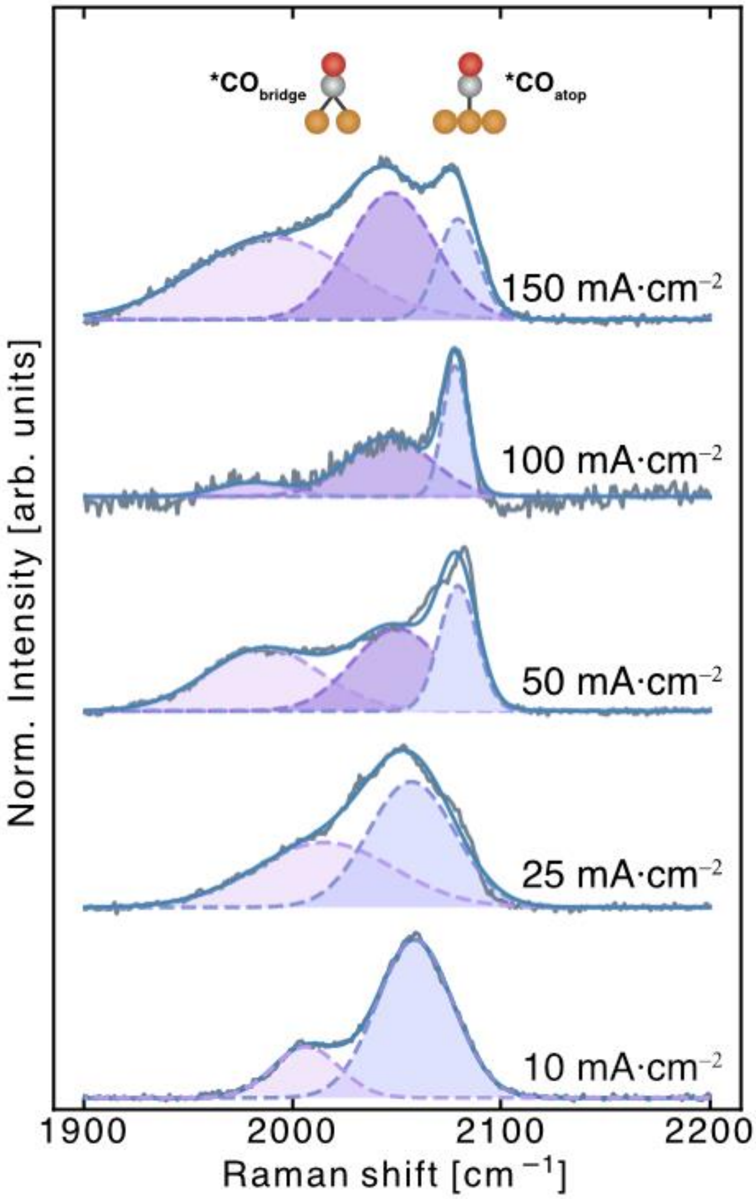

Figure S46. *COCO configuration analysis on IMC catalysts. *In situ* normalised Raman spectra and corresponding peak fittings (zoomed-in region from Supplementary Figure S26) reveal a progressive increase in the $*CO_{atop}$ adsorption mode relative to the $*CO_{bridge}$ mode as current density increases. This shift represents the preferred configuration that favours C_{2+} product formation. Measurements were conducted in a flow cell with 0.5 M K_2SO_4 + H_2SO_4 (pH = 2) under applied current densities ranging from 10 to 150 mA·cm^{-2}.

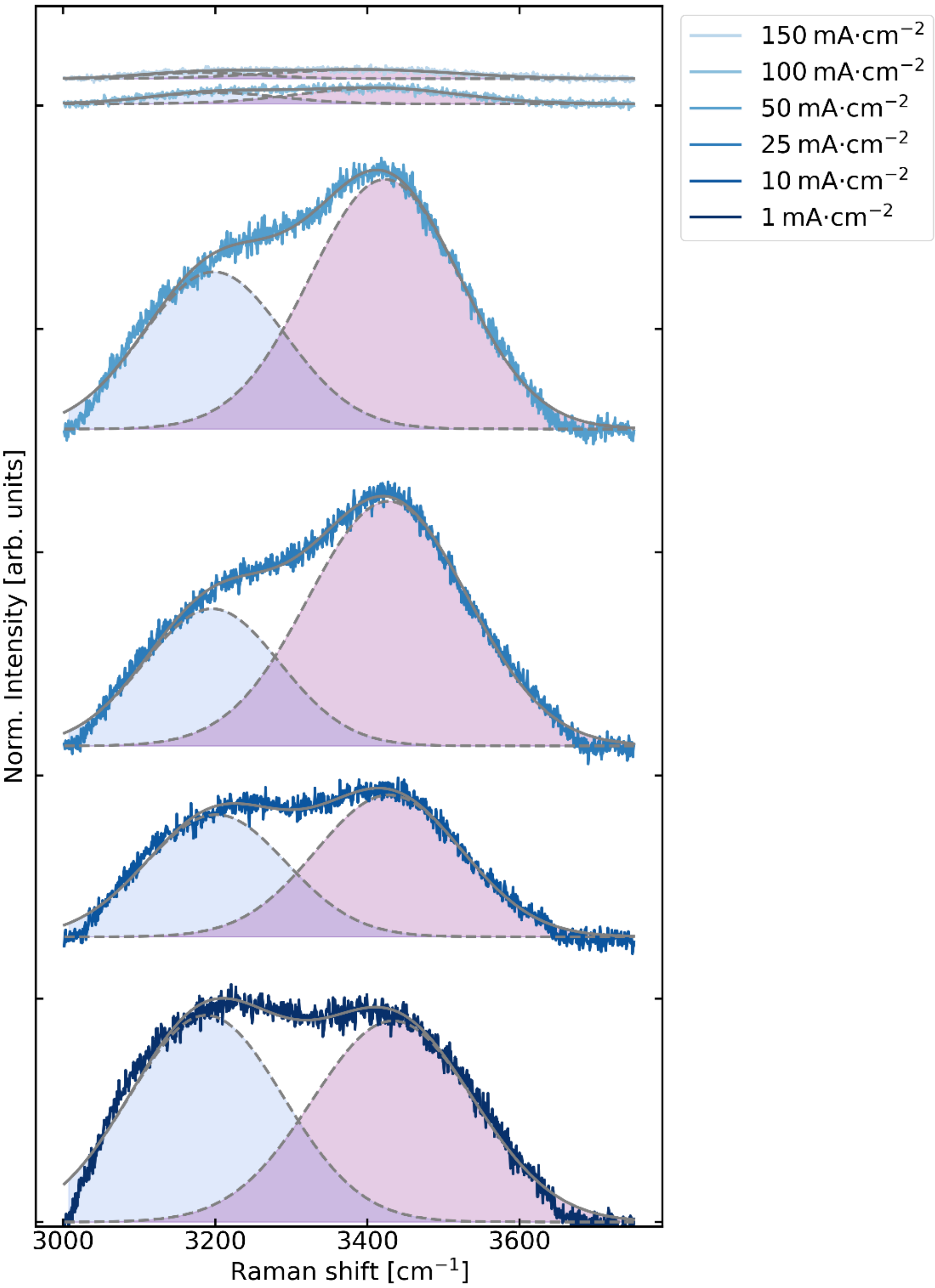

Figure S47. Interfacial structured water analysis on CEI catalysts. *In situ* normalised Raman spectra reveal two distinct hydrogen-bonded water structures at the catalyst interface: 4-HB·H_2O (blue) and 3-HB·H_2O (pink). The proportion of 4-HB·H_2O decreases significantly with applied current densities, ranging from 10 to 150 mA·cm^{-2}, indicating a shift in the interfacial water structure on CEI catalysts under electrochemical operation.

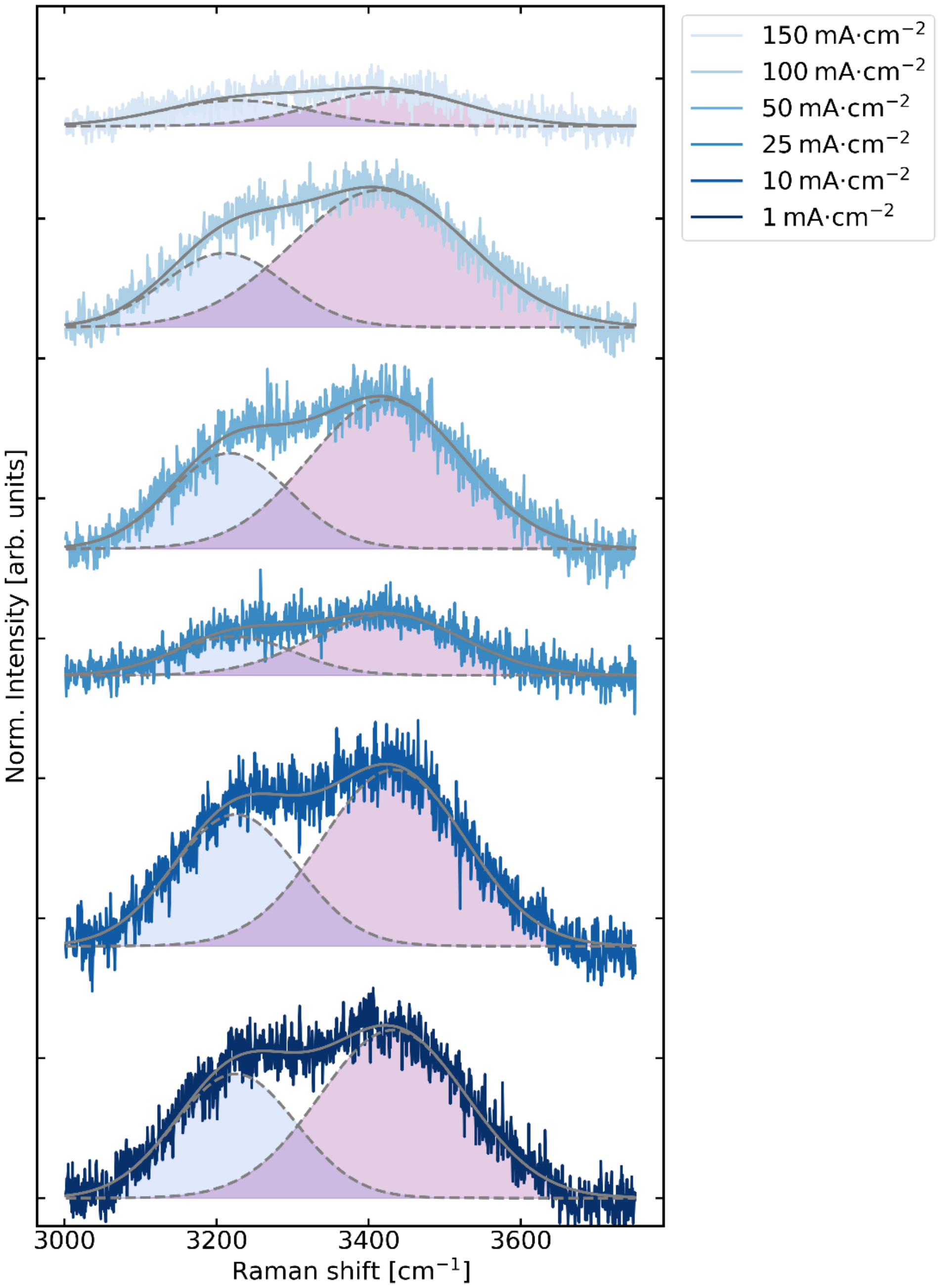

Figure S48. Interfacial structured water analysis on AEI catalysts. *In situ* Raman normalised spectra reveal two distinct hydrogen-bonded water structures at the catalyst interface: 4-HB·H_2O (blue) and 3-HB·H_2O (pink). The proportion of 4-HB·H_2O increases with applied current densities, ranging from 10 to 150 mA·cm^{-2}, indicating a shift in the interfacial water structure on AEI catalysts under electrochemical operation.

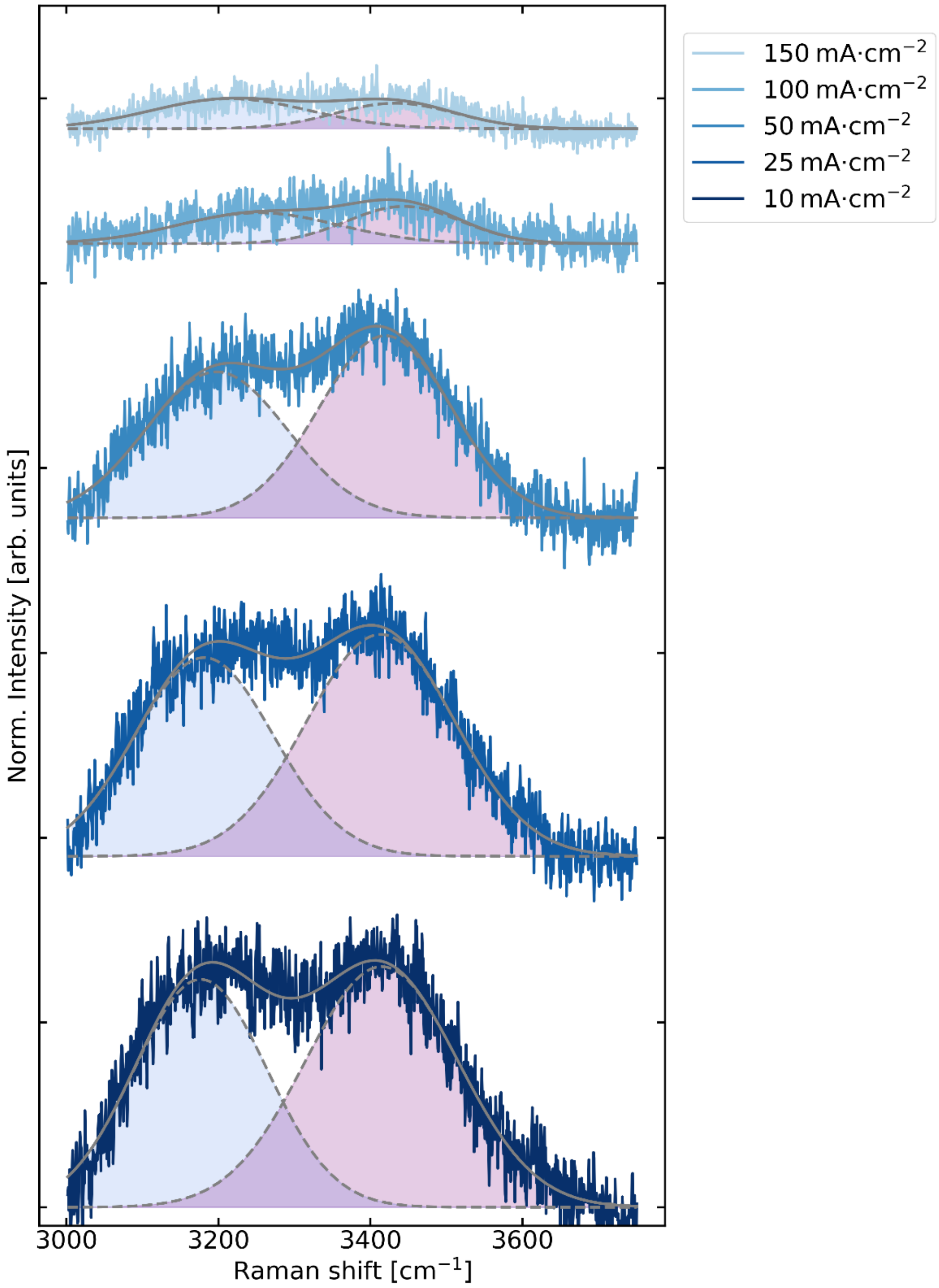

Figure S49. Interfacial structured water analysis on IMC catalysts. *In situ* Raman spectra reveal two distinct hydrogen-bonded water structures at the catalyst interface: 4-HB·H_2O (blue) and 3-HB·H_2O (pink). The proportion of 4-HB·H_2O increases significantly with applied current densities, ranging from 10 to 150 mA·cm^{-2}, indicating a shift in the interfacial water structure on IMC catalysts under electrochemical operation.

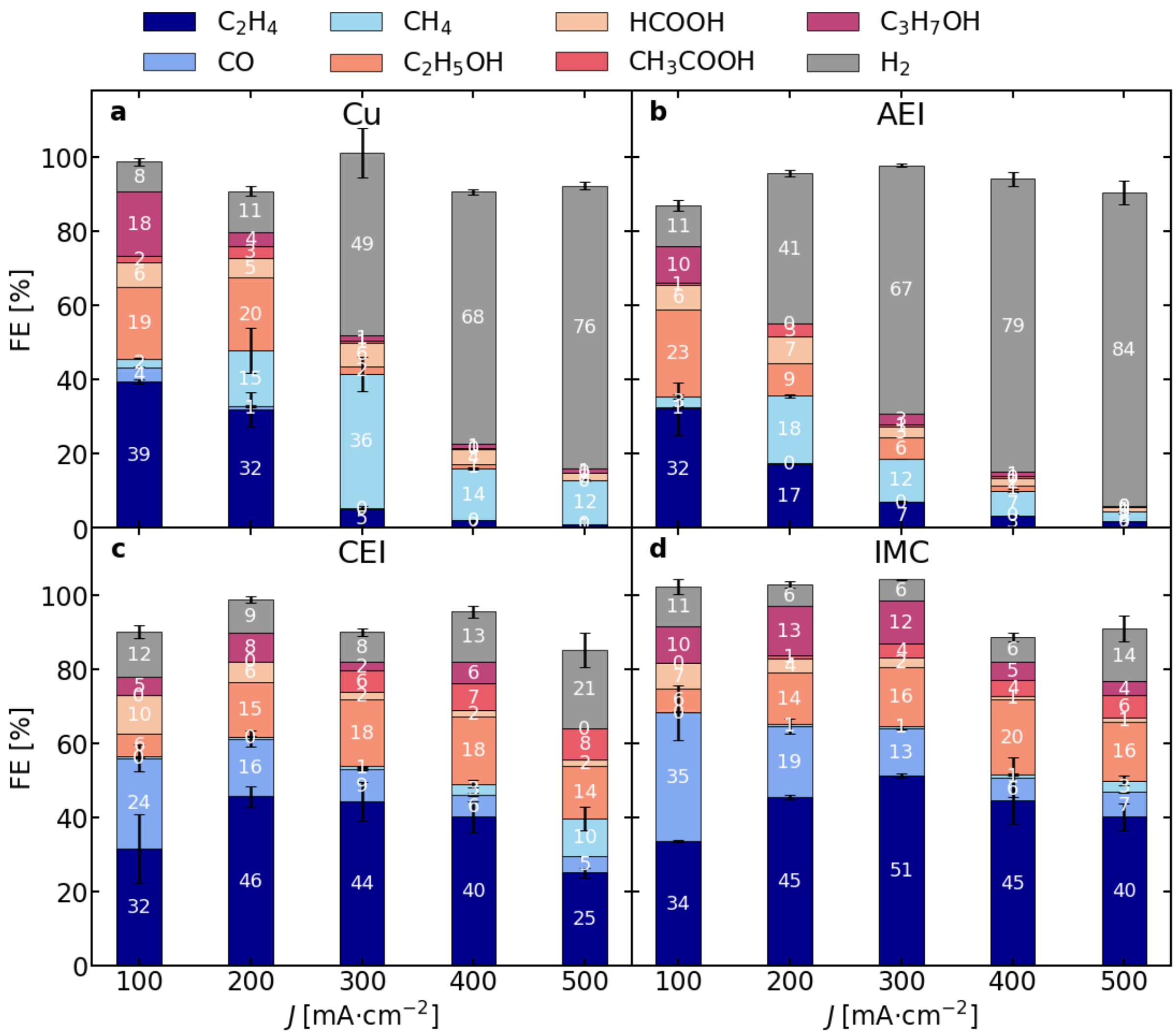

Figure S50. CO_2E performance of ionomer-coated Cu electrodes. Faradaic efficiencies of CO_2E for **a**, bare Cu **b**, AEI **c**, CEI and **d**, IMC catalysts in the presence of acidic electrolyte (0.5 M K_2SO_4 + H_2SO_4 (pH = 2)). Error bars indicate standard deviation among values from three repeated measurements. Missing products correspond to H_2 not detected in the cathode outlet, due to retention in the catholyte headspace or crossover into the anolyte compartment.

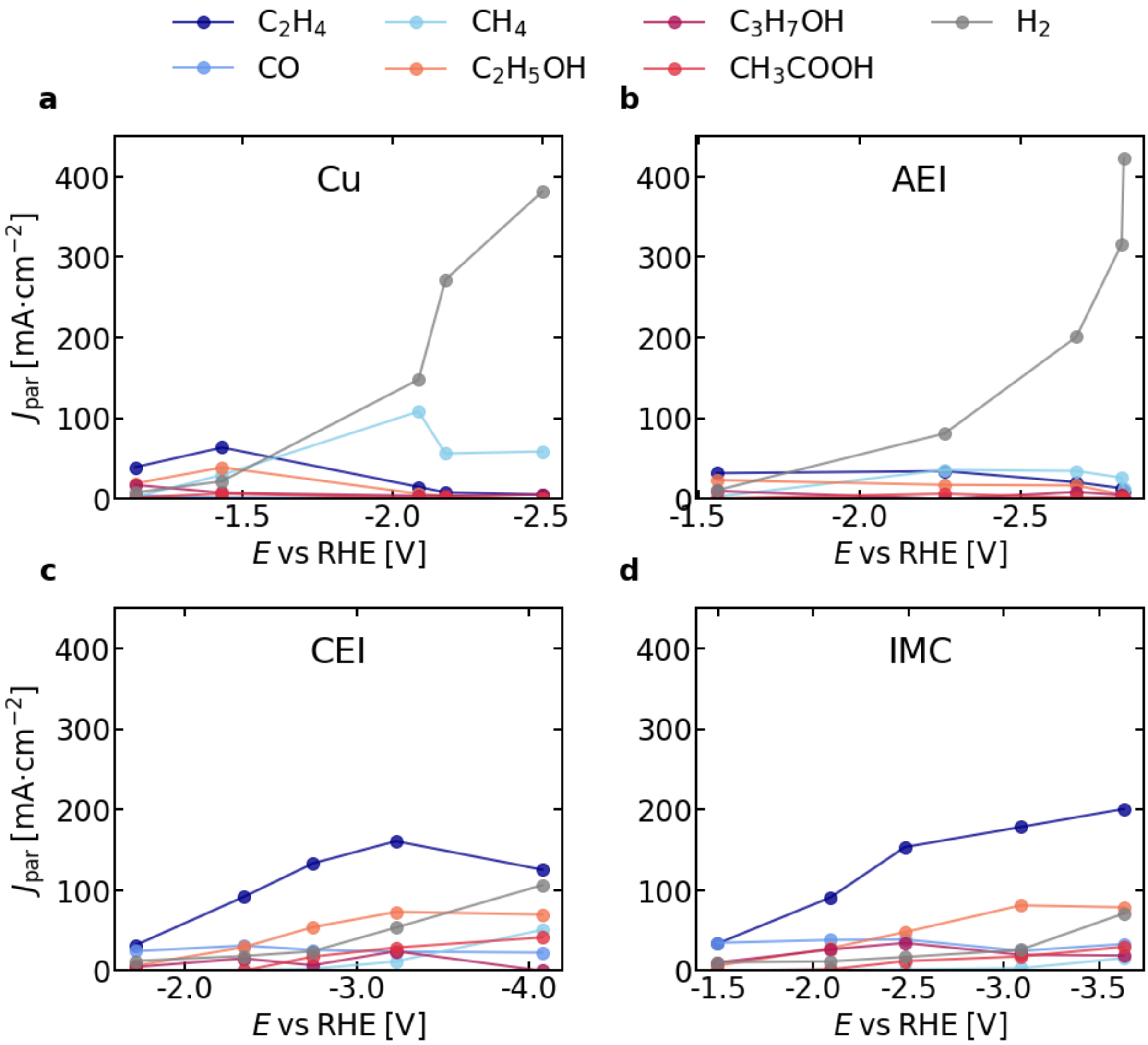

Figure S51. CO_2E performance of ionomer-coated Cu electrodes. Partial current densities of CO_2E using **a**, bare Cu **b**, AEI **c**, CEI and **d**, IMC catalysts in the presence of acidic electrolyte (0.5 M K_2SO_4 + H_2SO_4 (pH = 2)). 85% *iR* compensation was applied based on EIS measurements.

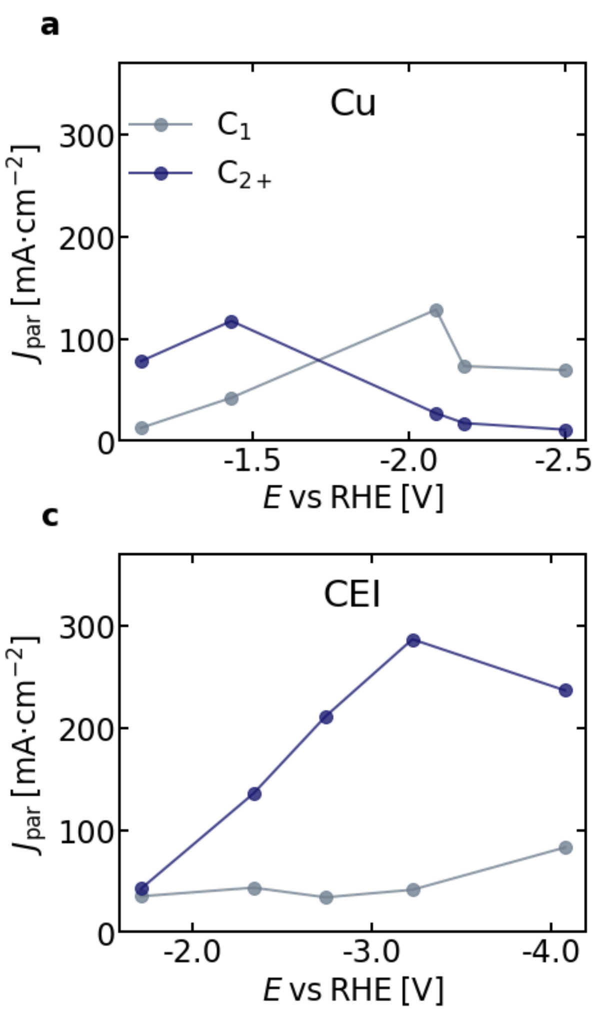

Figure S52. CO_2E performance of ionomer-coated Cu electrodes. C_1 (gray) vs C_{2+} (blue) partial current densities for **a**, bare Cu **b**, AEI **c**, CEI and **d**, IMC catalysts in the presence of acidic electrolyte (0.5 M K_2SO_4 + H_2SO_4 (pH = 2)). 85% *iR* compensation was applied based on EIS measurements.

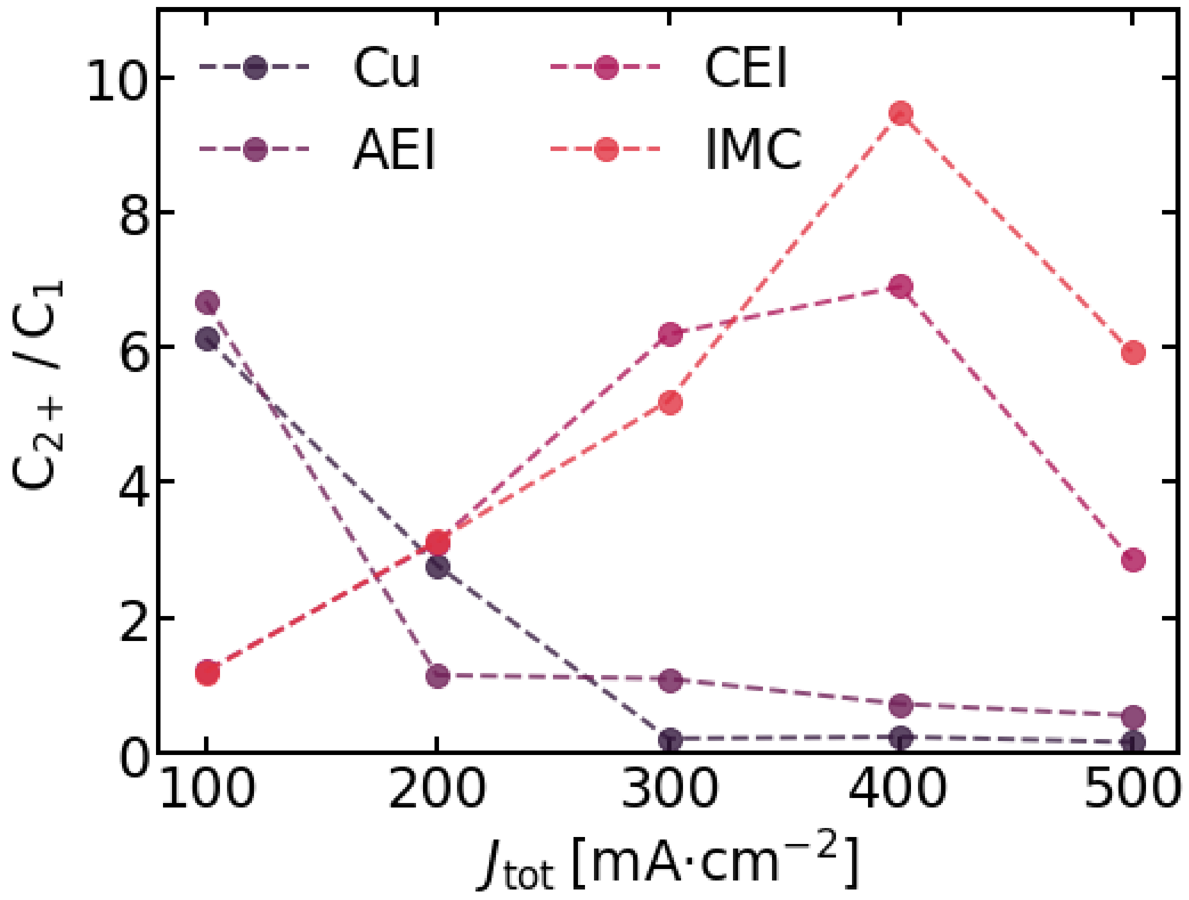

Figure S53. C_{2+}/C_1 ratio for bare Cu (dark purple), AEI (purple), CEI (violet) and IMC (orange) catalysts in the presence of acidic electrolyte (0.5 M K_2SO_4 + H_2SO_4 (pH = 2)).

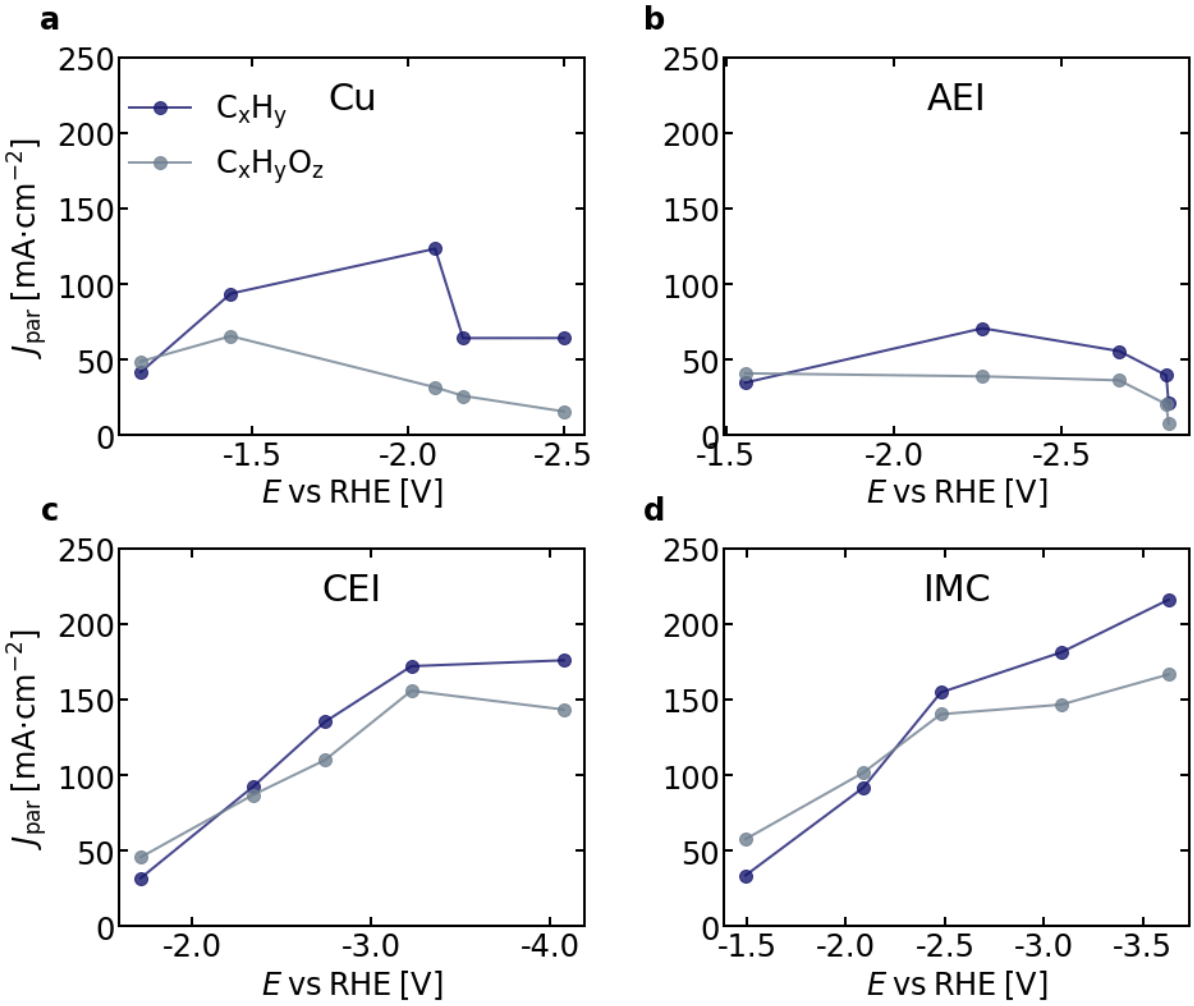

Figure S54. CO_2E performance of ionomer-coated Cu electrodes. Hydrocarbons, C_xH_y (blue) vs oxyhydrocarbons $C_xH_yO_z$ (gray) partial current densities for **a**, bare Cu **b**, AEI **c**, CEI and **d**, IMC catalysts in the presence of acidic electrolyte (0.5 M K_2SO_4 + H_2SO_4 (pH = 2)). 85% *iR* compensation was applied based on EIS measurements.

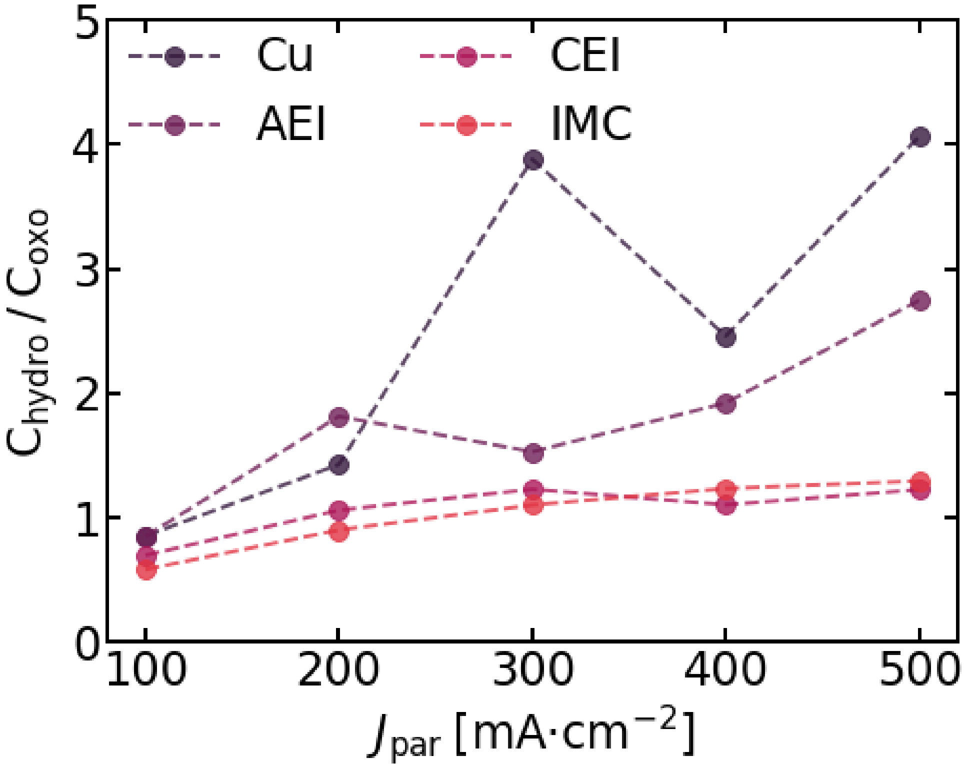

Figure S55. Hydrocarbons, C_xH_y vs oxyhydrocarbons $C_xH_yO_z$ ratio for bare Cu (dark purple), AEI (purple), CEI (violet) and IMC (orange) catalysts in the presence of acidic electrolyte (0.5 M K_2SO_4 + H_2SO_4 (pH = 2)).

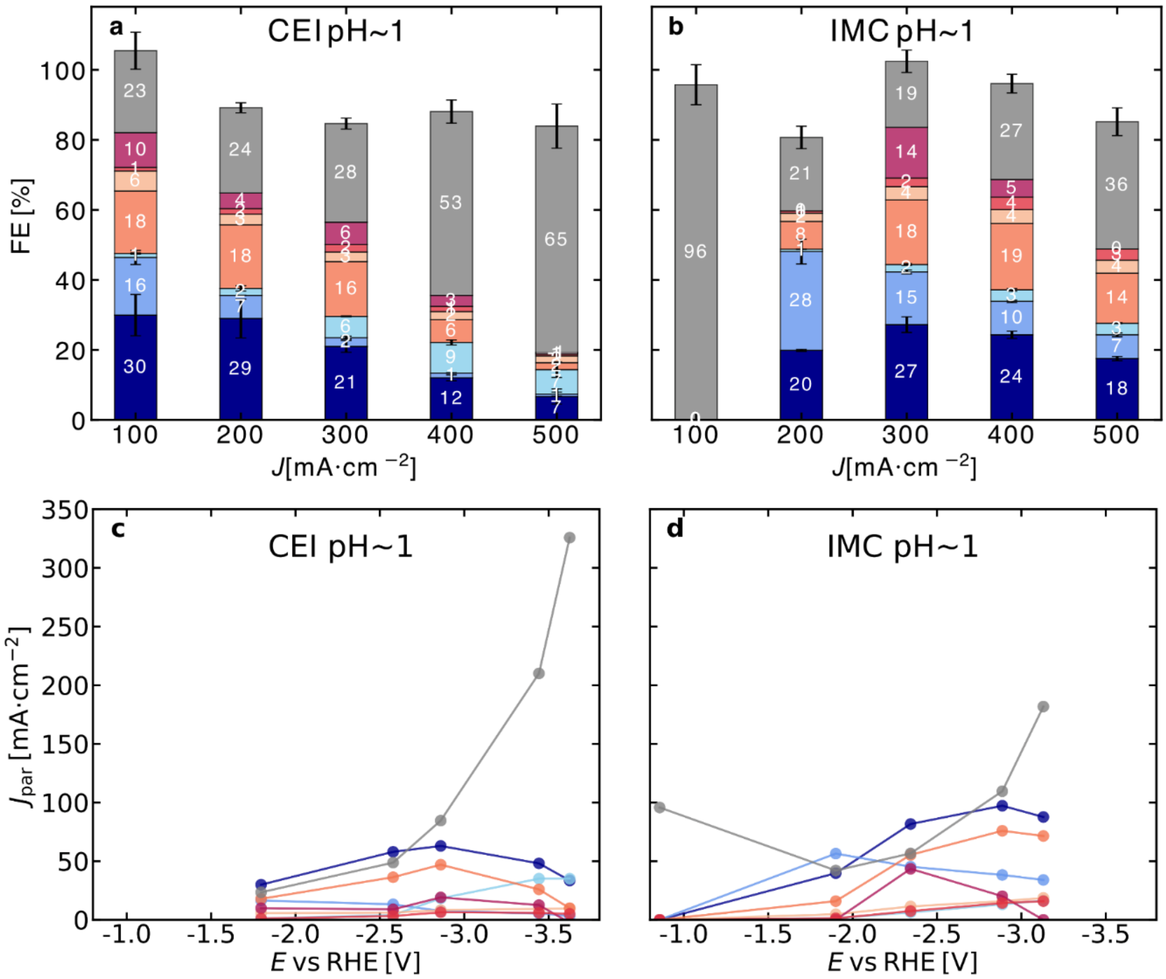

Figure S56. CO_2E performance at pH ~1 comparing CEI-only and IMC catalyst layers. Faradaic efficiencies (FEs) for major CO_2 reduction products and H_2 at total current densities of 100–500 mA·cm^{-2} for **a, c.** CEI-only and **b, d.** IMC (Aquivion + Fumion) catalyst layers. Under strongly acidic conditions, the CEI-only configuration exhibits dominant HER across the entire current range. In contrast, the IMC structure significantly suppresses H_2 selectivity while enhancing the formation of CO, C_2H_4, and other C_{2+} products, demonstrating effective ion management even at pH ~1 (K_2SO_4 0.5M + H_2SO_4 pH~1). Error bars represent standard deviation from at least three independent measurements. Missing products correspond to H_2 not detected in the cathode outlet, due to retention in the catholyte headspace or crossover into the anolyte compartment. **c, d.** Partial current densities (J_{par}) of individual products as a function of measured potential (E vs RHE). 85% iR compensation was applied based on EIS measurements. The **d.** IMC configuration achieves higher C_{2+} product current densities at lower operating potentials than the **c.** CEI-only system, reflecting reduced overpotentials and improved catalytic efficiency.

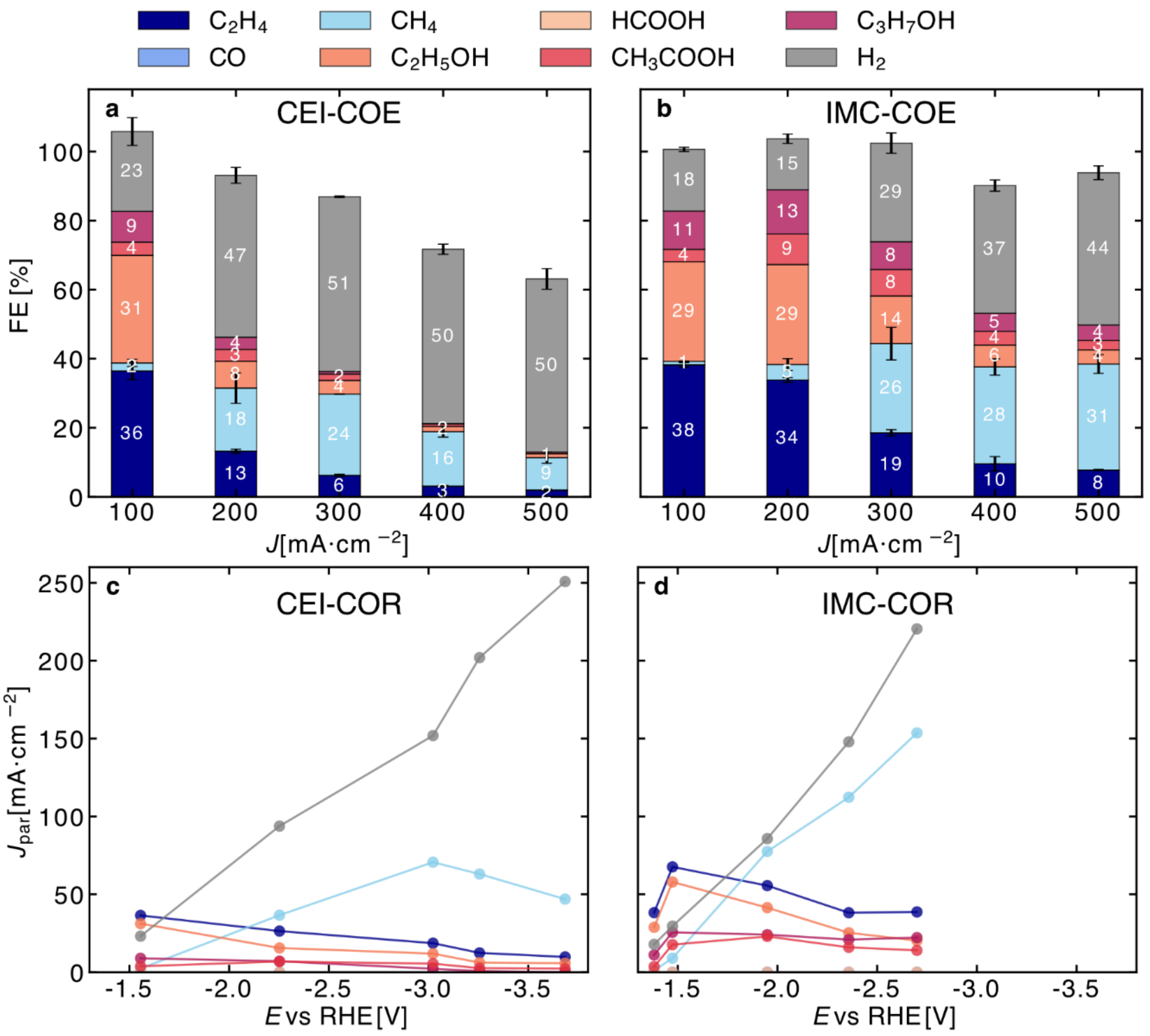

Figure S57. CO electroreduction (COE) performance between CEI-only and IMC catalyst layers. Faradaic efficiencies (FEs) of major COE products and H_2 at total current densities from 100 to 500 mA·cm^{-2} for **(a, c)** CEI-only (Aquivion) and **(b, d)** IMC (Aquivion + Fumion) catalyst layers. Both systems exhibit significant multicarbon (C_{2+}) product formation from CO feed, with the IMC configuration achieving higher selectivity for C_2H_4, C_2H_5OH and C_3H_7OH at intermediate current densities, along with moderately suppressed HER. Missing products correspond to H2 not detected in the cathode outlet, due to retention in the catholyte headspace or crossover into the anolyte compartment. Partial current densities (J_{par}) of individual COE products as a function of the measured potential (*E* vs RHE) at each applied current. 85% *iR* compensation was applied based on EIS measurements. The IMC catalyst **(d)** maintains enhanced activity for C_{2+} products relative to CEI **(c)**, while also reducing the overpotential required for CO conversion. These results confirm that the IMC strategy facilitates not only CO_2E, but also improves C–C coupling and selectivity in CO electroreduction, consistent with a favourable interfacial ionic environment.

Table S7. Performance table of state-of-art Cu-based catalysts toward C_{2+} products in acidic media.

Catalyst	Electrolyte	*FE* C_{2+} (%)	*J* (mA·cm^{-2})	J_{par} (mA·cm^{-2})	*SPCU* (%)	J_{par}× *SPCU_C*$_{2+}$ (mA·cm^{-2})	Stability (h)	Ref.
**IMC-S**	0.5 M K_2SO_4 + H_2SO_4 (pH 2)	**79.5**	**500**	**398**	**90**<br>**86.4** (C_{2+})	**343.6**	**70**	**This work**
**IMC-F**	0.5 M K_2SO_4 + H_2SO_4 (pH 2)	**82.6**	**300**	**248**	**86.5**<br>**76** (C_{2+})	**188.3**	**60**	**This work**
IMC	0.5 M K_2SO_4 + H_2SO_4 (pH 2)	62	500	310	-	-	-	This work
CEI	0.5 M K_2SO_4 + H_2SO_4 (pH 2)	68	300	204	-	-	-	This work
CEI	0.5 M K_2SO_4 + H_2SO_4 (pH 2)	47	500	235	-	-	-	This work
CAL-modified CuNP	H_3PO_4 1M / KCl 3M (pH 1)	45	1200	540	77<br>55 (C_{2+})	297	12	[20]
COF:PFSA-modified PTFE–Cu	1 M H_3PO_4 + 3 M KCl (pH 1)	75	200	150	75<br>45 (C_{2+})	67.5	20	[25]
PCRL (Cu/PTFE/ionomer)	MEA: 0.01M H_2SO_4 anolyte	30	100	30	85		8	[26]
Cu/C (carbon black)	0.1 M H_2SO_4 +0.4M K_2SO_4 (pH 1)	36	550	198	-	-	-	[27]
Modified-Cu/PTFE	1 M H_3PO_4 + 0.1M K^+ (pH 1)	55	50	27.5	-	-	5	[28]
Cu/benzimidazolium CG/CN/Nafion	0.2 M H_2SO_4 (pH 0.4)	80	100	80	90<br>78.5 (C_{2+})	62.8	150	[29]
CuNP/EmimBF$_4$	0.05M H_2SO_4+ 3M KCl (pH 0.89)	60	300	180	-	-	5	[30]
poly(Lys, Phe)-modified EC–Cu	3~8 mM $CuSO_4$, 0.05 M H_2SO_4 and 2.5 M KCl	90	200	180	70 (C_{2+})	126	10	[21]
ER-CuNS	0.05M H_2SO_4 + 3M KCl (pH < 1)	83.7	670	560	54.4 (C_{2+})	304.6	30	[19]
Pd-Cu	CO_2-saturated 0.5 M K_2SO_4	89	500	445	68<br>60 (C_{2+})	267	4.5	[31]
La-Cu hollow sphere	0.05 M H_2SO_4 + 3M KCl (pH < 1)	86.2	900	775.8	74.5<br>52.8 (C_{2+})	409.6	40	[32]
hollow-fiber Cu penetration electrode	0.05 M H_2SO_4 + 3M KCl (pH < 1)	73.4	3000	2200	51.8 (C_{2+})	1139.6	100	[33]
Cu/Polyionomer	0.5 M K_2SO_4 + H_2SO_4 (pH 2)	61	300	183	84	125.9	10	

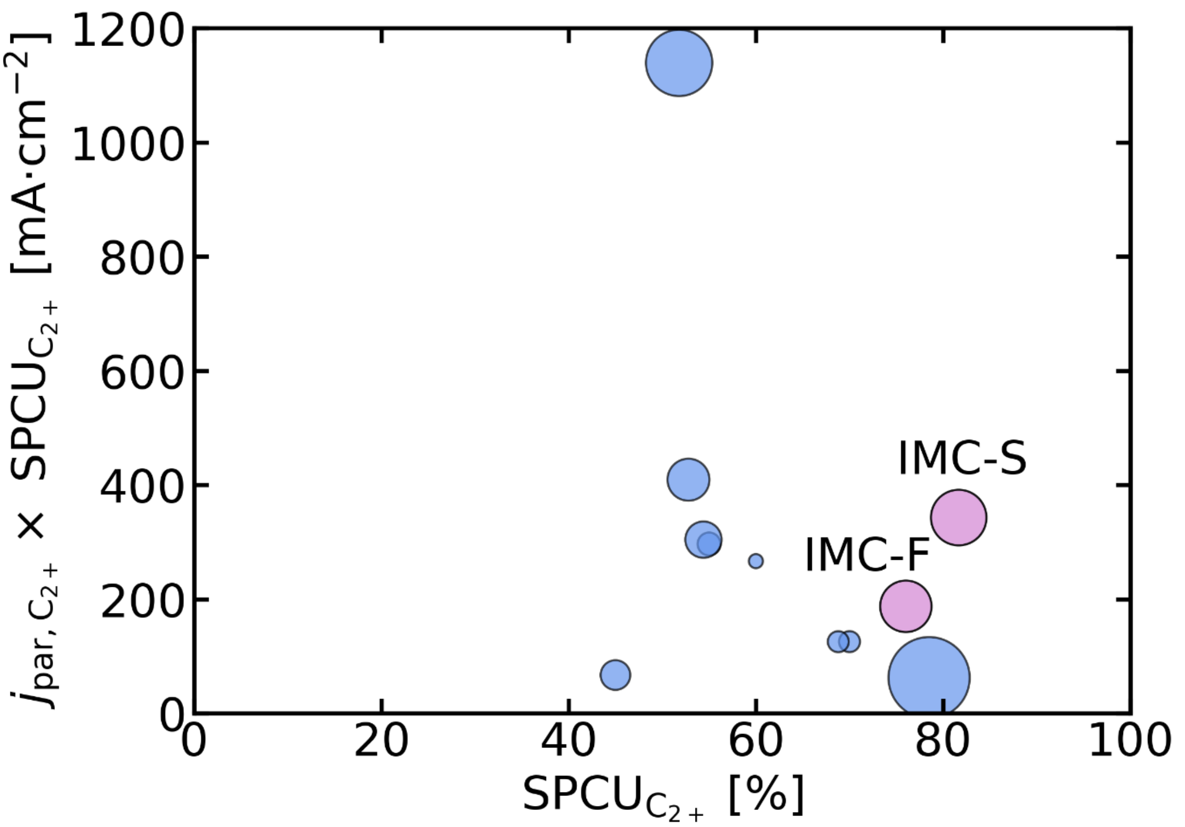

Figure S58. Performance comparison of Cu-based catalysts in acidic CO_2E. $j_{par,C2+}$ × $SPCU_{C2+}$ against $SPCU_{C2+}$ for various Cu-based catalysts in acidic CO_2E (Supplementary Table S7). The size of each data point represents the reported stability of the system. The pink data pointa corresponds to this work, demonstrating high single-pass carbon utilization towards C_{2+} products (~75% for IMC-F and ~86% for IMC-S) while maintaining a competitive effective current density (~250 mA·cm^{-2} for IMC-F and ~340 mA·cm^{-2} for IMC-S) and stability (60-70 h) relative to state-of-the-art references. This highlights the potential of the developed catalyst for efficient and scalable CO_2 conversion compared to state-of-the-art references.

Supplementary references

(1) Baek, S.-J.; Park, A.; Ahn, Y.-J.; Choo, J. Baseline Correction Using Asymmetrically Reweighted Penalized Least Squares Smoothing. *Analyst* **2014**, *140* (1), 250–257.

(2) Newville, M.; Otten, R.; Nelson, A.; Stensitzki, T.; Ingargiola, A.; Allan, D.; Fox, A.; Carter, F.; Rawlik, M. LMFIT: Non-Linear Least-Squares Minimization and Curve-Fitting for Python. *Zenodo.* **2025.** https://doi.org/10.5281/zenodo.16175987

(3) Shin, S. H.; Nur, P. J.; Kodir, A.; Kwak, D. H.; Lee, H.; Shin, D.; Bae, B. Improving the Mechanical Durability of Short-Side-Chain Perfluorinated Polymer Electrolyte Membranes by Annealing and Physical Reinforcement. *ACS Omega* **2019**, *4*, 21, 19153–19163.

(4) Vandiver, M. A.; Caire, B. R.; Pandey, T. P.; Li, Y.; Seifert, S.; Kusoglu, A.; Knauss, D. M.; Herring, A. M.; Liberatore, M. W. Effect of Hydration on the Mechanical Properties and Ion Conduction in a Polyethylene-b-Poly(Vinylbenzyl Trimethylammonium) Anion Exchange Membrane. *J Memb Sci* **2016**, *497*, 67–76.

(5) Liscio, A.; Palermo, V.; Müllen, K.; Samorì, P. Tip−Sample Interactions in Kelvin Probe Force Microscopy: Quantitative Measurement of the Local Surface Potential. *Journal of Physical Chemistry C* **2008**, *112* (44), 17368–17377.

(6) Martinez-Duarte, R.; Mondal, T. K.; Bangaru, A. V. B.; Williams, S. J. A Review on AC-Dielectrophoresis of Nanoparticles. *Micromachines* **2025**, *16*, 453.

(7) Meng, Y.; Zhou, M.; Huang, W.; Min, Y.; Shen, X.; Xu, Q. Benzyl-Containing Quaternary Ammonium Salt as a New Leveler for Microvia Copper Electroplating. *Electrochim Acta* **2022**, *429*, 141013.

(8) Titheridge, L. J.; Sharma, S.; Soisson, A.; Tiffin, C.; Roth, C.; Marshall, A. T. Investigating Cathode Ionomer Content and Assembly Techniques for Anion Exchange Membrane Water Electrolyzers. *ACS Electrochemistry* **2025**, *1* (6), 951–961.

(9) Nwabara, U. O.; Hernandez, A. D.; Henckel, D. A.; Chen, X.; Cofell, E. R.; De-Heer, M. P.; Verma, S.; Gewirth, A. A.; Kenis, P. J. A. Binder-Focused Approaches to Improve the Stability of Cathodes for CO_2 Electroreduction. *ACS Appl Energy Mater* **2021**, *4* (5), 5175–5186.

(10) Trai, N.; Chi Minh, H.; Nam Correspondence Tran Duy Tap, V.; Nam, V.; Hoang Hao, L.; Tran Trong Hieu, D.; Thanh Danh, T.; Hoang Long, T.; Truc Phuong, H.; Quang Luan, L.; Van Man, T.; Anh Tuyen, L.; Kim Ngoc, P.; Duy Tap, T. Surface Features of Polymer Electrolyte Membranes for Fuel Cell Applications: An Approach Using S2p XPS Analysis. *Science and Technology Development Journal* **2021**, *24* (3), 2100–2109.

(11) Hsu, H. L.; Leong, K. R.; Teng, I. J.; Halamicek, M.; Juang, J. Y.; Jian, S. R.; Qian, L.; Kherani, N. P. Reduction of Photoluminescence Quenching by Deuteration of Ytterbium-Doped Amorphous Carbon-Based Photonic Materials. *Materials* **2014**, *7* (8), 5643–5663.

(12) Madhuvilakku, R.; Yen, Y. K.; Yan, W. M.; Huang, G. W. Laser-Scribed Graphene Electrodes Functionalized with Nafion/Fe_3O_4 Nanohybrids for the Ultrasensitive Detection of Neurotoxin Drug Clioquinol. *ACS Omega* **2022**, *7* (18), 15936–15950.

(13) Li, L.; Tian, X.; Meng, D.; Collins, M.; Wang, J.; Patterson, R.; Nguyen, L.; Yi, X. Processing, Characterization, and Impact of Nafion Thin Film on Photonic Nanowaveguides for Humidity Sensing. *Adv Photonics Res* **2022**, *3* (2), 2100181.

(14) A.S. Bandarenka. Development of Hybrid Algorithms for EIS Data Fitting. In *Lecture Notes on Impedance Spectroscopy. Measurement, Modeling and Applications*; Ed. Kanoun, O. -CRC Press, Tailor and Francis Group: London, **2013**, *4*, 29–36.

(15) Schott, C.; Hofbauer, L.; Gubanova, E.; Schneider, P.; Bandarenka, A. S. Scanning Impedance Microscopy under Oxygen Reduction Reaction Conditions. Proof of the Concept. *Electrochim Acta* **2025**, *513*, 145533.

(16) Haimerl, F.; Kumar, S.; Heere, M.; Bandarenka, A. S. Electrochemical Impedance Spectroscopy of PEM Fuel Cells at Low Hydrogen Partial Pressures: Efficient Cell Tests for Mass Production. *Industrial Chemistry & Materials* **2024**, *2* (1), 132–140.

(17) Watzele, S.; Hauenstein, P.; Liang, Y.; Xue, S.; Fichtner, J.; Garlyyev, B.; Scieszka, D.; Claudel, F.; Maillard, F.; Bandarenka, A. S. Determination of Electroactive Surface Area of Ni-, Co-, Fe-, and Ir-Based Oxide Electrocatalysts. *ACS Catal* **2019**, *9* (10), 9222–9230.

(18) Watzele, S. A.; Kluge, R. M.; Maljusch, A.; Borowski, P.; Bandarenka, A. S. Impedance Response Analysis of Anion Exchange Membrane Electrolyzers for Determination of the Electrochemically Active Catalyst Surface Area. *Chemistry - Methods* **2024**, *4* (3), e202300035.

(19) Ma, Z.; Yang, Z.; Lai, W.; Wang, Q.; Qiao, Y.; Tao, H.; Lian, C.; Liu, M.; Ma, C.; Pan, A.; Huang, H. CO_2 Electroreduction to Multicarbon Products in Strongly Acidic Electrolyte via Synergistically Modulating the Local Microenvironment. *Nat. Commun.* **2022**, *13* (1), 1–11.

(20) Huang, J. E.; Li, F.; Ozden, A.; Rasouli, A. S.; de Arquer, F. P. G.; Liu, S.; Zhang, S.; Luo, M.; Wang, X.; Lum, Y.; Xu, Y.; Bertens, K.; Miao, R. K.; Dinh, C. T.; Sinton, D.; Sargent, E. H. CO_2 Electrolysis to Multicarbon Products in Strong Acid. *Science* **2021**, *372*, 1074–1078.

(21) Cao, Y.; Chen, Z.; Li, P.; Ozden, A.; Ou, P.; Ni, W.; Abed, J.; Shirzadi, E.; Zhang, J.; Sinton, D.; Ge, J.; Sargent, E. H. Surface Hydroxide Promotes CO_2 Electrolysis to Ethylene in Acidic Conditions. *Nat Commun* **2023**, *14* (1), 1–8.

(22) Monteiro, M. C. O.; Dattila, F.; López, N.; Koper, M. T. M. The Role of Cation Acidity on the Competition between Hydrogen Evolution and CO_2 Reduction on Gold Electrodes. *J Am Chem Soc* **2022**, *144* (4), 1589–1602.

(23) Zhan, C.; Dattila, F.; Rettenmaier, C.; Bergmann, A.; Kühl, S.; García-Muelas, R.; López, N.; Roldan Cuenya, B. Revealing the CO Coverage-Driven C-C Coupling Mechanism for Electrochemical CO_2 Reduction on Cu_2O Nanocubes via Operando Raman Spectroscopy. *ACS Catal* **2021**, *11* (13), 7694–7701.

(24) Li, Y. C.; Wang, Z.; Yuan, T.; Nam, D. H.; Luo, M.; Wicks, J.; Chen, B.; Li, J.; Li, F.; De Arquer, F. P. G.; Wang, Y.; Dinh, C. T.; Voznyy, O.; Sinton, D.; Sargent, E. H. Binding Site Diversity Promotes CO_2 Electroreduction to Ethanol. *J Am Chem Soc* **2019**, *141* (21), 8584–8591.

(25) Zhao, Y.; Hao, L.; Ozden, A.; Liu, S.; Miao, R. K.; Ou, P.; Alkayyali, T.; Zhang, S.; Ning, J.; Liang, Y.; Xu, Y.; Fan, M.; Chen, Y.; Huang, J. E.; Xie, K.; Zhang, J.; O'Brien, C. P.; Li, F.; Sargent, E. H.; Sinton, D. Conversion of CO_2 to Multicarbon Products in Strong Acid by Controlling the Catalyst Microenvironment. *Nat Synt* **2023**, *2* (5), 403–412.

(26) O'Brien, C. P.; Miao, R. K.; Liu, S.; Xu, Y.; Lee, G.; Robb, A.; Huang, J. E.; Xie, K.; Bertens, K.; Gabardo, C. M.; Edwards, J. P.; Dinh, C. T.; Sargent, E. H.; Sinton, D. Single Pass CO_2 Conversion Exceeding 85% in the Electrosynthesis of Multicarbon Products via Local CO_2 Regeneration. *ACS Energy Lett* **2021**, *6*, 2952–2959.

(27) Gu, J.; Liu, S.; Ni, W.; Ren, W.; Haussener, S.; Hu, X. Modulating Electric Field Distribution by Alkali Cations for CO_2 Electroreduction in Strongly Acidic Medium. *Nat Catal* **2022**, *5* (4), 268–276.

(28) Nie, W.; Heim, G. P.; Watkins, N. B.; Agapie, T.; Peters, J. C. Organic Additive-Derived Films on Cu Electrodes Promote Electrochemical CO_2 Reduction to C_{2+} Products Under Strongly Acidic Conditions. *Angew Chem. Int. Ed.* **2023**, *62* (12), e202216102.

(29) Fan, M.; Huang, J. E.; Miao, R. K.; Mao, Y.; Ou, P.; Li, F.; Li, X. Y.; Cao, Y.; Zhang, Z.; Zhang, J.; Yan, Y.; Ozden, A.; Ni, W.; Wang, Y.; Zhao, Y.; Chen, Z.; Khatir, B.; O'Brien, C. P.; Xu, Y.; Xiao, Y. C.; Waterhouse, G. I. N.; Golovin, K.; Wang, Z.; Sargent, E. H.; Sinton, D. Cationic-Group-Functionalized Electrocatalysts Enable Stable Acidic CO_2 Electrolysis. *Nat Catal* **2023**, *6* (9), 763–772.

(30) Vichou, E.; Perazio, A.; Adjez, Y.; Gomez-Mingot, M.; Schreiber, M. W.; Sánchez-Sánchez, C. M.; Fontecave, M. Tuning Selectivity of Acidic Carbon Dioxide Electrolysis via Surface Modification. *Chemistry of Materials* **2023**, *35* (17), 7060–7068.

(31) Xie, Y.; Ou, P.; Wang, X.; Xu, Z.; Li, Y. C.; Wang, Z.; Huang, J. E.; Wicks, J.; McCallum, C.; Wang, N.; Wang, Y.; Chen, T.; Lo, B. T. W.; Sinton, D.; Yu, J. C.; Wang, Y.; Sargent, E. H. High Carbon Utilization in CO_2 Reduction to Multi-Carbon Products in Acidic Media. *Nat Catal* **2022**, *5* (6), 564–570.

(32) Feng, J.; Wu, L.; Song, X.; Zhang, L.; Jia, S.; Ma, X.; Tan, X.; Kang, X.; Zhu, Q.; Sun, X.; Han, B. CO_2 Electrolysis to Multi-Carbon Products in Strong Acid at Ampere-Current Levels on La-Cu Spheres with Channels. *Nat Commun* **2024**, *15*, 1–11.

(33) Zhu, C.; Wu, G.; Chen, A.; Feng, G.; Dong, X.; Li, G.; Li, S.; Song, Y.; Wei, W.; Chen, W. Selective CO_2 Electroreduction to Multicarbon Products Exceeding 2 A cm^{-2} in Strong Acids via a Hollow-Fiber Cu Penetration Electrode. *Energy Environ Sci* **2024**, *17* (2), 510–517.

(34) Polesso, B.; Pinilla-Sánchez, A.; Ahmed, E. H.; Guha, A.; Dimitropoulos, M.; Belsa, B.; Golovanova, V.; Xia, L.; Ram, R.; Kadam, S.; Das, A. M.; Chen, J.; Osmond, J.; Radek

Martínez, A.; Micali, M.; Alarcón Lladó, E.; García de Arquer, F. P. Chemostructurally Stable Polyionomer Coatings Regulate Proton-Intermediate Landscape in Acidic CO_2 Electrolysis. *J Am Chem Soc* **2025**, *147* (31), 27278–27288.
